# Treatment-related modulation of visuo-vestibular integration in post-earthquake dizziness syndrome: a longitudinal virtual reality–based study

**DOI:** 10.1007/s00415-026-13767-4

**Published:** 2026-03-24

**Authors:** Hanifi Korkmaz, Işıl Çakmak Karaer, Kübra Orman, Burcu Talu, Feyza İnceoğlu

**Affiliations:** 1 Medical Health Services and Vocational School, Malatya Turgut Ozal University, Battalgazi, Malatya Turkey; 2 Department of Ear, Nose, Throat, Faculty of Medicine, Malatya Turgut Ozal University, Malatya, Turkey; 3 Department of Psychiartry, Faculty of Medicine, Malatya Turgut Ozal University, Malatya, Turkey; 4 Department of Health Science, Department of Physiotherapy and Rehabilitation, Inönü University, Malatya, Turkey; 5 Department of Biostatistics, Faculty of Medicine, Malatya Turgut Ozal University, Malatya, Turkey

**Keywords:** Post-earthquake dizziness syndrome, Visuo-vestibular integration, Sensory conflict, Virtual reality–based assessment, Vestibular rehabilitation, Cognitive behavioral therapy

## Abstract

**Background:**

Post-earthquake dizziness syndrome (PEDS) is increasingly recognized as a condition marked by persistent dizziness and imbalance after major earthquakes, often without clear peripheral vestibular pathology. Despite proposed roles of visuo-vestibular dysfunction and sensory conflict, longitudinal objective evidence remains limited.

**Objective:**

To examine the longitudinal effects of virtual reality–based vestibular rehabilitation (VR), cognitive behavioral therapy (CBT), and their integration (VR + CBT) on objective visuo-vestibular processing and symptom burden in adults with PEDS.

**Methods:**

In a four-arm longitudinal study, 48 earthquake-exposed adults with PEDS were evaluated at baseline, post-intervention, and 3-month follow-up following an 8-week intervention. Objective visuo-vestibular outcomes were assessed using an immersive virtual reality–based system, including static and dynamic subjective visual vertical (SVV/DSVV), rod-and-frame test (RFT), and visual motion sensitivity (VMS) tests. Subjective outcomes included dizziness-related handicap (DHI) and post-traumatic stress symptoms (PCL-5).

**Results:**

Dizziness-related disability and trauma-related symptoms improved over time across groups, indicating clinical modifiability of PEDS. Objective measures demonstrated a domain-specific response profile: SVV and DSVV remained largely stable, whereas RFT showed improvement in active treatment arms, suggesting reduced visual frame dependence. VMS outcomes exhibited differential trajectories, with the integrated VR + CBT group showing the most consistent and durable modulation under visually provocative conditions.

**Conclusions:**

Recovery in PEDS appears to involve selective modulation of context-dependent visuo-vestibular processing rather than uniform changes across all spatial orientation measures. Integrated VR + CBT yields the most coherent and durable benefits in visually demanding domains, supporting multidisciplinary models that jointly address sensory conflict and cognitive–emotional mechanisms after major earthquakes.

**Supplementary Information:**

The online version contains supplementary material available at 10.1007/s00415-026-13767-4.

## Introduction

Major earthquakes are complex traumatic events with lasting physical and psychological consequences that extend beyond the acute phase [1]. In addition to psychiatric outcomes such as anxiety, depression, sleep disturbances, and post-traumatic stress disorder, earthquake exposure has been consistently associated with increased dizziness, imbalance, and nonspecific vertiginous complaints. Notably, these symptoms may persist for months or years after the event and are frequently reported even in individuals without a prior history of vestibular disease [2].

Post-earthquake dizziness has been conceptualized as a distinct clinical entity and termed *post-earthquake dizziness syndrome* (PEDS) by Nomura and Toi (2014) [3]. PEDS is typically characterized by persistent sensations of disequilibrium, phantom body motion, or motion-sickness-like dizziness, often occurring in the absence of identifiable peripheral vestibular pathology. Proposed mechanisms emphasize maladaptive sensory integration processes involving visual, vestibular, and somatosensory inputs, as well as the contributory roles of psychological stress and autonomic nervous system dysregulation [2, 4]. Despite growing recognition, PEDS remains a relatively novel and undercharacterized condition, with limited empirical data on its pathophysiology, longitudinal course, and optimal management strategies.

On February 6, 2023, two catastrophic earthquakes of magnitudes 7.7 and 7.6 struck Türkiye, centered in Kahramanmaraş, affecting 11 provinces and more than 10 million people. Although nearly two years have passed, recurrent aftershocks continue to sustain a sense of instability and threat in the region. Beyond reconstruction challenges, a substantial psychosomatic burden persists, with neurology, otolaryngology, emergency medicine, and psychiatry clinics reporting ongoing presentations of dizziness, imbalance, and vertigo-like symptoms, often fluctuating with aftershock activity.

Consistent with observations following the Tohoku, Nepal, and Kumamoto earthquakes, post-earthquake dizziness complaints increased markedly after the February 2023 Türkiye earthquakes [2, 4, 5]. Recent large-scale screening studies conducted in Türkiye demonstrated strong associations between subjective dizziness severity, balance complaints, and post-traumatic stress symptoms, underscoring the close interaction between psychological distress and vestibular symptomatology in earthquake survivors [6]. However, most existing studies have relied predominantly on self-report measures and cross-sectional designs, with limited incorporation of objective assessments targeting visuo-vestibular integration or longitudinal symptom trajectories.

Experimental and clinical evidence suggests that earthquake-related dizziness arises through multiple, non-exclusive mechanisms. The otolith organs detect low-frequency linear accelerations generated during seismic activity and may disrupt normal vestibular signaling, contributing to balance disturbances and benign paroxysmal positional vertigo [2, 7]. Concurrently, psychological stress and anxiety may modulate vestibular processing at higher cortical levels, producing central patterns of postural instability and dizziness that resemble functional or perceptual disorders [8, 9]. These findings highlight visuo-vestibular integration and sensory conflict as core mechanisms in PEDS, rather than viewing the condition solely through the lens of peripheral vestibular dysfunction.

Despite increasing interest in PEDS, critical mechanistic and measurement gaps remain. Contemporary models describe PEDS as a multifactorial condition emerging from the interaction of otolithic perturbations, autonomic nervous system dysregulation, and central sensory-processing mechanisms—such as velocity storage and sensory conflict—with additional modulation by environmental instability (e.g., damaged or tilted buildings) and psychological stress [10]. Reliance on symptom inventories alone risks under-characterizing the visuo-vestibular integration processes that may sustain symptoms during recurrent aftershocks and prolonged exposure to unstable environments. Objective paradigms that directly assess spatial orientation and visual dependence—such as static and dynamic subjective visual vertical (SVV/DSVV), rod-and-frame–based measures, and visual motion sensitivity assessments—therefore provide critical insight into domain-specific signatures of sensory reweighting and conflict central to proposed PEDS mechanisms [10]. Supporting this perspective, case-based evidence demonstrates that chronic gravity-related stimulation in tilted residential environments can induce persistent dizziness and nystagmus that resolve following environmental normalization, implicating sustained otolithic loading and central integration processes beyond purely psychological explanations [11].

Importantly, while previous studies have documented the prevalence and correlates of post-earthquake dizziness, few have systematically examined treatment-related changes in objective visuo-vestibular integration parameters over time. To address these limitations, the present study investigates treatment-related changes in visuo-vestibular integration among individuals with PEDS following the February 2023 Türkiye earthquakes. By integrating objective assessments of visual–vestibular processing with longitudinal follow-up and a multidisciplinary rehabilitation framework incorporating virtual reality–based vestibular interventions, this study aims to clarify mechanisms underlying symptom persistence and recovery in earthquake-exposed populations.

The primary aim of this study was to evaluate the effects of virtual reality–based vestibular rehabilitation (VR), cognitive behavioral therapy (CBT), and their integrated application (VR + CBT) on objective measures of visuo-vestibular integration and visual dependence/sensory conflict over time (pre-treatment, post-treatment, and follow-up) in individuals diagnosed with PEDS. Specifically, the study examined treatment-related changes in SVV, DSVV, Rod-and-Frame Test outcomes, and Visual Motion Sensitivity (VMS) parameters to determine the treatment responsiveness and longitudinal stability of central visuo-vestibular integration in PEDS. It was hypothesized that patients with PEDS would exhibit significant treatment-related changes over time, from baseline to post-intervention and follow-up, in objective indices of visuo-vestibular integration and visual dependence, as reflected by measures of subjective visual vertical, dynamic subjective visual vertical, rod-and-frame performance, and visual motion sensitivity. It was further hypothesized that the integrated virtual reality and cognitive behavioral therapy intervention would produce a more consistent response profile and more durable improvements in visuo-vestibular integration and visual motion sensitivity than single-modality virtual reality, single-modality cognitive behavioral therapy, and active control conditions.

## Methods

### Study design and setting

**Fig. 1:**
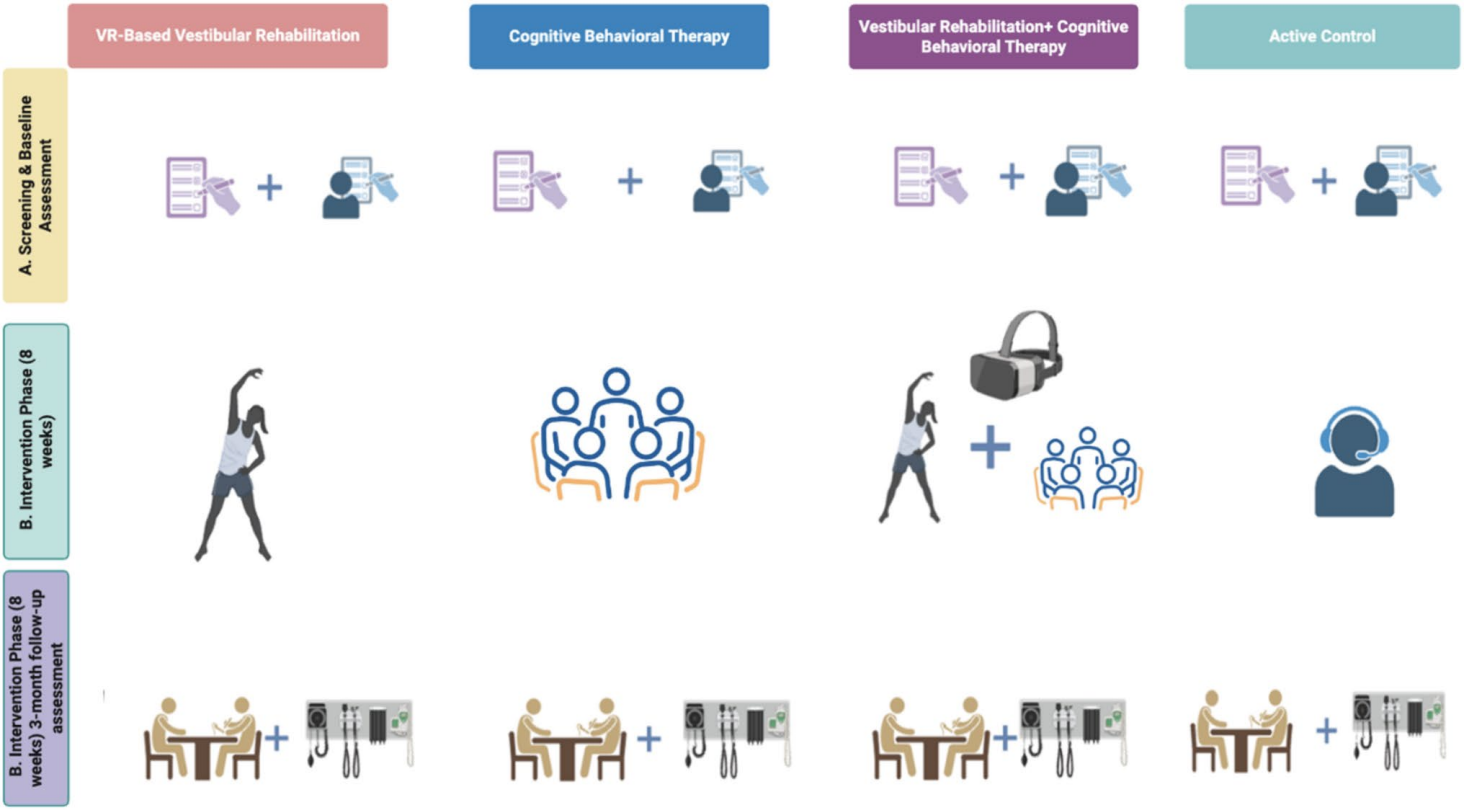
Study flow and assessment timeline illustrating baseline screening, intervention arms (VR-based vestibular rehabilitation, cognitive behavioral therapy, integrated VR + CBT, and active control), and 3-month follow-up assessments in patients with post-earthquake dizziness syndrome. All participants underwent the same baseline and follow-up assessment battery, while the intervention content differed across groups during the 8-week treatment phase

This prospective, controlled, multi-arm interventional study included adults diagnosed with post-earthquake dizziness syndrome (PEDS) following the February 2023 Türkiye earthquakes. All procedures were carried out at the Department of Otorhinolaryngology, Malatya Training and Research Hospital. Assessments were performed at baseline, immediately after completion of the 8-week intervention (post-treatment), and three months after the post-treatment assessment (follow-up). The intervention program spanned 8 weeks and consisted of 16 sessions delivered twice weekly, with an approximate duration of 90 min per session (Fig. 1). The protocol was approved by the University Clinical Research Ethics Committee (Protocol No. 2024/54) and was conducted between July 2024 and December 2025 in accordance with the Declaration of Helsinki.

Participant recruitment began in November 2024. Baseline assessments and the 8-week intervention phase (16 sessions) were completed by April 2025, including the immediate post-treatment assessment. Follow-up assessments were conducted during July–August 2025, corresponding to three months after the post-treatment assessment (i.e., after completion of the intervention). The mean interval between post-treatment and follow-up assessments across all participants was 92.4 ± 1.8 days (range: 90–95 days).

This study was conducted as a prospective, controlled, multi-arm interventional study. The initial design incorporated a randomized allocation framework. However, due to real-world post-disaster logistical and sociocultural constraints, strict randomization could not be fully implemented. Therefore, group allocation followed a pragmatic approach while aiming to approximate baseline equivalence across intervention arms.

Allocation decisions were influenced by participants’ work schedules, travel distance from container settlements to the hospital, and sociocultural considerations affecting participation in group-based CBT sessions (e.g., reluctance of some female participants to attend mixed-gender therapy groups). To minimize allocation bias, age, baseline DHI scores, PCL-5 scores, and objective visuo-vestibular measures were considered during group formation. Baseline comparability was statistically examined prior to outcome analyses. Accordingly, the study should be interpreted as a controlled, non-randomized interventional study conducted under pragmatic post-disaster conditions. Within pragmatic clinical trial frameworks, real-world constraints may limit strict randomization and experimental control while preserving clinically meaningful inference regarding intervention effectiveness in routine care settings [12]. Analytical emphasis was therefore placed on baseline comparability and group × time interaction effects rather than simple between-group contrasts.

Due to the nature of the interventions (VR-based rehabilitation, CBT, integrated VR + CBT, and structured education), blinding of participants, therapists, and outcome assessors was not feasible. Participants received clearly differentiated intervention schedules, and assessments were conducted within the same clinical workflow. However, primary objective outcomes (SVV, DSVV, RFT, and VMS parameters) were obtained using standardized, instrument-based protocols, minimizing potential assessor-related measurement bias.

### Participants and recruitment

Participants were earthquake-exposed adults residing in Malatya province who reported persistent dizziness, imbalance, or vertigo-like symptoms that emerged after the February 6, 2023, earthquakes. Recruitment was implemented through hospital-based screening of neurology, otorhinolaryngology, emergency medicine, and psychiatry outpatient records, as well as community outreach within container-city settlements established after the disaster. All participants provided written informed consent before inclusion.

### Diagnostic framework for post-earthquake dizziness syndrome

Post-earthquake dizziness syndrome was diagnosed according to the conceptual framework proposed by Nomura and Toi (2014) [3] and further elaborated in subsequent epidemiological and clinical studies [2]. PEDS was defined by the presence of persistent dizziness or illusory body motion following earthquake exposure, frequent symptom exacerbation during aftershocks, and the absence of a peripheral vestibular disorder sufficient to explain the clinical presentation fully. Particular emphasis was placed on symptom patterns consistent with central sensory conflict, visual dependence, and autonomic modulation, which are increasingly recognized as core features of PEDS. Prior to inclusion, all participants underwent a standardized bedside otoneurological examination to exclude alternative peripheral or central causes of vertigo. This assessment included positional testing for benign paroxysmal positional vertigo, bedside evaluation of spontaneous and gaze-evoked nystagmus, and the Head Impulse, Nystagmus, and Test of Skew (HINTS) examination, which is recommended for differentiating peripheral vestibular disorders from central vertigo in patients with ongoing, continuous dizziness. Individuals with clinical findings suggestive of benign paroxysmal positional vertigo, Ménière disease, vestibular neuritis, or central neurological vertigo were excluded from the study[13].

### Eligibility criteria

Inclusion criteria were: (a) dizziness and/or imbalance persisting for at least 3 months after earthquake exposure; (b) symptom aggravation during or after aftershocks; (c) moderate to severe dizziness-related disability as indicated by a Dizziness Handicap Inventory (DHI) total score ≥ 40; (d) age between 18 and 65 years; and (e) ability to participate in standing balance and virtual reality-based assessments.

Exclusion criteria included: (a) documented or self-reported vestibular disorders predating the earthquakes; (b) diagnosed neurological disease; (c) severe visual impairment limiting safe use of VR equipment; (d) cognitive impairment (Mini-Mental State Examination score < 19); (e) primary orthopedic or medical conditions affecting balance; and (f) current participation in structured vestibular rehabilitation or psychotherapy programs.

### Intervention framework

Participants were allocated to one of four intervention arms within the same clinical program: virtual reality–based vestibular rehabilitation (VR), cognitive behavioral therapy (CBT), integrated VR plus CBT (VR + CBT), or an active control condition comprising standardized education and supportive counseling. Interventions were delivered under real-world post-disaster conditions with flexible scheduling to maximize adherence while preserving protocol fidelity.

Participants allocated to the active control group did not receive VR-based rehabilitation or CBT during the intervention period. Instead, they attended structured follow-up visits and received standardized education and monitoring. Weekly follow-ups were conducted by a physiotherapist, with additional symptom monitoring by project staff. Education focused on general dizziness management, including balance safety advice, gradual increases in walking activity, Cawthorne–Cooksey exercises (positional desensitization exercises), pacing strategies, stress reduction, and lifestyle recommendations such as reduced salt intake and regular sleep patterns [14, 15]. No structured VR exposure or formal CBT was provided during the 8-week period. For ethical reasons, participants in the active control group were offered access to the integrated VR + CBT intervention after completion of follow-up assessments.

### Virtual reality-based vestibular rehabilitation

VR-based vestibular rehabilitation was delivered using an immersive balance system (BalanceVR, Virtualis, France) specifically designed for clinical assessment and rehabilitation of balance and vestibular disorders. The system integrates a head-mounted display with a force platform, enabling synchronized presentation of visual motion stimuli and real-time measurement of postural responses. This setup allows controlled manipulation of visual, vestibular, and somatosensory inputs, facilitating targeted sensory conflict and adaptive sensory reweighting.

The rehabilitation program was structured according to established vestibular rehabilitation principles, incorporating adaptation, substitution, and habituation strategies tailored to individual symptom profiles. Participants were exposed to progressively challenging visual motion environments designed to reproduce real-life situations known to provoke dizziness in PEDS, including optokinetic stimulation, visual flow environments, and dynamic scene exposure. Visual motion velocity, scene complexity, and task demands were gradually increased across sessions based on individual symptom tolerance and clinical observation, consistent with recommended VR-based vestibular rehabilitation frameworks [16].

BalanceVR provides patient-specific assessment and rehabilitation modules, enabling objective monitoring of progression across therapy sessions. Core evaluation components integrated within the system include Subjective Visual Vertical and Dynamic Subjective Visual Vertical, Rod-and-Frame paradigms, CTSIB-VR–based sensory organization testing, and cervical motion–related assessments. Rehabilitation content was selected from a range of immersive modules targeting visuo-vestibular integration and postural control, such as optokinetic environments, optical flow, target tracking, crowd simulation, escalator and elevator scenarios, head–eye coordination tasks, and sway-referenced visual environments. These modules were applied in a graded manner to enhance engagement, promote habituation to visually complex environments, and support functional recovery of balance and spatial orientation.

All rehabilitation sessions were supervised by clinicians experienced in vestibular rehabilitation, and system-generated performance metrics were recorded to document individual response patterns and guide progression throughout the intervention period.

### Cognitive behavioral therapy

Cognitive behavioral therapy was delivered in a structured group format by experienced psychiatry professionals and was administered twice weekly to participants in the CBT only and integrated VR plus CBT groups. The intervention was based on established cognitive behavioral principles, emphasizing the link between dizziness-related experiences, maladaptive beliefs, and emotional and behavioral responses. Psychoeducation addressed interactions between vestibular symptoms, anxiety, and cognitive appraisal, with sessions focusing on identifying and restructuring dysfunctional beliefs such as catastrophizing and hypervigilance. Behavioral components targeted avoidance and safety behaviors through graded exposure, supported by relaxation techniques and structured homework to reinforce cognitive and behavioral strategies. The protocol followed established CBT models for dizziness and anxiety-related conditions [17, 18].

### Integrated therapy: VR-based vestibular rehabilitation plus cognitive behavioral therapy

In the integrated VR + CBT condition, both therapeutic components were administered within the same intervention period. CBT sessions were conceptually aligned with VR exposure tasks, allowing cognitive strategies to be directly applied during visuo-vestibular challenges. This coordinated approach was designed to simultaneously target sensory conflict mechanisms and maladaptive cognitive–emotional responses.

### Visuo-vestibular outcome measures

Assessments were conducted using the BalanceVR system, an immersive virtual reality platform specifically developed for the evaluation of visuo-vestibular orientation. The system enables standardized stimulus delivery, reduces external spatial references, and integrates static SVV, dynamic SVV, and rod-and-frame tasks within a single controlled environment. Patients were tested in a seated position in a dim and quiet room, with feet unsupported to limit proprioceptive cues and minimize somatosensory contributions to verticality perception [19]. Visual stimuli were presented through a head-mounted display, and adjustments were made using a handheld joystick.

### Static subjective visual vertical (SVV)

**Fig. 2:**
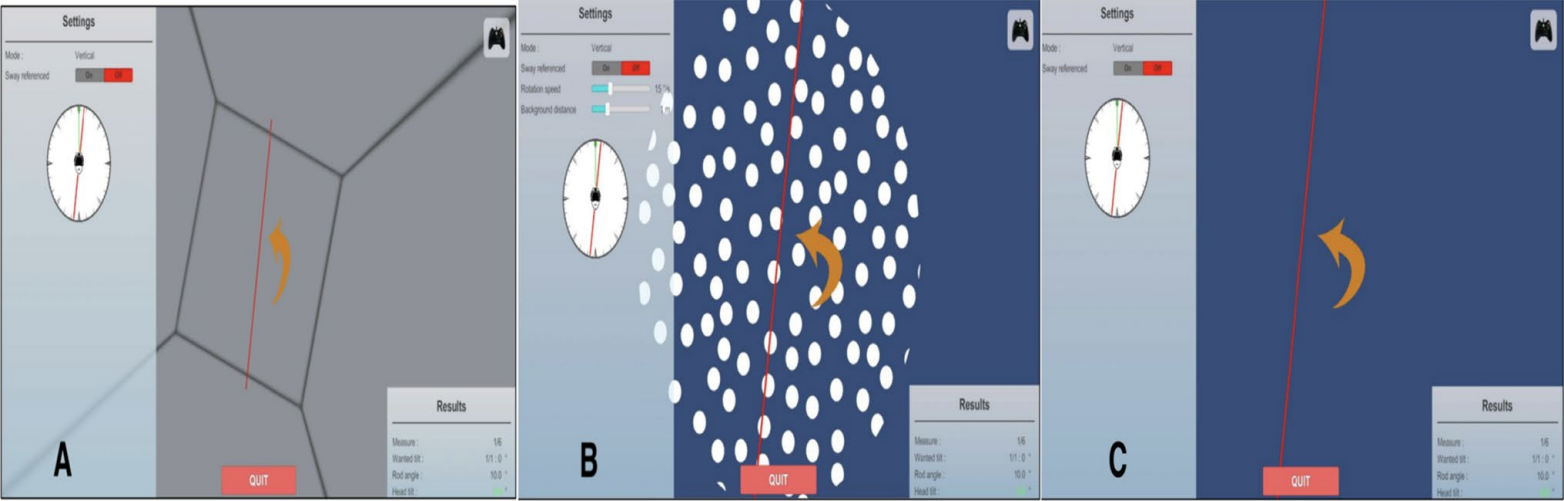
Representative screenshots of the BalanceVR test modules: **A** Rod and frame test (*RFT*). **B** Dynamic subjective visual vertical (*DSVV*), **C** Static subjective visual vertical (*SVV*)

Static SVV was assessed using the BalanceVR system (Fig. 2A). Participants were seated in a dimly lit room and viewed a single red luminous line presented on a uniform dark background through a head-mounted display (Fig. 2C). By eliminating external visual references, the task emphasized vestibular otolith contributions to verticality perception [20]. At each trial onset, the line was displayed at randomized initial orientations (± 5°, ± 10°, ± 15°, ± 20°, ± 40°). Participants adjusted the line to perceived gravitational vertical using a handheld joystick. Ten trials were completed per participant. SVV deviation was calculated as the angular difference from true vertical (0°), with clockwise deviations assigned positive values and counterclockwise deviations negative values. The VR-based setup enabled precise control of visual stimuli while limiting non-vestibular sensory cues compared with conventional bucket or dome methods. In healthy adults, static SVV deviations are typically considered within normal limits when remaining within approximately ± 2° from true vertical. Larger deviations may indicate utricular dysfunction or impaired graviceptive processing [21].

### Dynamic subjective visual vertical (DSVV)

In the DSVV condition, the static background was replaced by a field of white dots moving over a blue background, generating a controlled optokinetic stimulus (Fig. 2B). No linear edges or spatial landmarks were present, thereby preventing the use of external visual reference cues. The red luminous line was initially displayed at randomized orientations (± 5°, ± 10°, ± 15°, ± 20°, ± 40°), and participants adjusted its position to perceived gravitational vertical using a joystick. Angular deviation from true vertical was calculated, with clockwise (CW) deviations coded as positive and counterclockwise (CCW) deviations as negative. The optokinetic background rotated at a constant velocity of 30°/s; rotation direction was matched to the initial line tilt (CW for positive, CCW for negative), with trials balanced across both directions [22]. Dynamic SVV deviations are generally larger than static SVV values due to visually induced bias and are considered an index of visual dependency. VR-based dynamic SVV paradigms have demonstrated good to excellent test–retest reliability, with intraclass correlation coefficients exceeding 0.80 for clockwise and counterclockwise conditions [23].

### Rod-and-frame test (RFT)

In the VR-based RFT, patients were placed within a three-dimensional rectangular virtual enclosure that was intentionally tilted to create a misleading visual frame (Fig. 2A). The frame was oriented at ± 10°, ± 20°, or ± 30° relative to the gravitational vertical. Across ten trials, a red luminous line was presented at randomized initial angles, and participants adjusted its orientation using a joystick until it was perceived as vertically aligned in real space, while disregarding the tilted surrounding frame. The angular deviation from true vertical served as an index of visual dependence and reliance on contextual visual cues. By situating the classical rod-and-frame paradigm within an immersive three-dimensional environment, this VR implementation enhances ecological validity and perceptual engagement while maintaining strict control over external spatial references [24].

### Visual motion sensitivity

Visual motion sensitivity was assessed using a full-field optokinetic paradigm within an immersive virtual reality environment combined with force-platform recording. Participants stood barefoot on the StaticVR platform in a standardized position while exposed to rotating white dots filling the visual scene, inducing a controlled visual–vestibular sensory conflict. This paradigm was designed to challenge visual dependency and visuo-vestibular integration mechanisms commonly implicated in chronic dizziness conditions such as PEDS.

During testing, participants were instructed to maintain an upright and stable posture without voluntary movement, allowing involuntary postural responses elicited by visual motion to be captured objectively. The assessment focused on optokinetic- and optic-flow–related sway metrics reflecting the efficiency of sensory reweighting under conditions of visual motion. Experimental work has demonstrated that immersive visual motion environments reliably modulate center-of-pressure (CoP) velocity and sway area, reflecting dynamic sensory reweighting processes (Teaford et al., 2023; Keshner & Kenyon, 2020). In this context, instrumented VMS outcomes provide objective indices of altered visuo-vestibular integration beyond subjective symptom reporting [25].

VMS metrics were derived from synchronized force-platform recordings during optokinetic and optical-flow stimulation. The software provides condition-specific center-of-pressure (CoP) displacement speed, displacement amplitude, and confidence ellipse surface measures, computed from CoP trajectories over time according to the manufacturer’s processing pipeline [26].

Because standardized normative thresholds for VR-based VMS metrics are limited in the literature, device-specific reference data were obtained from an independent healthy cohort (*n* = 50; 28 women, 22 men) assessed with the identical protocol during the project standardization phase. For each VMS parameter, reference distributions were summarized as mean ± SD and 5th–95th percentile ranges. These values were used to contextualize baseline performance and longitudinal change, without applying diagnostic cut-offs. These normative data allow interpretation of baseline deviation from expected performance ranges, thereby partially addressing the absence of a parallel healthy control arm.

Given that optokinetic and optical-flow stimuli may induce habituation across repeated exposures, potential repeated-testing effects were considered in the study design. All groups underwent identical assessment schedules, and longitudinal analyses were based on group × time interaction models rather than simple within-group change. These considerations reduce the likelihood that observed differences reflect mere habituation to visual motion stimuli rather than intervention-related effects.

### Instrumented visual motion sensitivity metrics: computation, reference framework, and interpretive criteria

During VMS assessment, optokinetic speed (°/s) and optical flow speed (m/s) were predefined stimulus parameters set within the software environment and delivered as controlled visual perturbations. These parameters represent stimulus intensity rather than postural response outputs. Outcome metrics were derived from synchronized force-platform–based center-of-pressure (CoP) trajectories recorded during these visual perturbations. The software provides condition-specific CoP displacement speed (cm/s), displacement amplitude (cm), and 95% confidence sway ellipse area (cm^2^), computed from time-series CoP data according to the manufacturer’s processing pipeline [26]. The 95% sway ellipse represents the area enclosing 95% of CoP samples during the trial and is a widely accepted descriptor of postural stability in conventional and virtual reality–based posturography systems. In the present system, optokinetic and optic-flow exposure are delivered as full-field visual perturbations; no independent ‘optokinetic area’ output metric is generated. Therefore, the visual stimulation area corresponds to the full immersive field defined by the VR environment.

Optokinetic and optic-flow stimuli function as controlled sensory perturbations that increase visual weighting and induce visuo-vestibular conflict. Immersive visual surround–based posturography systems have been shown to yield repeatable sway measures comparable to conventional dynamic posturography paradigms (Trueblood et al., 2018). In line with established sensory reweighting models [27], CoP speed and ellipse area were interpreted as objective markers of visual weighting under motion-induced sensory conflict.

Given the limited availability of standardized normative thresholds for VR-based VMS parameters, device-specific reference data from an independent healthy cohort (*n* = 50) assessed with the identical protocol during the standardization phase were used to contextualize performance. Reference distributions (mean ± SD; 5th–95th percentiles) were applied as interpretive anchors rather than diagnostic cut-offs. Higher CoP speed and sway ellipse area relative to reference ranges were considered indicative of increased visual dependence, whereas shifts toward reference values were interpreted as improved sensory reweighting capacity, consistent with established postural control models [27], within an immersive VR context.

Given that repeated exposure to optokinetic and optic-flow stimulation may induce habituation effects, longitudinal interpretation was based primarily on group × time interaction effects rather than isolated within-group change. All study arms underwent identical assessment schedules, reducing systematic bias related to repeated testing. This analytic strategy minimizes the likelihood that observed changes reflect nonspecific habituation to visual motion stimuli rather than intervention-related modification of visuo-vestibular integration in PEDS.

### Subjective outcome measures

#### Dizziness handicap inventory

Dizziness-related disability was evaluated using the Dizziness Handicap Inventory, a widely used 25-item self-report questionnaire designed to quantify the perceived impact of dizziness on daily functioning (Jacobson and Newman, 1990). The DHI includes physical, functional, and emotional subdomains addressing symptom provocation, limitations in daily activities, and emotional responses associated with dizziness. The validated Turkish version of the DHI was administered and has demonstrated good internal consistency, test–retest reliability, and construct validity in individuals with vestibular disorders [28]. Both total and subscale scores were calculated according to standard scoring guidelines.

#### Post-traumatic stress symptoms

Post-traumatic stress symptoms were assessed using the Posttraumatic Stress Disorder Checklist for DSM-5 (PCL-5), a 20-item self-report instrument developed to measure the severity of DSM-5–defined PTSD symptoms over the preceding month [29]. The PCL-5 evaluates symptom clusters related to intrusion, avoidance, negative alterations in cognition and mood, and hyperarousal, with items rated on a 5-point Likert scale (0 = not at all to 4 = extremely). Total scores range from 0 to 80, with higher scores reflecting greater overall PTSD symptom burden. The Turkish validated version of the PCL-5 was used, which has shown strong psychometric properties, including high internal consistency and convergent validity, in trauma-exposed populations [30]. Total scores were used for analyses to characterize global post-traumatic stress severity.

### Statistical analysis

The primary outcome was predefined as the group × time interaction effect for VMS optical flow mean speed, selected as the most direct objective marker of visually induced sensory conflict modulation. Secondary objective outcomes included other VMS parameters and RFT measures. Exploratory/secondary outcomes included SVV, DSVV, DHI, and PCL-5.

Statistical analyses were performed using SPSS software (Statistical Package for the Social Sciences), version 28.0. The distributional properties of continuous variables were examined using the Kolmogorov–Smirnov test. As all primary outcome measures demonstrated normal distribution (*p* > 0.05), parametric statistical methods were applied throughout the analyses. The level of statistical significance was set at *p* < 0.05.

To evaluate treatment-related changes over time in visuo-vestibular integration measures, repeated-measures analyses of variance were conducted. Specifically, a two-way mixed-design analysis of variance (group × time) was used to examine whether changes across assessment points (baseline, post-intervention, and follow-up) differed between intervention groups. When significant main effects or interaction effects were detected, post-hoc pairwise comparisons were performed using Bonferroni correction to control for multiple testing.

Assumptions of sphericity and homogeneity of variances were examined and confirmed prior to model interpretation. For between-group comparisons at individual time points, one-way analysis of variance was applied, followed by Bonferroni-adjusted post-hoc tests when appropriate. Categorical variables were analyzed using cross-tabulations and chi-square tests. Where appropriate, effect sizes were reported to support the interpretation of the magnitude of group and time effects. A priori sample size calculation was performed using G*Power 3.1 [31] for a repeated-measures within–between interaction design. Assuming an effect size of *f* = 0.35, *α* = 0.05, power (1–β) = 0.95, four groups, and three measurement points, the required total sample size was calculated as *n* = 48. The final sample met this requirement. (See sup.1).

## Results

A total of 48 patients diagnosed with PEDS were included in the study. No significant differences were observed among the groups in terms of age, indicating a comparable age distribution across all intervention arms (*F* = 0.544, *p* = 0.655). The mean age was 32.55 ± 7.55 years in the VR + CBT group, 34.91 ± 8.89 years in the VR group, 36.33 ± 8.60 years in the CBT group, and 33.00 ± 7.62 years in the active control group (Table 1). Similarly, there were no significant between-group differences in sex distribution (*χ*^2^ = 0.495, *p* = 0.920). The distribution of earthquake-related life events also did not differ significantly across groups (*χ*^2^ = 16.157, *p* = 0.064), confirming baseline demographic and clinical comparability among the study groups (Table 2). In addition to demographic variables, no significant between-group differences were observed at baseline for primary or secondary outcome measures. Baseline RFT scores, VMS optical flow mean speed, optokinetic parameters, SVV, DSVV, DHI total and subscale scores, and PCL-5 scores did not differ significantly across intervention arms (all *p* > 0.05).

**Table 1:** Demographic characteristics of the study groups: age comparison

Variables	Groups	Mean ± SD	(Min—max)	*F*	*p*
Age	VR + CBT	32.55 ± 7.55	(22–43)	0.544	0.655
	VR	34.91 ± 8.89	(22–45)		
	CBT	36.33 ± 8.6	(19–47)		
	Active control	33 ± 7.62	(21–44)		

*Mean* mean, *SD* standard deviation, *F* one-way analysis of variance (ANOVA)

**Table 2:** Distribution of sex and earthquake-related life events across study groups

Variable	Group		Group				Total	*χ*^2^	*p*
			VR + CBT	VR	CBT	Active control			
Sex	1	*n*	7	7	8	7	29	0.495	0.920
		%	24.1%	24.1%	27.6%	24.1%	100.0%		
	2	*n*	4	5	4	6	19		
		%	22.2%	22.2%	22.2%	33.3%	100.0%		
Earthquake-related life events	1	*n*	5	1	4	4	14	16.157	0.064
		%	35.7%	7.1%	28.6%	28.6%	100.0%		
	2	*n*	5	8	2	4	19		
		%	26.3%	42.1%	10.5%	21.1%	100.0%		
	3	*n*	1	2	4	2	9		
		%	11.1%	22.2%	44.4%	22.2%	100.0%		
	4	*n*	0	0	2	3	5		
		%	0.0%	0.0%	40.0%	60.0%	100.0%		
Total		*n*	11	11	12	13	48		
		%	23.4%	23.4%	25.5%	27.7%	100.0%		

In participants with PEDS, changes in the physical subscale of the Dizziness-Related Disability Inventory (DHI) differed significantly across groups over time (Table 3, *p* < 0.05). The group × time interaction was statistically significant, with a partial eta-squared (*η*^2^) of 0.434, indicating that approximately 43.4% of the total variance in physical disability scores was explained by differential temporal effects across groups. This finding demonstrates that the magnitude and trajectory of change over time were not uniform among the intervention arms.

Changes in DHI physical, functional, emotional, and total scores differed significantly between groups over time (all *p* < 0.05). The group × time interaction demonstrated large effect sizes for all outcomes (physical *η*^2^ = 0.434; functional *η*^2^ = 0.762; emotional *η*^2^ = 0.697; total *η*^2^ = 0.762), indicating that a substantial proportion of variance in dizziness-related disability was attributable to differential temporal effects across intervention arms. These findings suggest that both the magnitude and trajectory of change varied meaningfully between treatment groups.

Within-group analyses revealed significant time-dependent improvements across intervention arms. In the VR + CBT group, DHI scores decreased significantly from pre- to post-intervention and from pre-intervention to follow-up across subscales (*p* < 0.05). The VR-only group demonstrated significant improvements across multiple time comparisons, particularly between pre–post and post–follow-up assessments (*p* < 0.05). The CBT group showed significant reductions over time, with improvements observed in at least one pairwise comparison for each outcome (*p* < 0.05). The active control group also exhibited significant time-related changes across pairwise comparisons (*p* < 0.05). Overall, although all groups showed temporal improvement, the magnitude and sustainability of change differed across treatment conditions, as reflected by the significant interaction effects.

**Fig. 3:**
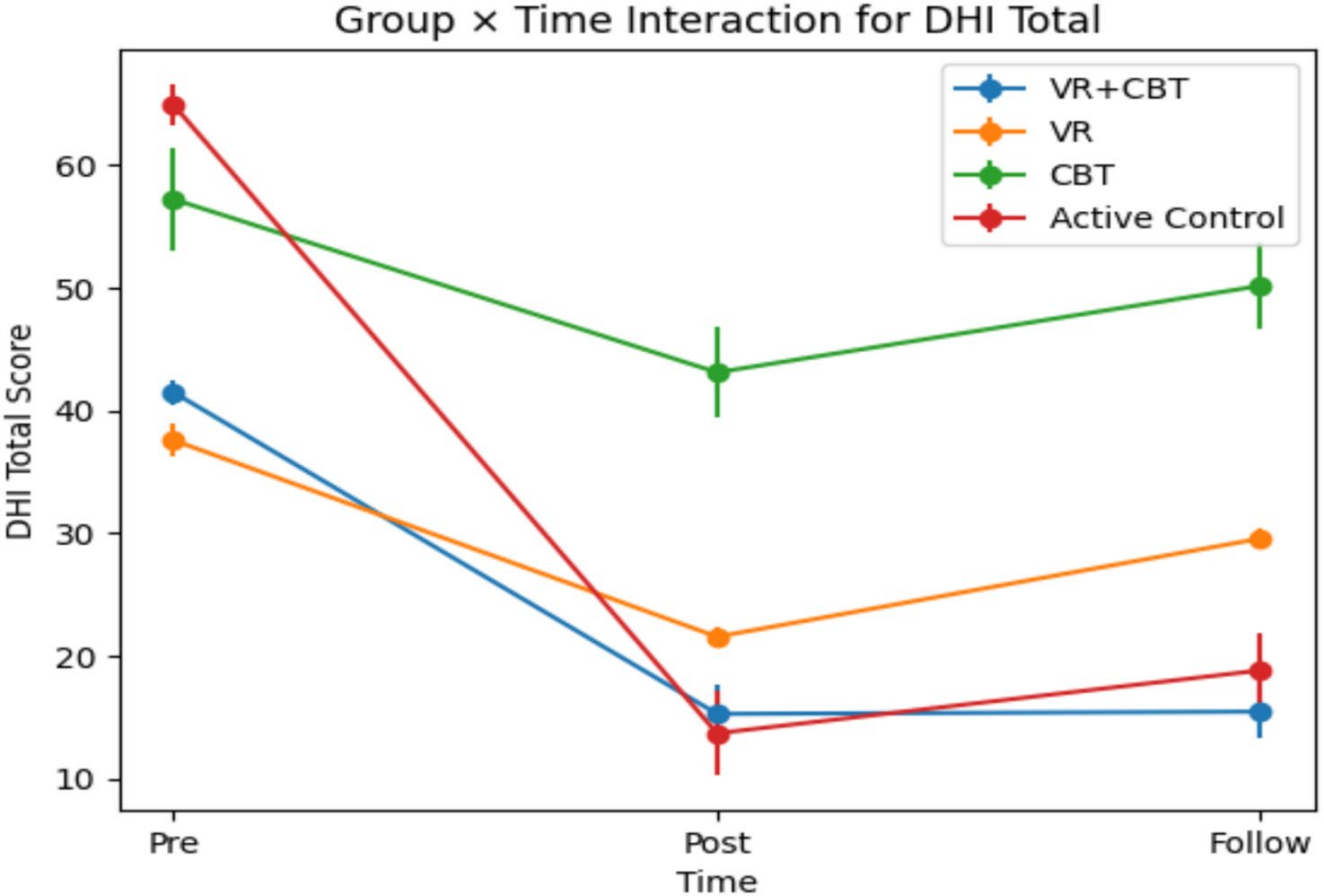
Group × time interaction for DHI-Total scores. Mean values at pre, post, and follow-up are shown for all groups. Error bars represent SD

Overall, although all groups showed temporal improvement, the magnitude and sustainability of change differed across treatment conditions, as reflected by the significant interaction effects. The overall trajectory of change in DHI-Total scores across groups is visually illustrated in Fig. 3, which depicts the group × time interaction pattern and highlights the differential improvement profiles among the intervention arms .

**Table 3:** Group × time effects on DHI subscales and total score

Outcome	Time	VR + CBT (mean ± SD)	VR	CBT	Active control	Group × time *F* *p* *η*^2^
DHI-physical	Pre	12.3 ± 2.3	11.0 ± 2.2	15.9 ± 5.3	18.5 ± 2.6	15.48 < 0.001 0.434
	Post	4.3 ± 2.1	5.5 ± 1.5	12.1 ± 5.0	4.7 ± 6.0	
	Follow	4.5 ± 2.0	8.2 ± 1.2	14.0 ± 4.2	6.1 ± 5.2	
DHI-functional	Pre	15.7 ± 1.8	13.6 ± 1.8	20.7 ± 4.6	24.1 ± 3.3	38.62 < 0.001 0.762
	Post	4.9 ± 3.8	13.6 ± 1.8	17.2 ± 5.1	4.5 ± 4.2	
	Follow	5.4 ± 3.4	10.6 ± 1.7	18.9 ± 4.0	6.4 ± 3.9	
DHI-emotional	Pre	13.5 ± 2.0	13.0 ± 2.1	20.6 ± 6.1	22.3 ± 4.0	26.43 < 0.001 0.697
	Post	6.1 ± 2.3	8.4 ± 2.8	13.8 ± 5.8	4.5 ± 3.2	
	Follow	6.4 ± 2.0	10.7 ± 1.9	17.2 ± 5.6	6.3 ± 2.9	
DHI-total	Pre	41.5 ± 3.5	37.6 ± 4.3	57.2 ± 14.5	64.9 ± 6.2	34.18 < 0.001 0.762
	Post	15.3 ± 7.7	21.6 ± 2.5	43.1 ± 12.8	13.7 ± 12.2	
	Follow	15.5 ± 7.2	29.6 ± 3.0	50.1 ± 12.0	18.8 ± 11.1	

*DHI* dizziness handicap inventory, *Mean* mean, *SD* standard deviation, *F* two-way mixed-model ANOVA, *F*^b^ repeated-measures ANOVA *t*-test, *η*^2^ eta-squared, *p < 0.05: statistically significant

In participants with PEDS, changes in Posttraumatic Stress Disorder Checklist for DSM-5 (PCL-5) scores differed significantly between groups over time (*p* < 0.05). The group × time interaction yielded a partial eta-squared (*η*^2^) of 0.198, indicating that approximately 19.8% of the variance in PTSD symptom severity was explained by differential temporal effects across the intervention groups. This result suggests that the pattern and magnitude of symptom change over time varied between groups.

Within-group analyses demonstrated significant time-dependent reductions in PCL-5 scores across all groups. In the VR + CBT group, significant improvements were observed from pre- to post-intervention and from pre-intervention to follow-up (*p* < 0.05). The VR-only and CBT-only groups both showed significant reductions across all pairwise comparisons (pre–post, pre–follow-up, and post–follow-up; *p* < 0.05). The active control group also exhibited significant decreases from pre- to post-intervention and from pre-intervention to follow-up (*p* < 0.05) (Table 4).

**Table 4:** Group × time effects on post-traumatic stress symptoms (PCL-5 total score)

Variable	VR + CBT		VR		CBT		Active control		Between-group comparison	Independent between-group comparison	
	Mean ± SD		Mean ± SD		Mean ± SD		Mean ± SD			*F*^a^	*p*^a^
Pre-PCL-5	20.55 ± 6.06		17.91 ± 5.92		20.67 ± 6.56		19.31 ± 6.82		*F* = 3.545	*F* = 0.459	*p* = 0.712
Post-PCL-5	7.91 ± 3.88		4.27 ± 2.69		5.33 ± 3.45		3.62 ± 3.45		*p* = 0.003	*F* = 3.55	*p* = 0.022
Follow-PCL-5	9.64 ± 4.15		11.09 ± 3.38		13 ± 3.86		5.18 ± 3.47		*η*^2^ = 0.198	*F* = 10.032	*p* = 0
Within-group comparison (*F*^b^)	*F* = 26.576	*p* = 0	*F* = 31.799	*p* = 0	*F* = 43.861	*p* = 0	*F* = 55.368	*p* = 0			
Pre-post	*p* = 0		*p* = 0		*p* = 0		*p* = 0				
Pre-follow	*p* = 0		*p* = 0		*p* = 0		*p* = 0				
Post-follow	*p* = 0.143		*p* = 0		*p* = 0		*p* = 0.151				

*PCL-5* post-traumatic stress disorder checklist, *Mean* mean, *SD* standard deviation, *F* two-way mixed-model ANOVA, *F*^b ^repeated-measures ANOVA *t*-test, *η*^2^ eta-squared, *p < 0.05: statistically significant

In participants with PEDS, neither static nor dynamic subjective visual vertical measures showed significant differences between groups across time (*p* > 0.05). Similarly, within-group analyses revealed no significant time-related changes in SVV or DSVV values in any of the intervention arms, including the VR + CBT, VR-only, CBT-only, and active control groups (*p* > 0.05). These findings indicate that visuo-vestibular verticality perception, assessed under both static and dynamic conditions, remained stable over the intervention and follow-up period (Table 5).

**Table 5:** Group × time effects on static and dynamic subjective visual vertical (SVV, DSVV)

Outcome	Time	VR + CBT (mean ± SD)	VR	CBT	Active control	Group × time *F* *p* *η*^2^
SVV (°)	Pre	1.75 ± 2.03	0.38 ± 2.22	1.57 ± 1.95	1.2 ± 3.36	0.55 0.660 0.04
	Post	0.36 ± 0.7	0.37 ± 0.98	0.99 ± 1.6	1.49 ± 3.13	
	Follow	0.5 ± 0.57	0.37 ± 0.95	1.16 ± 1.22	1.35 ± 1.71	
DSVV (°)	Pre	−3.13 ± 7.72	−4.01 ± 6.39	−1.31 ± 4.91	−5.03 ± 8.19	0.10 0.64 0.01
	Post	−1.14 ± 2.18	−2.93 ± 2.9	−1.06 ± 4.44	−3.08 ± 7.68	
	Follow	−1.34 ± 1.92	−3.25 ± 2.4	−1.13 ± 3.16	−4.05 ± 4.13	

*SVV* subjective visual vertical, *DSVV* dynamic subjective visual vertical, *Mean* mean, *SD* standard deviation, *F* two-way mixed-model ANOVA, *F*^b^ repeated-measures ANOVA *t*-test, *η*^2^ eta-squared, *p < 0.05: statistically significant

In participants with PEDS, no significant between-group differences were observed in mean Rod-and-Frame Test (RFT) scores across time (*p* > 0.05). However, within-group analyses revealed significant time-dependent changes in several intervention arms. In the VR + CBT group, RFT scores showed a significant change over time, with a significant difference between baseline and follow-up assessments (*p* < 0.05). The VR-only group demonstrated significant changes across all time comparisons, including baseline–post-treatment, baseline–follow-up, and post-treatment–follow-up (*p* < 0.05). In the CBT group, significant improvements were observed between baseline and post-treatment, as well as between baseline and follow-up (*p* < 0.05). In contrast, no significant temporal changes were detected in the active control group (*p* > 0.05) (see Table 6).

**Table 6:** Group × time changes in rod-and-frame test (RFT) scores

Variable	VR + CBT		VR		CBT		Active control		Between-group comparison	Independent between-group comparison	
	Mean ± SD		Mean ± SD		Mean ± SD		Mean ± SD			*F*^a^	*p*^a^
Pre-RFT mean	6.54 ± 6.25		10.43 ± 5.96		11.66 ± 4.2		9.63 ± 5.35		*F* = 1.545	*F* = 1.807	*p* = 0.16
Post-RFT mean	2.58 ± 2.54		5.36 ± 2.47		7.09 ± 3.74		9.08 ± 4.34		*p* = 0.173	*F* = 7.649	*p* = 0
Follow-RFT mean	2.97 ± 2.39		6.88 ± 2.09		8.46 ± 3.41		9.35 ± 3.95		*η*^2^ = 0.097	*F* = 9.506	*p* = 0
Within-group comparison (*F*^b^)	*F* = 6.456	*p* = 0.004	*F* = 3.936	*p* = 0.027	*F* = 3.485	*p* = 0.04	*F* = 0.16	*p* = 0.853			
Pre-post	*p* = 0.097		*p* = 0.021		*p* = 0.032		*p* = 1				
Pre-follow	*p* = 0.026		*p* = 0.026		*p* = 0.04		*p* = 1				
Post-follow	*p* = 1		*p* = 0.039		*p* = 0.057		*p* = 1				

*RFT* rod-and-frame test, *Mean* mean, *SD* standard deviation, *F* two-way mixed-model ANOVA, *F*^b^ repeated-measures ANOVA *t*-test, *η*^2^: eta-squared, *p < 0.05: statistically significant

**Fig. 4:**
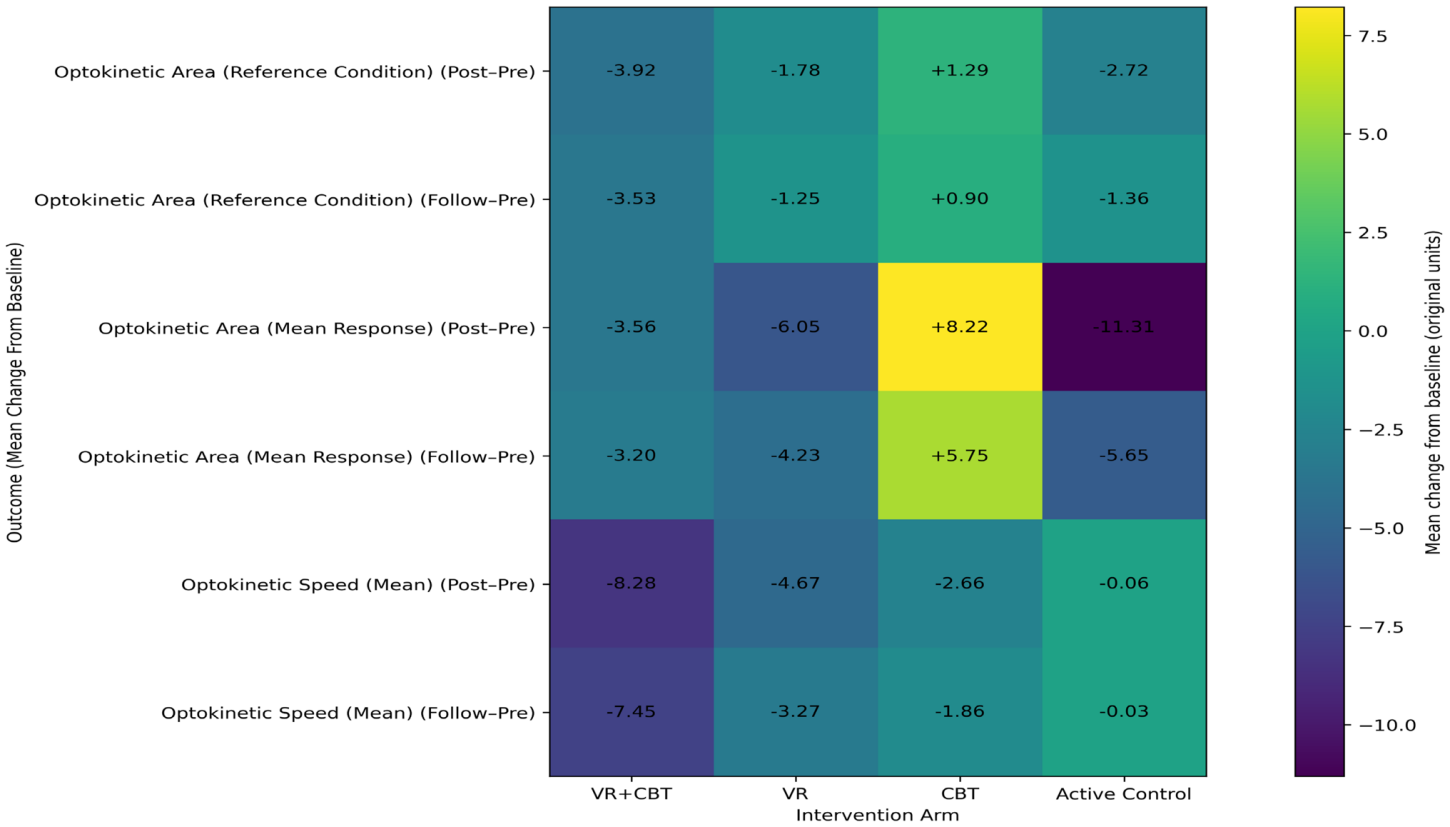
Heatmap of mean changes in optokinetic-based visual motion sensitivity outcomes by intervention arm

**Fig. 5:**
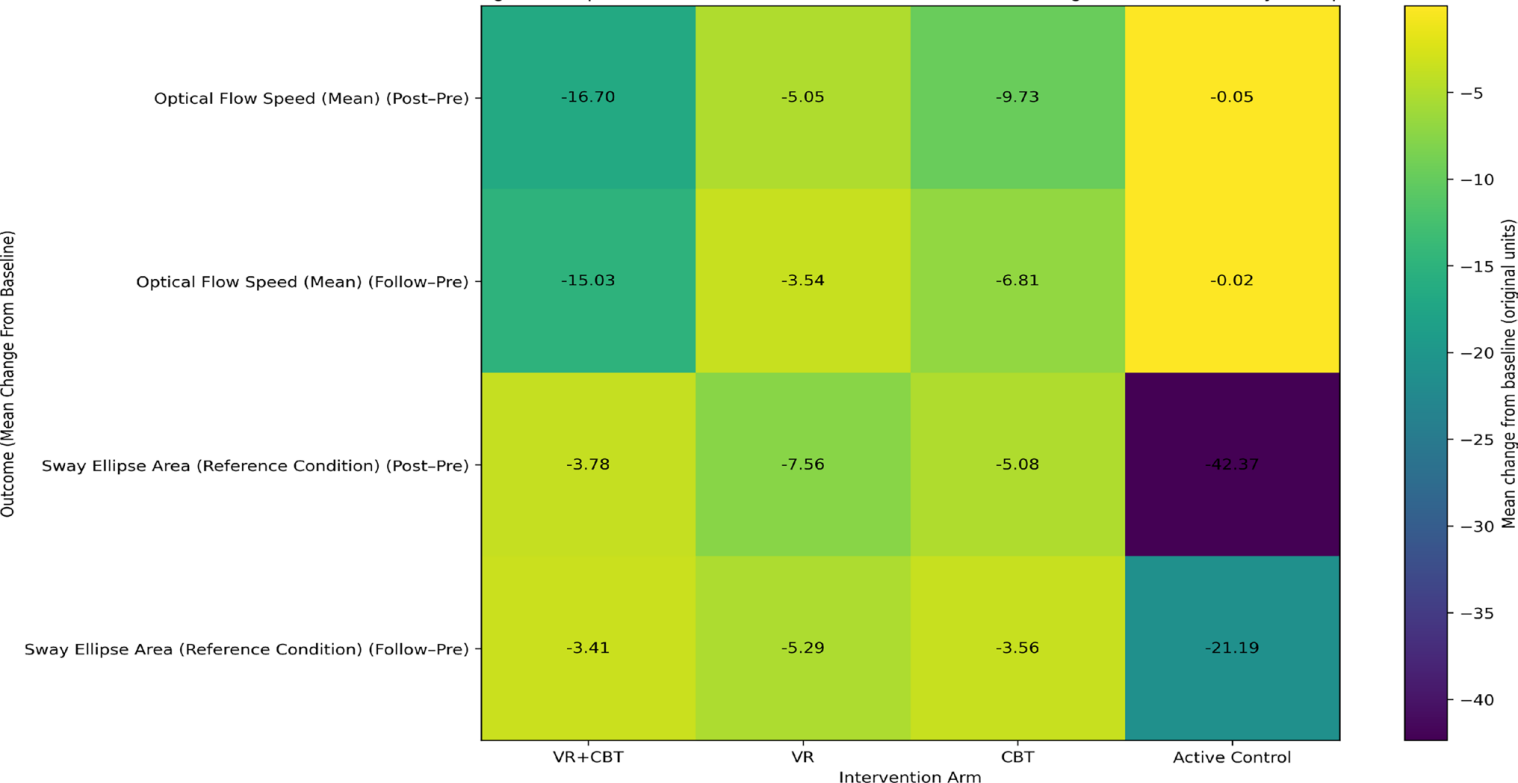
Heatmap of mean changes in optical flow–related postural instability measures by intervention arm

In patients with PEDS, significant group × time interactions were observed for VMS optokinetic area (reference and mean values), optical flow mean speed, and sway ellipse area reference (all *p* < 0.05; *η*^2^ ranging from 0.191 to 0.294), indicating that temporal changes differed across intervention arms and that a moderate proportion of variance was explained by the interaction effects. While certain within-group time-dependent improvements were detected—particularly in the VR + CBT group for selected optokinetic parameters and across multiple arms for optical flow speed—these patterns were not uniform across outcomes, and some parameters (e.g., optokinetic mean speed and sway ellipse area reference) did not demonstrate consistent within-group shifts despite significant interaction effects. No significant between-group interaction was found for optokinetic mean speed. Detailed statistical results are presented in Tables 7, and the direction and magnitude of mean changes from baseline are visually summarized in the heatmaps provided in Figs. 4 and 5.

**Table 7:** Group × time effects on visual motion sensitivity (VMS) outcomes in PEDS

Outcome	Time	VR + CBT (mean ± SD)	VR	CBT	Active control	Group × time *F* *p* *η*^2^
Optokinetic area (ref)	Pre	6.03 ± 4.01	3.25 ± 3.05	2.56 ± 1.93	5.56 ± 3.83	4.13 0.012 0.191
	Post	2.11 ± 1.65	1.47 ± 1.32	3.85 ± 2.9	2.84 ± 2.47	
	Follow	2.50 ± 1.59	2.00 ± 1.13	3.46 ± 2.03	4.20 ± 1.69	
Optokinetic area (mean)	Pre	5.96 ± 5.59	10.83 ± 13.66	3.71 ± 3.06	20.80 ± 11.61	8.16 < 0.001 0.294
	Post	2.40 ± 1.62	4.78 ± 5.84	11.93 ± 8.63	9.49 ± 9.88	
	Follow	2.76 ± 1.81	6.60 ± 4.84	9.46 ± 6.03	15.15 ± 8.46	
Optokinetic speed (mean)	Pre	16.59 ± 11.26	13.30 ± 7.8	9.51 ± 5.39	13.14 ± 6.43	4.54 0.008 0.157
	Post	8.31 ± 4.97	8.63 ± 3.14	6.85 ± 2.95	13.08 ± 4.93	
	Follow	9.14 ± 5.49	10.03 ± 3.49	7.65 ± 2.42	13.11 ± 3.63	
Optical flow speed (mean)	Pre	21.5 ± 7.23	16.51 ± 14.85	18.98 ± 9.97	29.82 ± 14.94	20.71 < 0.001 0.225
	Post	4.80 ± 3.45	11.46 ± 10.58	9.25 ± 4.24	29.77 ± 9.94	
	Follow	6.47 ± 2.93	12.97 ± 11.26	12.17 ± 2.74	29.80 ± 9.39	
Sway ellipse area (ref)	Pre	6.51 ± 5.75	11.07 ± 28.81	9.31 ± 23.85	52.39 ± 38.17	14.63 < 0.001 0.244
	Post	2.73 ± 2.49	3.51 ± 7.13	4.23 ± 10.72	10.02 ± 7.82	
	Follow	3.10 ± 2.55	5.78 ± 10.5	5.75 ± 9.65	31.20 ± 17.76	

*VMS* visual motion sensitivity, *Mean* mean value, *SD* standard deviation, *F* two-way mixed-model ANOVA, *F*^b^ repeated-measures ANOVA *t*-test, *η*^2^ eta squared, *p < 0.05 indicates statistical significance

This heatmap illustrates mean changes from baseline (Post–Pre and Follow-up–Pre) for optokinetic-based visual motion sensitivity parameters, including Optokinetic Area (Reference Condition), Optokinetic Area (Mean Response), and Optokinetic Speed (Mean), across the VR + CBT, VR, CBT, and active control groups. Negative values indicate reductions from baseline, reflecting decreased visually induced postural disturbance. The heatmap is intended to visualize the direction and magnitude of change across intervention arms; values represent mean change only, and within-group variability is not displayed. Statistical significance of group × time effects is reported in Table 7.

This heatmap presents mean baseline-referenced changes (Post–Pre and Follow-up–Pre) for Optical Flow Speed (Mean) and Sway Ellipse Area (Reference Condition) across intervention arms. The visualization highlights intervention-specific patterns in visually induced postural instability and sensory reweighting over time. Cell values correspond to mean changes in the original measurement units; variability within groups is not shown. Inferential statistics for these outcomes are provided in Table 7.

## Discussion

This longitudinal four-arm study examined whether treatment in post-earthquake dizziness syndrome modulates objective visuo-vestibular integration across complementary domains including visual verticality measured by SVV and DSVV, visual frame dependence assessed with the rod and frame test, and visual motion sensitivity, from baseline to post-intervention and three-month follow-up. Beyond symptomatic improvement, we tested whether interventions targeting both sensory processing and cognitive-emotional drivers yield durable changes in visuo-vestibular function. Although PEDS is increasingly recognized as clinically meaningful and frequently accompanied by psychological burden, objective and mechanism-oriented longitudinal comparisons of virtual reality-based rehabilitation, cognitive behavioral therapy, and an integrated VR plus CBT approach remain scarce. Our findings address this gap by demonstrating a domain-specific response profile characterized by improvement in symptom burden and selective modulation of frame dependence and visual motion responsivity, alongside relative stability of verticality measures. We hypothesized treatment-related changes in objective outcomes, with superior durability for the integrated VR + CBT approach. This hypothesis was partially supported in a domain-specific manner: verticality measures (SVV/DSVV) remained stable, whereas visual context dependence (RFT) and several VMS metrics showed treatment-related modulation, most consistently in the integrated arm under visually provocative conditions.

Across treatment arms, dizziness-related handicap demonstrated clinically meaningful improvement over time, supporting the clinical relevance of PEDS beyond transient discomfort and highlighting its impact across physical, functional, and emotional domains [6]. While Cengiz et al. (2024) demonstrated associations among dizziness severity, dizziness-related handicap, and post-traumatic stress symptoms following the 2023 Türkiye earthquakes, their screening design could not evaluate treatment responsiveness or temporal change. In contrast, our longitudinal trajectories indicate that handicap in PEDS is modifiable through targeted interventions, aligning with a multidimensional management approach rather than conceptualizing PEDS as self-limiting or purely stress-related.

Post-traumatic stress symptoms were also prominent and improved over time, consistent with evidence linking post-earthquake dizziness to trauma-related psychopathology. Specifically, dizziness severity showed a significant positive relationship with PTSD symptom burden in earthquake-exposed cohorts [6], and PEDS has been described as closely intertwined with emotional dysregulation, anxiety, and social stressors [3]. Adolescents with PEDS likewise exhibit higher PTSD scores than those without dizziness [32]. Within this framework, reductions in PCL-5 may reflect attenuation of a sustaining factor that can amplify dizziness-related complaints and hinder engagement with rehabilitation.

Notably, the magnitude of improvement observed in the active control group warrants careful interpretation. In post-disaster contexts, dizziness and related distress are closely linked to perceived threat, uncertainty, and a diminished sense of bodily safety. Disaster mental health frameworks emphasize that restoration of a sense of safety through regular clinical contact, structured follow-up, and predictable healthcare engagement represents a core mechanism supporting symptom reduction, even in the absence of active therapeutic intervention [33]. In this respect, ongoing monitoring and sustained contact with healthcare providers may enhance reassurance and perceived health security, thereby attenuating both symptom severity and psychological burden. From a vestibular and perceptual standpoint, improvement in the active control condition is therefore more plausibly explained by modulation of threat appraisal and bodily vigilance rather than by direct sensorimotor recalibration. Contemporary models of chronic dizziness highlight that heightened symptom monitoring, illness-focused attention, and maladaptive interpretations of bodily sensations play a central role in symptom persistence and recovery trajectories [34]. Accordingly, while clinic-based treatment exposure remains essential for rehabilitation, repeated engagement with symptom-focused assessments may transiently reinforce illness salience in some individuals and subtly shape emotional recovery independently of objective visuo-vestibular adaptation.

Taken together, these observations indicate that symptom improvement in PEDS can emerge through multiple pathways, including reassurance and restoration of perceived safety, and should not be interpreted as evidence against treatment-specific mechanisms demonstrated in objective visuo-vestibular outcomes.

Objective outcomes showed heterogeneous responsiveness across domains. Measures of verticality, SVV and DSVV, demonstrated no meaningful group or time-related change. In contrast, the rod and frame test and visual motion sensitivity exhibited treatment-related effects in specific contexts. This dissociation indicates that PEDS is less likely to reflect a global distortion of gravitational verticality and more likely to involve context-dependent sensory reweighting under visually ambiguous frames and motion-rich stimulation. Experimental work also shows that congruent visuo-vestibular signals can reshape higher-order perceptual binding, supporting the view that context-dependent multisensory integration—rather than baseline verticality estimation—may be the more treatment-responsive bottleneck in visually loaded dizziness phenotypes [35]. This pattern is consistent with sensory conflict models of post-earthquake dizziness, which emphasize visually driven vulnerability rather than uniform otolithic bias [2, 10], and aligns with observations that prolonged exposure to tilted environments can sustain dizziness and spatial disorientation despite largely normal peripheral vestibular findings [11].

Recent comprehensive reviews, particularly by Miwa and colleagues, have synthesized current evidence to conceptualize PEDS as a multifactorial condition arising from the interaction of vestibular dysfunction, autonomic dysregulation, sensory conflict within the velocity storage system, psychological stress, and persistent environmental instability rather than a single peripheral lesion. Within this framework, some studies emphasize peripheral vestibular and otolithic involvement, reporting deterioration in cVEMP, postural sway, and vHIT findings following major earthquakes, especially after the 2016 Kumamoto events, and linking seismic forces to otoconia displacement and BPPV-like presentations[2, 36, 37]. However, Miwa et al. also underline that repeated low-frequency ground motion and aftershocks can preferentially sensitize central sensory integration mechanisms, amplifying visually induced dizziness through sensory conflict and velocity storage system dysfunction, even when baseline graviceptive processing remains relatively intact [2, 38, 39].

Interpreted within contemporary models, our findings align more closely with sensory conflict and central integration mechanisms of PEDS than with a primary otolithic dysfunction hypothesis. The absence of meaningful change in SVV and DSVV argues against a dominant impairment of gravitational verticality perception, as would be expected in overt utricular pathology [2]. In contrast, treatment-related modulation in rod-and-frame performance and visual motion sensitivity supports context-dependent visual reweighting under visually ambiguous or motion-rich conditions, consistent with velocity storage–based models of earthquake-induced dizziness [2, 39, 40]. Miwa and colleagues further note that low-frequency sway, typical of tall structures, can prolong recovery through repeated aftershocks by amplifying sensory mismatch [10, 41]. The concurrent improvement in psychological outcomes and the response observed in the active control group underscore the modulatory role of autonomic arousal and stress-related amplification rather than primary vestibular injury [3, 4]. Collectively, these findings suggest that in our cohort, PEDS predominantly reflects maladaptive multisensory integration under persistent environmental instability, rather than uniform peripheral vestibular or otolithic damage.

Methodologically, these findings support multi-domain objective profiling. Bozduman Çelebi & Akdağ (2024 [32] noted the limitation of lacking objective vestibular/balance measures in earthquake-exposed adolescents; our data extend the field by showing that objective indices can remain stable in some domains (SVV/DSVV) while changing in others (RFT/VMS), emphasizing the importance of domain-specific interpretation.

Our longitudinal pattern suggests that integrated VR + CBT produces the most consistent improvements in visuo-vestibular integration markers—particularly under visually provocative VMS conditions—and shows within-group gains in frame dependence (RFT). In contrast, the active control trajectory is a comparatively meaningful improvement despite active control and sometimes atypical. Notably, the active control condition also showed clinically meaningful improvements in symptom and psychological measures, highlighting the contribution of non-specific therapeutic effects in post-disaster cohorts. Conceptually, VR-based vestibular rehabilitation offers graded multisensory exposure and recalibration under controlled visual motion, while CBT targets threat appraisal, hypervigilance, avoidance, and maladaptive symptom interpretations that can perpetuate dizziness [10, 42]. Integrated VR + CBT did not induce uniform changes across all visuo-vestibular domains; however, it yielded the most coherent and durable improvements in context-dependent measures (RFT and visually provocative VMS), supporting the second hypothesis that combined sensory and cognitive–emotional targeting confers an advantage over single-modality or non-specific interventions.

Miwa et al. (2025)[10] highlight ANS involvement and central sensory conflict as contributors to persistent motion sensations; from this angle, the stronger VMS improvements with VR + CBT are consistent with the idea that reducing arousal-driven amplification and improving tolerance to visually induced mismatch may facilitate more durable adaptation. Importantly, the persistence of SVV/DSVV stability even in the integrated arm reinforces that treatment effects are not uniform across all visuo-vestibular measures; instead, they preferentially appear in visually loaded, context-sensitive processing.

Population and clinical reports indicate that PEDS is common and clinically meaningful, frequently co-occurring with psychological burden and sometimes with limited peripheral vestibular signals [10, 37]. Experimental work also suggests that earthquake/aftershock exposure can be associated with measurable equilibrium dysfunction and that anxiety correlates with postural sway parameters [4]. Our study extends these lines by adding a longitudinal, multi-arm intervention framework and targeting objective markers that index visual frame dependence and visual-motion sensitivity—components that naturally sit within sensory conflict accounts and are directly addressable by VR-based exposure paradigms.

Clinically, these results argue for assessment and follow-up that goes beyond symptom inventories. When SVV/DSVV remain unchanged but VMS and RFT shift, clinicians obtain a more precise map of what is changing: not necessarily baseline verticality estimation, but vulnerability under visual motion and misleading frames. In post-disaster settings where repeated aftershocks, unstable environments, and persistent threat cues may sustain symptoms, objective profiling may help tailor treatment selection—prioritizing integrated approaches when visual-motion provocation and frame dependence are prominent [11].

In the present study, the primary outcome was predefined as the group × time interaction for VMS optical flow mean speed, selected as the most direct objective marker of visually induced sensory conflict modulation. Although the sample size was constrained by post-disaster recruitment conditions, the observed interaction effects with moderate-to-large effect sizes support the robustness of the main mechanistic finding. Nevertheless, replication in larger cohorts is warranted to further strengthen statistical precision.

Although the study was longitudinal and comparative, pragmatic post-disaster constraints limited strict randomization, and residual confounding cannot be fully excluded. A parallel healthy control arm was not included; however, device-specific normative reference data obtained during the standardization phase were used to contextualize baseline deviations. Assessor blinding was not feasible, yet the use of objective, instrument-based outcome measures reduces the likelihood of substantial measurement bias. While our battery primarily targets visuo-vestibular integration, future studies may integrate broader vestibular profiling and autonomic markers to refine mechanistic subtypes and improve prediction of persistence or relapse.

## Conclusion

In summary, our findings support a multi-domain view of PEDS in which clinical improvement is accompanied by selective modulation of visuo-vestibular integration. Treatment effects were most evident in measures capturing visual context dependence (RFT) and visual-motion responsivity (VMS), while SVV/DSVV remained comparatively stable, suggesting domain-specific responsiveness. The integrated VR + CBT approach yielded the most consistent and durable objective improvements, lending support to multidisciplinary models in which sensory conflict and cognitive–emotional factors jointly shape recovery after major earthquakes.

## Supplementary Information

Below is the link to the electronic supplementary material.

Supplementary file1 (PDF 1477 KB)

Supplementary file2 (PDF 997 KB)

Supplementary file3 (DOCX 296 KB)

## Data Availability

The datasets generated and/or analyzed during the current study are not publicly available due to ethical restrictions and the sensitive nature of post-disaster clinical data, but are available from the corresponding author on reasonable request.
