# Supplementary material for: Treatment-related modulation of visuo-vestibular integration in post-earthquake dizziness syndrome: a longitudinal virtual reality–based study
Source: J Neurol. 2026 Mar 24;273(4):230. doi: 10.1007/s00415-026-13767-4 (PMC13013104; doi:10.1007/s00415-026-13767-4)
Supplement: Supplementary file 1 — Supplementary file1 (PDF 1477 KB) [file 415_2026_13767_MOESM1_ESM.pdf]

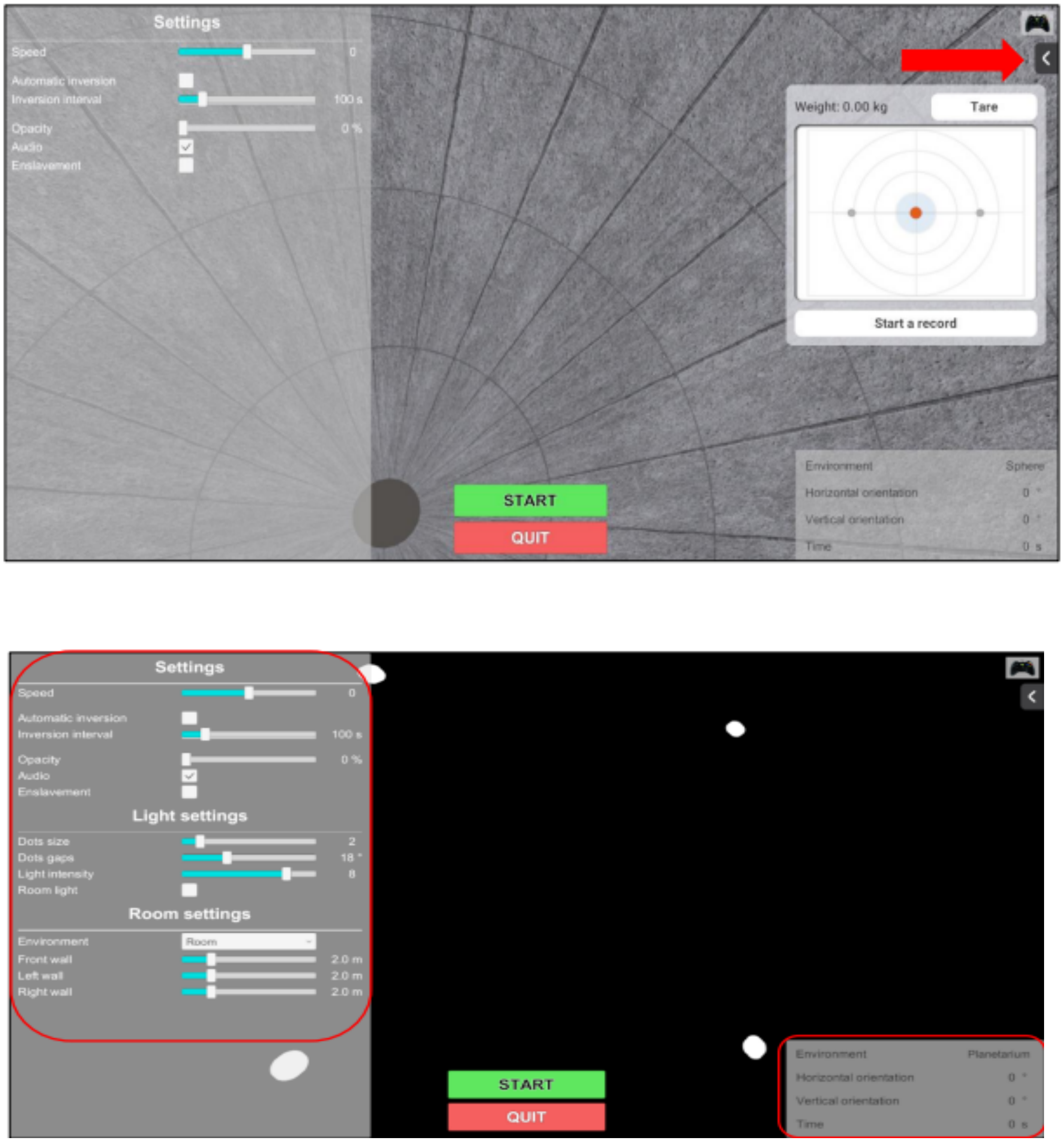

Figure 1. Optokinetic visual motion sensitivity scenarios used to assess visuo-vestibular sensory conflict under full-field visual motion.

Check this option to make the arrows visible and help the patient visualize the direction of movement of the bar (right or left).

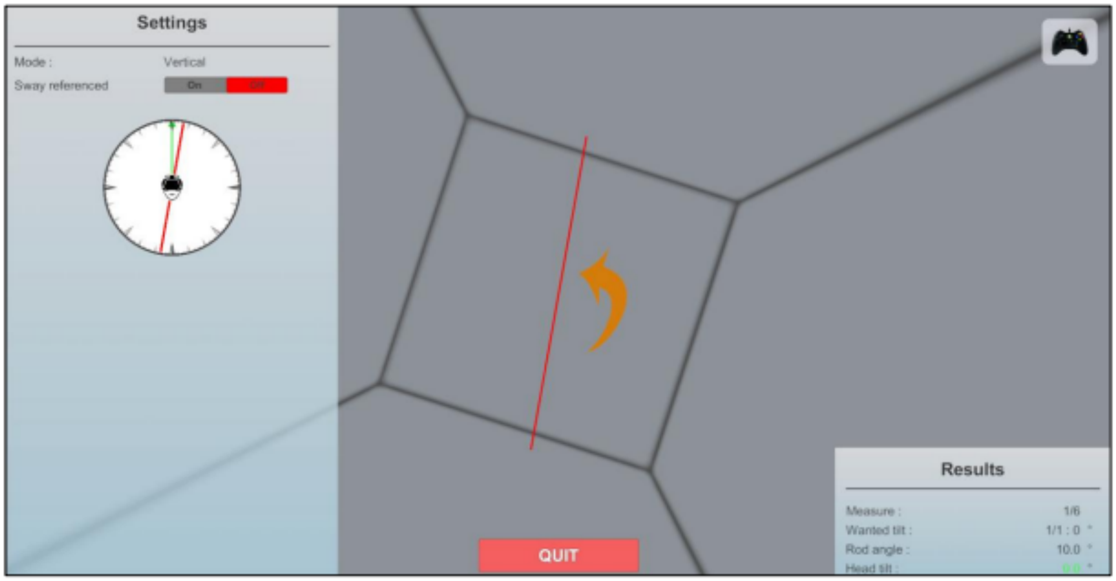

Figure 2. RFT scenario.

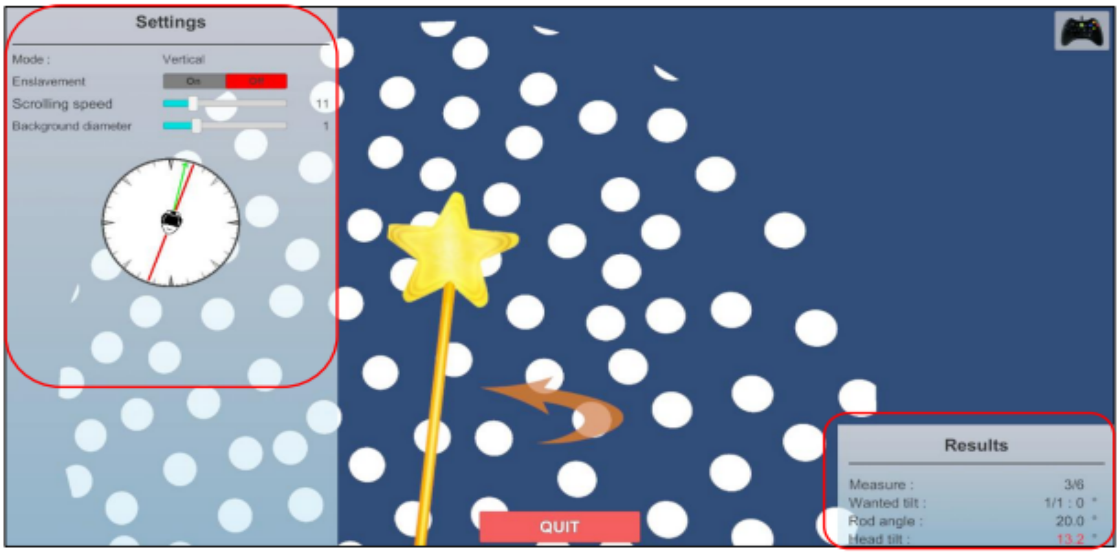

Figure 3. Dynamic SVV scenario.

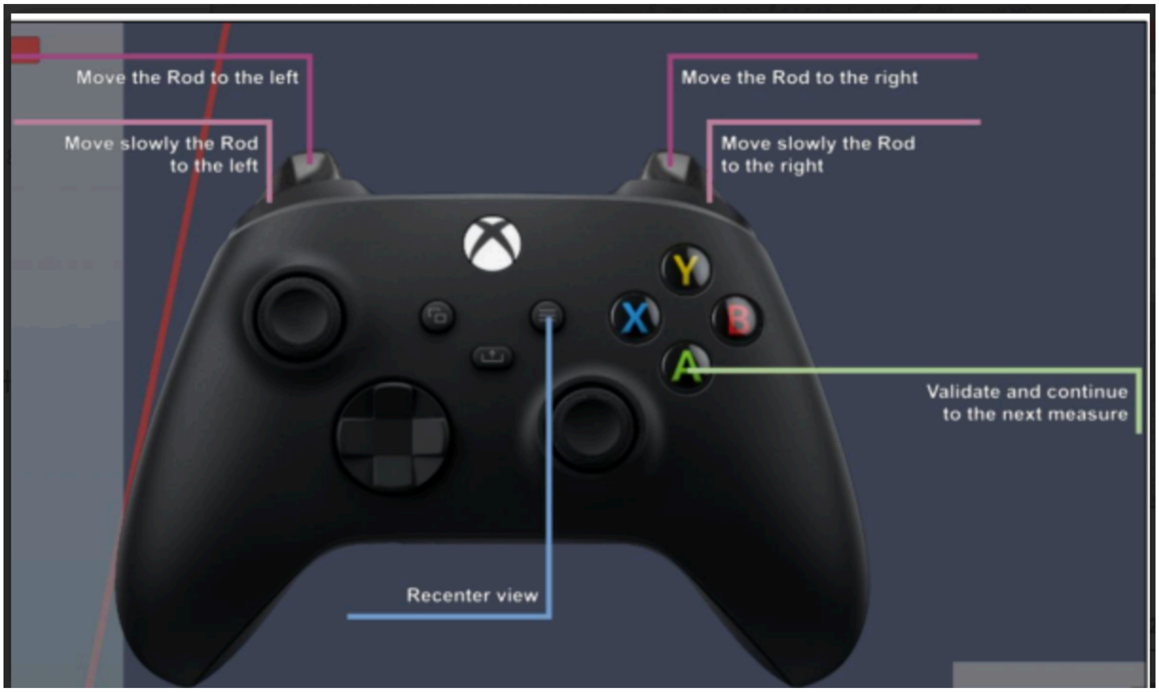

Figure 4. Xbox game controller interface used by participants to adjust stimulus orientation and confirm responses during visuo-vestibular test execution.

1. BULGULAR

Çalışmaya toplam 47 katılımcı dahil edilmiştir. Gruplar arasında yaş açısından anlamlı fark saptanmamıştır (F=0,544; p=0,655). Tüm gruplarda yaş dağılımı benzer olup ortalama yaş VR+BDT grubunda 32,55±7,55; VR grubunda 34,91±8,89; BDT grubunda 36,33±8,60 ve aktif kontrol grubunda 33,00±7,62'dir (Bkz. **Tablo 1**).

Cinsiyet dağılımı açısından gruplar arasında anlamlı bir fark bulunmamıştır (χ^2=0,495; p=0,920). Aynı şekilde, katılımcıların yaşadıkları olayların gruplara göre dağılımı da istatistiksel olarak farklılık göstermemiştir (χ^2=16,157; p=0,064) (Bkz. **Tablo 2**).

Tablo 1: Yaşa göre grupların karşılaştırılması.

Değişkenler	Gruplar	Ort ± ss	(Min - Max)	F	p
Yaş	VR+BDT	32,55 ± 7,55	(22-43)	0,544	0,655
	VR	34,91 ± 8,89	(22-45)		
	BDT	36,33 ± 8,6	(19-47)		
	Aktif Kontrol	33 ± 7,62	(21-44)		

Ort; ortalama, ss; standart sapma, F; Bağımsız ANOVA testi

Bu projeye dahil edilen DSBS tanılı katılımcılarda yaşa göre gruplar arasında istatistiksel farklılık bulunmadı (p>0,05).

Tablo 2: Cinsiyet ve yaşanılan olayların gruplara göre karşılaştırılması.

Değişken	Grup		Group				Total	χ^2	p
			VR+BDT	VR	BDT	Aktif Kontrol			
Cinsiyet	1	n	7	7	8	7	29	0,495	0,920
		%	24,1%	24,1%	27,6%	24,1%	100,0%		
	2	n	4	4	4	6	18		
		%	22,2%	22,2%	22,2%	33,3%	100,0%		
Yaşam_Olayları_kontrol	1	n	5	1	4	4	14	16,157	0,064
		%	35,7%	7,1%	28,6%	28,6%	100,0%		
	2	n	5	8	2	4	19		
		%	26,3%	42,1%	10,5%	21,1%	100,0%		
	3	n	1	2	4	2	9		
		%	11,1%	22,2%	44,4%	22,2%	100,0%		
	4	n	0	0	2	3	5		
		%	0,0%	0,0%	40,0%	60,0%	100,0%		
Total		n	11	11	12	13	47		
		%	23,4%	23,4%	25,5%	27,7%	100,0%		

Bu projeye dahil edilen DSBS tanılı katılımcılarda cinsiyet ve yaşanılan olaya göre gruplar arasında istatistiksel farklılık saptanmadı (χ^2=16,157; p=0,064).

Tablo 3. Grupların fHIT Lateral Kanal Skorlarının Zaman İçinde Değişiminin Analizi (Pre–Post–Follow-Up).

Değişken	VR+BDT		VR		BDT		Aktif Kontrol		Gruplar arası Karşılaştırma	Gruplararası Bağımsız Karşılaştırma	
	Ort ± ss		Ort ± ss		Ort ± ss		Ort ± ss			F[a]	p[a]
**Pre- fhit Lateraller (%)**	73 ± 6,57		81,18 ± 17,57		73,58 ± 17,93		92,73 ± 10,75		F =10,276	F =5,339	p =0,003
**Post- fhit Lateraller (%)**	95,94 ± 3,62		93,07 ± 5,96		89,1 ± 8,85		78,77 ± 16,09		p =0	F =6,662	p =0,001
**Follow- fhit Lateraller (%)**	93,65 ± 3,23		89,5 ± 4,81		84,44 ± 7,52		85,75 ± 10,59		η =0,418	F =3,686	p =0,019
**Grupiçi Karşılaştırma (F[b])**	F =59,111	p =0	F =2,815	p =0,071	F =5,234	p =0,009	F =12,972	p =0			
Pre-Post	p =0		p =0,098		p =0,013		p =0,022				
Pre-Follow	p =0		p =0,074		p =0,008		p =0,119				
Post-Follow	p =0,722		p =0,214		p =0,046		p =0,001				

Ort: Ortalama, ss: Standart sapma, F: İki yönlü karma model ANOVA, Fb: Tekrarlayan ölçümlerde ANOVA (bağımlı gruplar analizi), η²: Eta kare etki büyüklüğü. Pre = uygulama öncesi, Post = uygulama sonrası, Follow-up = izlem ölçümü. *p < 0,05: İstatistiksel olarak anlamlı fark vardır.

Çalışmaya alınan katılımcılarda fHIT lateral kanal ölçümlerinin değişimlerinde gruplar arasında istatistiksel olarak anlamlı farklılık bulundu (p<0,05). Gruplar arası zamana göre değişimde elde edilen kısmi eta kare (η²) değeri 0.418, etkileşimin toplam varyansın yaklaşık % 41.8'ini açıkladığını göstermektedir. Grup zaman etkileşimi zamanın her grupta etkisinin aynı olmadığını göstermektedir.

Grup içi zamana göre karşılaştırmalarda;

- VR+BDT grubunda ölçümlerin zamana göre uygulama öncesi ve sonrası değişimi istatistiksel olarak anlamlı bulundu (p<0.05).
  - Pre ile post, pre ile follow arasındaki değişim istatistiksel olarak anlamlı bulundu (p<0.05).

- VR grubunda ölçümlerin zamana göre uygulama öncesi ve sonrası değişimi istatistiksel olarak anlamlı bulunmadı (p>0.05).
- BDT grubunda ölçümlerin zamana göre uygulama öncesi ve sonrası değişimi istatistiksel olarak anlamlı bulundu (p<0.05).
  - Pre ile post, pre ile follow, post ile follow arasındaki değişim istatistiksel olarak anlamlı bulundu (p<0.05).
- Aktif kontrol grubunda ölçümlerin zamana göre uygulama öncesi ve sonrası değişimi istatistiksel olarak anlamlı bulundu (p<0.05).
  - Pre ile post, post ile follow arasındaki değişim istatistiksel olarak anlamlı bulundu (p<0.05) (Bkz. Tablo 3).

Tablo 4. Grupların fHIT Anterior Kanal Skorlarının Zaman İçinde Değişiminin Analizi (Pre–Post–Follow-Up).

**Değişken**	**VR+BDT**		**VR**		**BDT**		**Aktif Kontrol**		**Gruplar arası Karşılaştırma**	**Gruplararası Bağımsız Karşılaştırma**	
	**Ort ± ss**		**Ort ± ss**		**Ort ± ss**		**Ort ± ss**			**F^a**	**p^a**
**Pre-fhit Anteriorlar (%)**	84,45 ± 9,37		87,79 ± 14,1		79,28 ± 22,25		94,75 ± 9,03		F =2,407	F =2,423	p =0,079
**Post-fhit Anteriorlar (%)**	97,55 ± 5,52		94,36 ± 6,87		91,36 ± 13,94		88,44 ± 16,84		p =0,034	F =1,239	p =0,307
**Follow-fhit Anteriorlar (%)**	96,24 ± 4,59		92,39 ± 5,72		87,74 ± 10,5		91,59 ± 9,54		η =0,144	F =2,111	p =0,113
**Grupiçi Karşılaştırma (F^b)**	F =11,449	p =0	F =0,592	p =0,558	F =2,184	p =0,125	F =1,984	p =0,15			
Pre-Post	p =0,12		p =0,88		p =0,142		p =0,821				
Pre-Follow	p =0,022		p =0,837		p =0,124		p =1				
Post-Follow	p =1		p =1		p =0,258		p =0,357				

Ort: ortalama, ss: standart sapma, F: iki yönlü karma model ANOVA, F^b: Tekrarlayan Ölçümlerde ANOVA t testi, η^2; eta katsayısı, *p<0,05: istatistiksel anlamlılık vardır

Projeye dahil edilen DSBS tanılı katılımcılarda fHIT anterior kanal ölçümlerinin değişimlerinde gruplar arasında istatistiksel olarak anlamlı farklılık bulundu (p<0,05). Gruplar arası zamana göre değişimde elde edilen kısmi eta kare (η^2) değeri 0.144, etkileşimin toplam varyansın yaklaşık %14.4'ünü açıkladığını göstermektedir. Grup zaman etkileşimi zamanın her grupta etkisinin aynı olmadığını göstermektedir.

Grup içi zamana göre karşılaştırmalarda;

- VR+BDT grubunda ölçümlerin zamana göre uygulama öncesi ve sonrası değişimi istatistiksel olarak anlamlı bulundu (p<0.05).
    - pre ile follow arasındaki değişim istatistiksel olarak anlamlı bulundu (p<0.05).
- VR grubunda ölçümlerin zamana göre uygulama öncesi ve sonrası değişimi istatistiksel olarak anlamlı bulunmadı (p>0.05).
- BDT grubunda ölçümlerin zamana göre uygulama öncesi ve sonrası değişimi istatistiksel olarak anlamlı bulunmadı (p>0.05).
- Aktif kontrol grubunda ölçümlerin zamana göre uygulama öncesi ve sonrası değişimi istatistiksel olarak anlamlı bulunmadı (p>0.05). (Bkz. Tablo 4).

Tablo 5. Grupların fHIT Posterior Kanal Skorlarının Zaman İçinde Değişiminin Analizi (Pre–Post–Follow-Up)

Değişken	VR+BDT		VR		BDT		Aktif Kontrol		Gruplar arası Karşılaştırma	Gruplararası Bağımsız Karşılaştırma	
	Ort ± ss		Ort ± ss		Ort ± ss		Ort ± ss			F^a	p^a
**Pre-fHIT Posteriorlar**	81,93 ± 8,32		85,92 ± 13,88		78,25 ± 26,2		96,12 ± 7,31		F =3,027	F =2,961	p =0,043
**Post-fHIT Posteriorlar**	96,1 ± 4,44		94,98 ± 4,87		89,88 ± 12,34		88,63 ± 15,44		p =0,01	F =1,393	p =0,258
**Follow-fHIT Posteriorlar**	94,69 ± 3,61		92,26 ± 3,91		86,39 ± 10,39		92,38 ± 9,82		η =0,174	F =2,4	p =0,081
**Grupiçi Karşılaştırma (F^b)**	F =17,275	p =0	F =1,122	p =0,335	F =2,019	p =0,145	F =4,638	p =0,015			

Pre-Post	p =0,07	p =0,421	p =0,151	p =0,551
Pre-Follow	p =0,012	p =0,411	p =0,145	p =1
Post-Follow	p =1	p =0,521	p =0,211	p =0,132

Ort: ortalama, ss: standart sapma, F: iki yönlü karma model ANOVA, F^b: Tekrarlayan Ölçümlerde ANOVA t testi, η^2; eta katsayısı, *p<0,05: istatistiksel anlamlılık vardır

Projeye dahil edilen DSBS tanılı katılımcılarda fHIT posterior ölçümlerinin değişimlerinde gruplar arasında istatistiksel olarak anlamlı farklılık bulundu ($p<0,05$). Gruplar arası zamana göre değişimde elde edilen kısmi eta kare (η^2) değeri 0.174, etkileşimin toplam varyansın yaklaşık %17.4'ünü açıkladığını göstermektedir. Grup zaman etkileşimi zamanın her grupta etkisinin aynı olmadığını göstermektedir.

Grup içi zamana göre karşılaştırmalarda;

- VR+BDT grubunda ölçümlerin zamana göre uygulama öncesi ve sonrası değişimi istatistiksel olarak anlamlı bulundu ($p<0.05$).
  - Pre ile post, pre ile follow arasındaki değişim istatistiksel olarak anlamlı bulundu ($p<0.05$).
- VR grubunda ölçümlerin zamana göre uygulama öncesi ve sonrası değişimi istatistiksel olarak anlamlı bulunmadı ($p>0.05$).
- BDT grubunda ölçümlerin zamana göre uygulama öncesi ve sonrası değişimi istatistiksel olarak anlamlı bulunmadı ($p>0.05$).
- Aktif kontrol grubunda ölçümlerin zamana göre uygulama öncesi ve sonrası değişimi istatistiksel olarak anlamlı bulunmadı ($p>0.05$). (Bkz. Tablo 5).

Tablo 6. Grupların BDEE Fiziksel Alt Ölçek Skorlarının Pre–Post–Follow-Up Karşılaştırması

Değişken	VR+BDT	VR	BDT	Aktif Kontrol	Gruplar arası	Gruplararası Bağımsız

					Karşılaştırma	Karşılaştırma	
	**Ort ± ss**	**Ort ± ss**	**Ort ± ss**	**Ort ± ss**		**F^a**	**p^a**
**Pre-BDEE Fiziksel**	12,27 ± 2,28	11 ± 2,24	15,92 ± 5,25	18,46 ± 2,63	F =10,979	F =12,233	p =0
**Post-BDEE Fiziksel**	4,27 ± 2,1	5,45 ± 1,51	12,08 ± 4,98	4,69 ± 5,95	p =0	F =9,035	p =0
**FOllow-BDEE Fiziksel**	4,45 ± 1,97	8,23 ± 1,17	14 ± 4,16	6,07 ± 5,24	η =0,434	F =15,476	p =0
**Grupiçi Karşılaştırma (F^b)**	F =27,847 p =0	F =14,218 p =0	F =7,412 p =0,002	F =66,5 p =0			
Pre-Post	p =0	p =0,004	p =0,053	p =0			
Pre-Follow	p =0	p =0,096	p =0,351	p =0			
Post-Follow	p =1	p =0	p =0,001	p =0,02			

BDEE: Baş dönmesi engellilik envanteri. Ort: ortalama, ss: standart sapma, F: iki yönlü karma model ANOVA, F^b: Tekrarlayan Ölçümlerde ANOVA t testi, η^2; eta katsayısı, *p<0,05: istatistiksel anlamlılık vardır

Bu projeye dahil edilen DSBS tanılı katılımcılarda BDEE fiziksel ölçümlerinin değişimlerinde gruplar arasında istatistiksel olarak anlamlı farklılık bulundu (p<0,05). Gruplar arası zamana göre değişimde elde edilen kısmi eta kare (η^2) değeri 0.434, etkileşimin toplam varyansın yaklaşık %43.4'ünü açıkladığını göstermektedir. Grup zaman etkileşimi zamanın her grupta etkisinin aynı olmadığını göstermektedir.

Grup içi zamana göre karşılaştırmalarda;

- VR+BDT grubunda ölçümlerin zamana göre uygulama öncesi ve sonrası değişimi istatistiksel olarak anlamlı bulundu (p<0.05).
    - Pre ile post, pre ile follow arasındaki değişim istatistiksel olarak anlamlı bulundu (p<0.05).
- VR grubunda ölçümlerin zamana göre uygulama öncesi ve sonrası değişimi istatistiksel olarak anlamlı bulundu (p<0.05).
    - Pre ile post, post ile follow arasındaki değişim istatistiksel olarak anlamlı bulundu (p<0.05).

- BDT grubunda ölçümlerin zamana göre uygulama öncesi ve sonrası değişimi istatistiksel olarak anlamlı bulundu ($p<0.05$).
  - Post ile follow arasındaki değişim istatistiksel olarak anlamlı bulundu ($p<0.05$).
- Aktif kontrol grubunda ölçümlerin zamana göre uygulama öncesi ve sonrası değişimi istatistiksel olarak anlamlı bulundu ($p<0.05$).
  - Pre ile post,pre ile follow, post ile follow arasındaki değişim istatistiksel olarak anlamlı bulundu ($p<0.05$). (Bkz. Tablo 6).

Tablo 7. Grupların BDEE Fonksiyonel Alt Ölçek Skorlarının Pre–Post–Follow-Up Karşılaştırması

Değişken	VR+BDT	VR	BDT	Aktif Kontrol	Gruplar arası Karşılaştırma	Gruplararası Bağımsız Karşılaştırma	
	Ort ± ss	Ort ± ss	Ort ± ss	Ort ± ss		F^a	p^a
**Pre-BDEE Fonksiyonel**	15,73 ± 1,79	13,55 ± 1,75	20,67 ± 4,64	24,08 ± 3,25	F =45,787	F =27,268	p =0
**Post-BDEE Fonksiyonel**	4,91 ± 3,81	7,73 ± 2,53	17,17 ± 5,11	4,46 ± 4,22	p =0	F =25,467	p =0
**Follow-BDEE Fonksiyonel**	5,36 ± 3,41	10,64 ± 1,7	18,92 ± 3,95	6,42 ± 3,94	η =0,762	F =38,615	p =0
**Grupiçi Karşılaştırma (F^b)**	F =84,204 p =0	F =14,57 p =0	F =5,752 p =0,006	F =262,654 p =0			
Pre-Post	p =0	p =0	p =0,016	p =0			
Pre-Follow	p =0	p =0,006	p =0,134	p =0			
Post-Follow	p =1	p =0	p =0,004	p =0,001			

BDEE: Baş dönmesi engellilik envanteri. Ort: ortalama, ss: standart sapma, F: iki yönlü karma model ANOVA, F^b: Tekrarlayan Ölçümlerde ANOVA t testi, η^2; eta katsayısı, *$p<0,05$: istatistiksel anlamlılık vardır

Bu projeye dahil edilen DSBS tanılı katılımcılarda DHI Fonksiyonel ölçümlerinin değişimlerinde gruplar arasında istatistiksel olarak anlamlı farklılık bulundu ($p<0,05$). Gruplar arası zamana göre değişimde elde edilen kısmi eta kare (η^2)

değeri 0.762, etkileşimin toplam varyansın yaklaşık %76.2'sini açıkladığını göstermektedir. Grup zaman etkileşimi zamanın her grupta etkisinin aynı olmadığını göstermektedir.

Grup içi zamana göre karşılaştırmalarda;

- VR+BDT grubunda ölçümlerin zamana göre uygulama öncesi ve sonrası değişimi istatistiksel olarak anlamlı bulundu ($p<0.05$).
    - Pre ile post, pre ile follow arasındaki değişim istatistiksel olarak anlamlı bulundu ($p<0.05$).
- VR grubunda ölçümlerin zamana göre uygulama öncesi ve sonrası değişimi istatistiksel olarak anlamlı bulundu ($p<0.05$).
    - Pre ile post, pre ile follow, post ile follow arasındaki değişim istatistiksel olarak anlamlı bulundu ($p<0.05$).
- BDT grubunda ölçümlerin zamana göre uygulama öncesi ve sonrası değişimi istatistiksel olarak anlamlı bulundu ($p<0.05$).
    - Pre ile post, Post ile follow arasındaki değişim istatistiksel olarak anlamlı bulundu ($p<0.05$).
- Aktif kontrol grubunda ölçümlerin zamana göre uygulama öncesi ve sonrası değişimi istatistiksel olarak anlamlı bulundu ($p<0.05$).
    - Pre ile post, pre ile follow, post ile follow arasındaki değişim istatistiksel olarak anlamlı bulundu ($p<0.05$). (Bkz. Tablo 7).

Tablo 8. Grupların BDEE Duygusal Alt Ölçek Skorlarının Pre–Post–Follow-Up Karşılaştırmas

**Değişken**	**VR+BDT**	**VR**	**BDT**	**Aktif Kontrol**	**Gruplar arası Karşılaştırma**	**Gruplararası Bağımsız Karşılaştırma**	
	**Ort ± ss**	**Ort ± ss**	**Ort ± ss**	**Ort ± ss**		**F^a**	**p^a**
**Pre-BDEE Duyusal**	13,45 ± 2,02	13 ± 2,14	20,58 ± 6,08	22,31 ± 4,03	F =32,96	F =17,004	p =0

**Post-BDEE Duyusal**	6,09 ± 2,26	8,36 ± 2,8	13,83 ± 5,8	4,54 ± 3,23	p =0	F =13,873	p =0
**Follow-BDEE Duyusal**	6,36 ± 1,96	10,68 ± 1,91	17,21 ± 5,56	6,32 ± 2,88	η =0,697	F =26,426	p =0
**Grupiçi Karşılaştırma (F^b)**	F =35,826 p =0	F =15,073 p =0	F =34,852 p =0	F =186,475 p =0			
Pre-Post	p =0	p =0,001	p =0	p =0			
Pre-Follow	p =0	p =0,054	p =0,002	p =0			
Post-Follow	p =1	p =0	p =0	p =0			

BDEE: Baş dönmesi engellilik envanteri. Ort: ortalama, ss: standart sapma, F: iki yönlü karma model ANOVA, F^b: Tekrarlayan Ölçümlerde ANOVA t testi, η^2; eta katsayısı, *p<0,05: istatistiksel anlamlılık vardır

Çalışmaya alınan katılımcılarda BDEE duyusal ölçümlerinin değişimlerinde gruplar arasında istatistiksel olarak anlamlı farklılık bulundu ($p<0,05$). Gruplar arası zamana göre değişimde elde edilen kısmi eta kare (η^2) değeri 0.697, etkileşimin toplam varyansın yaklaşık %69.7'sini açıkladığını göstermektedir. Grup zaman etkileşimi zamanın her grupta etkisinin aynı olmadığını göstermektedir.

Grup içi zamana göre karşılaştırmalarda;

- VR+BDT grubunda ölçümlerin zamana göre uygulama öncesi ve sonrası değişimi istatistiksel olarak anlamlı bulundu ($p<0.05$).
    - Pre ile post, pre ile follow arasındaki değişim istatistiksel olarak anlamlı bulundu ($p<0.05$).
- VR grubunda ölçümlerin zamana göre uygulama öncesi ve sonrası değişimi istatistiksel olarak anlamlı bulundu ($p<0.05$).
    - Pre ile post, post ile follow arasındaki değişim istatistiksel olarak anlamlı bulundu ($p<0.05$).
- BDT grubunda ölçümlerin zamana göre uygulama öncesi ve sonrası değişimi istatistiksel olarak anlamlı bulundu ($p<0.05$).

- Pre ile post, pre ile follow, post ile follow arasındaki değişim istatistiksel olarak anlamlı bulundu ($p<0.05$).

- Aktif kontrol grubunda ölçümlerin zamana göre uygulama öncesi ve sonrası değişimi istatistiksel olarak anlamlı bulundu ($p<0.05$).
  - Pre ile post, pre ile follow, post ile follow arasındaki değişim istatistiksel olarak anlamlı bulundu ($p<0.05$). (Bkz. Tablo 8).

Tablo 9. Grupların BDEE Toplam Skorlarının Zaman İçinde Değişiminin Analizi

Değişken	VR+BDT		VR		BDT		Aktif Kontrol		Gruplar arası Karşılaştırma	Gruplararası Bağımsız Karşılaştırma	
	Ort ± ss		Ort ± ss		Ort ± ss		Ort ± ss			F^a	p^a
**Pre-BDEE Toplam**	41,45 ± 3,5		37,55 ± 4,27		57,17 ± 14,48		64,85 ± 6,18		F =45,773	F =27,754	p =0
**Post-BDEE Toplam**	15,27 ± 7,68		21,55 ± 2,5		43,08 ± 12,75		13,69 ± 12,23		p =0	F =22,46	p =0
**Follow-BDEE Toplam**	15,45 ± 7,17		29,55 ± 2,96		50,13 ± 12,02		18,81 ± 11,05		η =0,762	F =34,181	p =0
**Grupiçi Karşılaştırma (F^b)**	F =100,438	p =0	F =29,802	p =0	F =25,188	p =0	F =286,395	p =0			
Pre-Post	p =0		p =0		p =0		p =0				
Pre-Follow	p =0		p =0,003		p =0,008		p =0				
Post-Follow	p =1		p =0		p =0		p =0				

Ort: ortalama, ss: standart sapma, F: iki yönlü karma model ANOVA, F^b: Tekrarlayan Ölçümlerde ANOVA t testi, η^2; eta katsayısı, *$p<0,05$: istatistiksel anlamlılık vardır

Projeye dahil edilen DSBS tanılı katılımcılarda BDEE toplam ölçümlerinin değişimlerinde gruplar arasında istatistiksel olarak anlamlı farklılık bulundu ($p<0,05$). Gruplar arası zamana göre değişimde elde edilen kısmi eta kare (η^2) değeri 0.762, etkileşimin toplam varyansın yaklaşık %76.2'sini açıkladığını göstermektedir. Grup zaman etkileşimi zamanın her grupta etkisinin aynı olmadığını göstermektedir.

Grup içi zamana göre karşılaştırmalarda;

- VR+BDT grubunda ölçümlerin zamana göre uygulama öncesi ve sonrası değişimi istatistiksel olarak anlamlı bulundu ($p<0.05$).
  - Pre ile post, pre ile follow arasındaki değişim istatistiksel olarak anlamlı bulundu ($p<0.05$).
- VR grubunda ölçümlerin zamana göre uygulama öncesi ve sonrası değişimi istatistiksel olarak anlamlı bulundu ($p<0.05$).
  - Pre ile post, pre ile follow, post ile follow arasındaki değişim istatistiksel olarak anlamlı bulundu ($p<0.05$).
- BDT grubunda ölçümlerin zamana göre uygulama öncesi ve sonrası değişimi istatistiksel olarak anlamlı bulundu ($p<0.05$).
  - Pre ile post, pre ile follow, post ile follow arasındaki değişim istatistiksel olarak anlamlı bulundu ($p<0.05$).
- Aktif kontrol grubunda ölçümlerin zamana göre uygulama öncesi ve sonrası değişimi istatistiksel olarak anlamlı bulundu ($p<0.05$).
  - Pre ile post, pre ile follow, post ile follow arasındaki değişim istatistiksel olarak anlamlı bulundu ($p<0.05$). (Bkz. Tablo 9).

Tablo 10. Grupların Beck Anksiyete Skorlarının Pre–Post–Follow-Up Değişimi

**Değişken**	**VR+BDT**		**VR**		**BDT**		**Aktif Kontrol**		**Gruplar arası Karşılaştırma**	**Gruplararası Bağımsız Karşılaştırma**	
	**Ort ± ss**		**Ort ± ss**		**Ort ± ss**		**Ort ± ss**			**F^a**	**p^a**
**Pre-Anksiyete**	21,18 ± 2,14		19,91 ± 2,59		36,67 ± 14,54		40,77 ± 10,38		F =4,938	F =15,416	p =0
**Post-Anksiyete**	4,24 ± 0,43		9,95 ± 1,29		24,17 ± 16,08		38,46 ± 8,99		p =0	F =31,722	p =0
**Follow-Anksiyete**	5,65 ± 2,42		10,68 ± 1,93		21,17 ± 10,12		39,54 ± 11,64		η =0,256	F =41,243	p =0
**Grupiçi Karşılaştırma (F^b)**	F =46,756	p =0	F =16,244	p =0	F =35,039	p =0	F =0,832	p =0,442			

Pre-Post	p =0	p =0	p =0	p =0,642
Pre-Follow	p =0	p =0,003	p =0	p =1
Post-Follow	p =1	p =1	p =0,919	p =1

Ort: ortalama, ss: standart sapma, F: iki yönlü karma model ANOVA, F^b: Tekrarlayan Ölçümlerde ANOVA t testi, η^2; eta katsayısı, *p<0,05: istatistiksel anlamlılık vardır

Projeye dahil edilen DSBS tanılı katılımcılarda Beck Anksiyete ölçümlerinin değişimlerinde gruplar arasında istatistiksel olarak anlamlı farklılık bulundu ($p<0,05$). Gruplar arası zamana göre değişimde elde edilen kısmi eta kare (η^2) değeri 0.418, etkileşimin toplam varyansın yaklaşık %41.8'ini açıkladığını göstermektedir. Grup zaman etkileşimi zamanın her grupta etkisinin aynı olmadığını göstermektedir.

Grup içi zamana göre karşılaştırmalarda;

- VR+BDT grubunda ölçümlerin zamana göre uygulama öncesi ve sonrası değişimi istatistiksel olarak anlamlı bulundu ($p<0.05$).
    - Pre ile post, pre ile follow arasındaki değişim istatistiksel olarak anlamlı bulundu ($p<0.05$).
- VR grubunda ölçümlerin zamana göre uygulama öncesi ve sonrası değişimi istatistiksel olarak anlamlı bulundu ($p<0.05$).
    - Pre ile post, pre ile follow arasındaki değişim istatistiksel olarak anlamlı bulundu ($p<0.05$).
- BDT grubunda ölçümlerin zamana göre uygulama öncesi ve sonrası değişimi istatistiksel olarak anlamlı bulundu ($p<0.05$).
    - Pre ile post, pre ile follow arasındaki değişim istatistiksel olarak anlamlı bulundu ($p<0.05$).
- Aktif kontrol grubunda ölçümlerin zamana göre uygulama öncesi ve sonrası değişimi istatistiksel olarak anlamlı bulunmadı ($p>0.05$). (Bkz. Tablo 10).

Tablo 11. Grupların PCL-5 Travma Belirti Skorlarının Pre–Post–Follow-Up Değişimi

Değişken	VR+BDT		VR		BDT		Aktif Kontrol		Gruplar arası Karşılaştırma	Gruplararası Bağımsız Karşılaştırma	
	Ort ± ss		Ort ± ss		Ort ± ss		Ort ± ss			F^a	p^a
**Pre-PCL-5**	20,55 ± 6,06		17,91 ± 5,92		20,67 ± 6,56		19,31 ± 6,82		F =3,545	F =0,459	p =0,712
**Post-PCL-5**	7,91 ± 3,88		4,27 ± 2,69		5,33 ± 3,45		3,62 ± 3,45		p =0,003	F =3,55	p =0,022
**Follow-PCL-5**	9,64 ± 4,15		11,09 ± 3,38		13 ± 3,86		5,18 ± 3,47		η =0,198	F =10,032	p =0
**Grupiçi Karşılaştırma (F^b)**	F =26,576	p =0	F =31,799	p =0	F =43,861	p =0	F =55,368	p =0			
Pre-Post	p =0		p =0		p =0		p =0				
Pre-Follow	p =0		p =0		p =0		p =0				
Post-Follow	p =0,143		p =0		p =0		p =0,151				

PCL-5: Travma Sonrası Stress Bozukluğu Kontrol Listesi Ort: ortalama, ss: standart sapma, F: iki yönlü karma model ANOVA, F^b: Tekrarlayan Ölçümlerde ANOVA t testi, η^2; eta katsayısı, *p<0,05: istatistiksel anlamlılık vardır

Projeye dahil edilen DSBS tanılı katılımcılarda PCL-5 ölçümlerinin değişimlerinde gruplar arasında istatistiksel olarak anlamlı farklılık bulundu (p<0,05). Gruplar arası zamana göre değişimde elde edilen kısmi eta kare (η^2) değeri 0.198, etkileşimin toplam varyansın yaklaşık %19.8'ini açıkladığını göstermektedir. Grup zaman etkileşimi zamanın her grupta etkisinin aynı olmadığını göstermektedir.

Grup içi zamana göre karşılaştırmalarda;

- VR+BDT grubunda ölçümlerin zamana göre uygulama öncesi ve sonrası değişimi istatistiksel olarak anlamlı bulundu (p<0.05).
  - Pre ile post, pre ile follow arasındaki değişim istatistiksel olarak anlamlı bulundu (p<0.05).
- VR grubunda ölçümlerin zamana göre uygulama öncesi ve sonrası değişimi istatistiksel olarak anlamlı bulundu (p<0.05).
  - Pre ile post, pre ile follow, post ile follow arasındaki değişim istatistiksel olarak anlamlı bulundu (p<0.05).

- BDT grubunda ölçümlerin zamana göre uygulama öncesi ve sonrası değişimi istatistiksel olarak anlamlı bulundu (p<0.05).
  - Pre ile post, pre ile follow, post ile follow arasındaki değişim istatistiksel olarak anlamlı bulundu (p<0.05).
- Aktif kontrol grubunda ölçümlerin zamana göre uygulama öncesi ve sonrası değişimi istatistiksel olarak anlamlı bulundu (p<0.05).
  - Pre ile post, pre ile follow arasındaki değişim istatistiksel olarak anlamlı bulundu (p<0.05). (Bkz. Tablo 11).

Tablo 12. Grupların Deprem Travma Ölçeği Skorlarının Zaman İçinde Değişimi

**Değişken**	**VR+BDT**	**VR**	**BDT**	**Aktif Kontrol**	**Gruplar arası Karşılaştırma**	**Gruplararası Bağımsız Karşılaştırma**	
	**Ort ± ss**	**Ort ± ss**	**Ort ± ss**	**Ort ± ss**		**F^a**	**p^a**
**Pre- Deprem Travma**	61,73 ± 10,62	62 ± 14,18	60,5 ± 10,27	56,08 ± 11,03	F =5,791	F =0,696	p =0,56
**Post-Deprem Travma**	20 ± 10,77	18,91 ± 9,78	12,58 ± 9,04	19,54 ± 13,82	p =0	F =1,167	p =0,333
**FOllow-Deprem Travma**	19,36 ± 8,96	40,45 ± 6,73	36,54 ± 5,88	23,19 ± 12,32	η =0,288	F =14,764	p =0
**Grupiçi Karşılaştırma (F^b)**	F =90,724 p =0	F =51,175 p =0	F =69,032 p =0	F =50,432 p =0			
Pre-Post	p =0	p =0	p =0	p =0			
Pre-Follow	p =0	p =0	p =0	p =0			
Post-Follow	p =1	p =0	p =0	p =0,198			

Ort: ortalama, ss: standart sapma, F: iki yönlü karma model ANOVA, F^b: Tekrarlayan Ölçümlerde ANOVA t testi, η^2; eta katsayısı, *p<0,05: istatistiksel anlamlılık vardır

Projeye dahil edilen DSBS tanılı katılımcılarda Deprem Travma ölçümlerinin değişimlerinde gruplar arasında istatistiksel olarak anlamlı farklılık bulundu (p<0,05). Gruplar arası zamana göre değişimde elde edilen kısmi eta kare (η^2) değeri

0.288, etkileşimin toplam varyansın yaklaşık %28.8'ini açıkladığını göstermektedir. Grup zaman etkileşimi zamanın her grupta etkisinin aynı olmadığını göstermektedir.

Grup içi zamana göre karşılaştırmalarda;

- VR+BDT grubunda ölçümlerin zamana göre uygulama öncesi ve sonrası değişimi istatistiksel olarak anlamlı bulundu (p<0.05).
  - Pre ile post, pre ile follow arasındaki değişim istatistiksel olarak anlamlı bulundu (p<0.05).
- VR grubunda ölçümlerin zamana göre uygulama öncesi ve sonrası değişimi istatistiksel olarak anlamlı bulundu (p<0.05).
  - Pre ile post, pre ile follow, post ile follow arasındaki değişim istatistiksel olarak anlamlı bulundu (p<0.05).
- BDT grubunda ölçümlerin zamana göre uygulama öncesi ve sonrası değişimi istatistiksel olarak anlamlı bulundu (p<0.05).
  - Pre ile post, pre ile follow, post ile follow arasındaki değişim istatistiksel olarak anlamlı bulundu (p<0.05).
- Aktif kontrol grubunda ölçümlerin zamana göre uygulama öncesi ve sonrası değişimi istatistiksel olarak anlamlı bulundu (p<0.05).
  - Pre ile post, post ile follow arasındaki değişim istatistiksel olarak anlamlı bulundu (p<0.05). (Bkz. Tablo 12).

Tablo 13. Grupların Vertigo Dizability (VerDiz) Yorgunluk–Korku ve Sosyal Stres Alt Ölçek Skorlarının Zaman İçindeki Değişimine İlişkin Pre–Post–Follow-Up Analizi

Değişken	VR+BDT	VR	BDT	Aktif Kontrol	Gruplar arası Karşılaştırma	Gruplararası Bağımsız Karşılaştırma	
	Ort ± ss	Ort ± ss	Ort ± ss	Ort ± ss		F^a	p^a
Pre-VerDiz-YK	29,73 ± 15,83	35,18 ± 18,36	32,25 ± 17,31	33,69 ± 13,03	F =1,108	F =0,23	p =0,875
Post-VerDiz-YK	62,36 ± 23,79	71,45 ± 17,28	66,92 ± 21,47	67,85 ± 14,07	p =0,365	F =0,412	p =0,745
Follow-	58,91 ± 23,82	53,32 ± 10,67	49,58 ± 12,9	64,43 ± 12,92	η =0,072	F =2,106	p =0,113

**VerDiz-YK**							
**Grupiçi Karşılaştırma (F^b)**	F =11,313 p =0	F =16,72 p =0	F =16,661 p =0	F =14,965 p =0			
Pre-Post	p =0,001	p =0	p =0	p =0			
Pre-Follow	p =0	p =0,018	p =0,018	p =0			
Post-Follow	p =0,814	p =0	p =0	p =0,713			
**Pre-VerDiz-SS**	21,45 ± 8,47	19,64 ± 8,3	19,5 ± 10,92	26,69 ± 7,49	F =1,318	F =1,801	p =0,161
**Post-VerDiz-SS**	42,64 ± 7,88	35 ± 7,58	36,83 ± 11,09	44,31 ± 10,08	p =0,258	F =2,709	p =0,057
**Follow-VerDiz-SS**	37,73 ± 7,28	27,32 ± 5,24	28,17 ± 7,2	42,55 ± 8,96	η =0,084	F =12,258	p =0
**Grupiçi Karşılaştırma (F^b)**	F =14,087 p =0	F =9,477 p =0	F =13,159 p =0	F =16,892 p =0			
Pre-Post	p =0	p =0,002	p =0	p =0			
Pre-Follow	p =0	p =0,046	p =0,014	p =0			
Post-Follow	p =0,024	p =0	p =0	p =0,852			

YK: Yorgunluk ve korku; SS: Sosyal stres; Ort: ortalama, ss: standart sapma, F: iki yönlü karma model ANOVA, F^b: Tekrarlayan Ölçümlerde ANOVA t testi, η^2; eta katsayısı, *$p<0,05$: istatistiksel anlamlılık vardır

- Grupların VerDiz-YK (Yorgunluk–Korku) ve VerDiz-SS (Sosyal Stres) alt ölçek skorlarına ilişkin sonuçlar Tablo X'te sunulmuştur. Her iki alt ölçekte de gruplar arasında zamana bağlı değişim açısından istatistiksel olarak anlamlı bir farklılık saptanmamıştır (tüm karşılaştırmalarda $p>0.05$).
- Bununla birlikte grup içi zamana göre değerlendirmelerde her iki alt ölçekte de benzer bir örüntü görülmüştür. VR+BDT grubunda, hem VerDiz-YK hem de VerDiz-SS skorlarında uygulama öncesi–sonrası değişim anlamlı bulunmuş; VerDiz-YK için pre–post ve pre–follow karşılaştırmaları, VerDiz-SS için ise pre–post, pre–follow ve post–follow karşılaştırmaları istatistiksel olarak anlamlıdır (tümünde $p<0.05$).
- VR grubunda, her iki alt ölçekte de zamana bağlı değişim anlamlıdır; VerDiz-YK için pre–post, pre–follow ve post–follow, VerDiz-SS için de aynı üç karşılaştırma anlamlı farklılık göstermiştir ($p<0.05$).

- BDT grubunda, her iki alt ölçekte uygulama öncesi–sonrası değişim anlamlıdır; VerDiz-YK'da pre–post, pre–follow ve post–follow değişimleri, VerDiz-SS'de ise pre–post, pre–follow ve post–follow değişimleri anlamlı bulunmuştur (p<0.05).
- Aktif kontrol grubunda, VerDiz-YK ve VerDiz-SS her iki alt ölçekte de uygulama öncesi–sonrası değişim anlamlıdır; VerDiz-YK'da pre–post ve pre–follow farklılıkları, VerDiz-SS'de ise pre–post ve pre–follow farklılıkları istatistiksel olarak anlamlıdır (p<0.05). (Bkz. Tablo 13).

Tablo 14. Duyusal Etkileşimli Klinik Denge Testi (CTSIB) Vestibüler Skorlarının Gruplar Arası ve Zaman Noktalarına Göre (Pre–Post–Follow-up) Karşılaştırılması.

**Değişken**	**VR+BDT**	**VR**	**BDT**	**Aktif Kontrol**	**Gruplar arası Karşılaştırma**	**Gruplararası Bağımsız Karşılaştırma**	
	**Ort ± ss**	**Ort ± ss**	**Ort ± ss**	**Ort ± ss**		**F^a**	**p^a**
**Pre-CTSIB vestibüler skor**	74,55 ± 17,31	65,73 ± 22,49	67,67 ± 25,16	60,54 ± 25,66	F =1,177	F =0,745	p =0,531
**Post-CTSIB vestibüler skor**	95,44 ± 2,52	81,64 ± 14,11	88,92 ± 8,04	65,54 ± 25,76	p =0,326	F =8,123	p =0
**Follow-CTSIB vestibüler skor**	93,35 ± 1,96	76,86 ± 14,26	82,54 ± 7,51	63,04 ± 18,94	η =0,076	F =11,796	p =0
**Grupiçi Karşılaştırma (F^b)**	F =16,046 p =0	F =2,346 p =0,108	F =4,566 p =0,016	F =0,42 p =0,66			
Pre-Post	p =0,043	p =0,174	p =0,028	p =1			
Pre-Follow	p =0,003	p =0,121	p =0,015	p =1			
Post-Follow	p =1	p =0,434	p =0,133	p =1			

CTSIB: Clinical Test of Sensory Interaction on Balance/ Duyusal Etkileşimli Klinik Denge Testi COrt: ortalama, ss: standart sapma, F: iki yönlü karma model ANOVA, F^b: Tekrarlayan Ölçümlerde ANOVA t testi, η^2; eta katsayısı, *p<0,05: istatistiksel anlamlılık vardır.

Projeye dahil edilen DSBS’li katılımcılarda CTSIB vestibüler skor ölçümlerinin değişimlerinde gruplar arasında istatistiksel olarak anlamlı farklılık bulunmadı (p>0.05).

Grup içi zamana göre karşılaştırmalarda;

- VR+BDT grubunda ölçümlerin zamana göre uygulama öncesi ve sonrası değişimi istatistiksel olarak anlamlı bulundu (p<0.05).
    - Pre ile post, pre ile follow arasındaki değişim istatistiksel olarak anlamlı bulundu (p<0.05).
- VR grubunda ölçümlerin zamana göre uygulama öncesi ve sonrası değişimi istatistiksel olarak anlamlı bulunmadı (p>0.05).
- BDT grubunda ölçümlerin zamana göre uygulama öncesi ve sonrası değişimi istatistiksel olarak anlamlı bulundu (p<0.05).
    - Pre ile post, pre ile follow arasındaki değişim istatistiksel olarak anlamlı bulundu (p<0.05).
- Aktif kontrol grubunda ölçümlerin zamana göre uygulama öncesi ve sonrası değişimi istatistiksel olarak anlamlı bulunmadı (p>0.05). (Bkz. Tablo 14).

Tablo 15. Stabilite Limitleri (LOS) Reaksiyon Süresi Sonuçlarının Gruplar Arasında ve Zaman Noktalarına Göre (Pre–Post–Follow-up) Karşılaştırılması.

**Değişken**	**VR+BDT**	**VR**	**BDT**	**Aktif Kontrol**	**Gruplar arası Karşılaştırma**	**Gruplararası Bağımsız Karşılaştırma**	
	**Ort ± ss**	**Ort ± ss**	**Ort ± ss**	**Ort ± ss**		**F^a**	**p^a**
**Pre-LOS reaksiyon süre ort.**	0,42 ± 0,12	0,52 ± 0,22	0,42 ± 0,19	0,57 ± 0,27	F =2,682	F =1,56	p =0,213

**Post-LOS reaksiyon süre ort.**	0,11 ± 0,03		0,17 ± 0,08		0,2 ± 0,09		0,47 ± 0,24		p =0,02	F =15,839	p =0
**Follow-LOS reaksiyon süre ort.**	0,14 ± 0,04		0,28 ± 0,08		0,27 ± 0,1		0,52 ± 0,17		η =0,158	F =25,12	p =0
**Grupiçi Karşılaştırma (F^b)**	F =63,005	p =0	F =18,368	p =0	F =7,972	p =0,001	F =1,611	p =0,212			
Pre-Post	p =0,001		p =0		p =0,016		p =0,472				
Pre-Follow	p =0		p =0		p =0,004		p =0,713				
Post-Follow	p =1		p =0,01		p =0,138		p =0,297				

LOS: Limits of stability/ stabilite limitleri; Ort: ortalama, ss: standart sapma, F: iki yönlü karma model ANOVA, F^b: Tekrarlayan Ölçümlerde ANOVA t testi, η^2; eta katsayısı, *p<0,05: istatistiksel anlamlılık vardır.

Projeye dahil edilen DSBS'li katılımcılarda **LOS reaksiyon süre ort.**ölçümlerinin değişimlerinde gruplar arasında istatistiksel olarak anlamlı farklılık bulundu (p<0,05). Gruplar arası zamana göre değişimde elde edilen kısmi eta kare (η^2) değeri 0.158, etkileşimin toplam varyansın yaklaşık %15.8'ini açıkladığını göstermektedir. Grup zaman etkileşimi zamanın her grupta etkisinin aynı olmadığını göstermektedir.

Grup içi zamana göre karşılaştırmalarda;

- VR+BDT grubunda ölçümlerin zamana göre uygulama öncesi ve sonrası değişimi istatistiksel olarak anlamlı bulundu (p<0.05).
  - Pre ile post, pre ile follow arasındaki değişim istatistiksel olarak anlamlı bulundu (p<0.05).
- VR grubunda ölçümlerin zamana göre uygulama öncesi ve sonrası değişimi istatistiksel olarak anlamlı bulundu (p<0.05).
  - Pre ile post, pre ile follow, post ile follow arasındaki değişim istatistiksel olarak anlamlı bulundu (p<0.05).
- BDT grubunda ölçümlerin zamana göre uygulama öncesi ve sonrası değişimi istatistiksel olarak anlamlı bulundu (p<0.05).

- Pre ile post, pre ile follow arasındaki değişim istatistiksel olarak anlamlı bulundu ($p<0.05$).
- Aktif kontrol grubunda ölçümlerin zamana göre uygulama öncesi ve sonrası değişimi istatistiksel olarak anlamlı bulunmadı ($p>0.05$). (Bkz. Tablo 15).

Tablo 16. Stabilite Limitleri (LOS) Movement Velocity/Hareket Hızı Sonuçlarının Gruplar Arasında ve Zaman Noktalarına Göre (Pre–Post–Follow-up) Karşılaştırılması.

Değişken	VR+BDT		VR		BDT		Aktif Kontrol		Gruplar arası Karşılaştırma	Gruplararası Bağımsız Karşılaştırma	
	Ort ± ss		Ort ± ss		Ort ± ss		Ort ± ss			F^a	p^a
**Pre-LOS hareket hızı ort**	1,5 ± 0,57		1,42 ± 0,54		1,46 ± 0,76		1,64 ± 0,93		F =0,621	F =0,22	p =0,882
**Post-LOS hareket hızı ort**	2,49 ± 0,63		2,02 ± 0,76		1,95 ± 0,78		2,24 ± 1,13		p =0,713	F =0,914	p =0,442
**Follow-LOS hareket hızı ort**	2,39 ± 0,58		1,84 ± 0,51		1,8 ± 0,59		1,94 ± 0,6		η =0,042	F =2,537	p =0,069
**Grupiçi Karşılaştırma (F^b)**	F =19,111	p =0	F =1,83	p =0,173	F =1,311	p =0,28	F =2,658	p =0,082			
Pre-Post	p =0,032		p =0,325		p =0,517		p =0,254				
Pre-Follow	p =0,001		p =0,227		p =0,392		p =0,505				
Post-Follow	p =1		p =0,7		p =0,936		p =0,107				

LOS: Limits of stability/ stabilite limitleri ;Ort: ortalama, ss: standart sapma, F: iki yönlü karma model ANOVA, F^b: Tekrarlayan Ölçümlerde ANOVA t testi, η^2; eta katsayısı, *$p<0,05$: istatistiksel anlamlılık vardır

Projeye dahil edilen DSBS'li katılımcılarda **LOS Mean velocity ort** ölçümlerinin değişimlerinde gruplar arasında istatistiksel olarak anlamlı farklılık bulunmadı ($p>0.05$).

Grup içi zamana göre karşılaştırmalarda;

- VR+BDT grubunda ölçümlerin zamana göre uygulama öncesi ve sonrası değişimi istatistiksel olarak anlamlı bulundu ($p<0.05$).

- Pre ile post, pre ile follow arasındaki değişim istatistiksel olarak anlamlı bulundu (p<0.05).

- VR grubunda ölçümlerin zamana göre uygulama öncesi ve sonrası değişimi istatistiksel olarak anlamlı bulunmadı (p>0.05).
- BDT grubunda ölçümlerin zamana göre uygulama öncesi ve sonrası değişimi istatistiksel olarak anlamlı bulunmadı (p>0.05).
- Aktif kontrol grubunda ölçümlerin zamana göre uygulama öncesi ve sonrası değişimi istatistiksel olarak anlamlı bulunmadı (p>0.05). (Bkz. Tablo 16).

Tablo 17. Stabilite Limitleri (LOS) Uç Nokta Sapması/End Point Excursion Sonuçlarının Gruplar Arasında ve Zaman Noktalarına Göre (Pre–Post–Follow-up) Karşılaştırılması.

**Değişken**	**VR+BDT**	**VR**	**BDT**	**Aktif Kontrol**	**Gruplar arası Karşılaştırma**	**Gruplararası Bağımsız Karşılaştırma**	
	**Ort ± ss**	**Ort ± ss**	**Ort ± ss**	**Ort ± ss**		**F^a**	**p^a**
**Pre-LOS Uç nokta sapması**	24,97 ± 10,28	27,39 ± 7,48	23,28 ± 11,74	21,95 ± 8,24	F =23,081	F =0,705	p =0,555
**Post-LOS Uç nokta sapması**	81,19 ± 2,58	69,42 ± 3,7	65,97 ± 10,89	43,6 ± 12,57	p =0	F =38,034	p =0
**Follow-LOS Uç nokta sapması**	75,57 ± 2,44	56,81 ± 1,97	53,16 ± 8,84	32,78 ± 8,42	η =0,617	F =87,691	p =0
**Grupiçi Karşılaştırma (F^b)**	F =408,609 p =0	F =63,124 p =0	F =71,03 p =0	F =50,755 p =0			
Pre-Post	p =0	p =0	p =0	p =0			
Pre-Follow	p =0	p =0	p =0	p =0			
Post-Follow	p =0	p =0	p =0	p =0			

LOS: Limits of stability/ stabilite limitleri; Ort: ortalama, ss: standart sapma, F: iki yönlü karma model ANOVA, F^b: Tekrarlayan Ölçümlerde ANOVA t testi, η^2; eta katsayısı, *p<0,05: istatistiksel anlamlılık vardır.

Projeye dahil edilen DSBS'li katılımcılarda LOS End Point Excursion ölçümlerinin değişimlerinde gruplar arasında istatistiksel olarak anlamlı farklılık bulundu ($p<0,05$). Gruplar arası zamana göre değişimde elde edilen kısmi eta kare (η^2) değeri 0.617, etkileşimin toplam varyansın yaklaşık %61.7'sini açıkladığını göstermektedir. Grup zaman etkileşimi zamanın her grupta etkisinin aynı olmadığını göstermektedir.

Grup içi zamana göre karşılaştırmalarda;

- VR+BDT grubunda ölçümlerin zamana göre uygulama öncesi ve sonrası değişimi istatistiksel olarak anlamlı bulundu ($p<0.05$).
    - Pre ile post, pre ile follow, post ile follow arasındaki değişim istatistiksel olarak anlamlı bulundu ($p<0.05$).
- VR grubunda ölçümlerin zamana göre uygulama öncesi ve sonrası değişimi istatistiksel olarak anlamlı bulundu ($p<0.05$).
    - Pre ile post, pre ile follow, post ile follow arasındaki değişim istatistiksel olarak anlamlı bulundu ($p<0.05$).
- BDT grubunda ölçümlerin zamana göre uygulama öncesi ve sonrası değişimi istatistiksel olarak anlamlı bulundu ($p<0.05$).
    - Pre ile post, pre ile follow, post ile follow arasındaki değişim istatistiksel olarak anlamlı bulundu ($p<0.05$).
- Aktif kontrol grubunda ölçümlerin zamana göre uygulama öncesi ve sonrası değişimi istatistiksel olarak anlamlı bulundu ($p<0.05$).
    - Pre ile post, pre ile follow, post ile follow arasındaki değişim istatistiksel olarak anlamlı bulundu ($p<0.05$). (Bkz. Tablo 17.)

Tablo 18. Stabilite Limitleri (LOS) Maksimum Sapma Sonuçlarının Gruplar Arasında ve Zaman Noktalarına Göre (Pre–Post–Follow-up) Karşılaştırılması.

Değişken	VR+BDT	VR	BDT	Aktif Kontrol	Gruplar arası Karşılaştırma	Gruplararası Bağımsız Karşılaştırma

	Ort ± ss	Ort ± ss	Ort ± ss	Ort ± ss		F^a	p^a
**Pre-LOS Max exursion ort**	40,2 ± 9,68	40,15 ± 9,85	38,04 ± 12,71	28,47 ± 14,21	F =6,563	F =2,733	p =0,055
**Post-LOS Max exursion ort**	85,34 ± 2,19	75,98 ± 3,97	71,89 ± 13,08	47,65 ± 18,32	p =0	F =22,267	p =0
**Follow-LOS Max exursion ort**	80,83 ± 1,97	65,23 ± 4,36	61,74 ± 9,69	38,06 ± 9,66	η =0,314	F =68,334	p =0
**Grupiçi Karşılaştırma (F^b)**	F =218,26 p =0	F =33,885 p =0	F =32,995 p =0	F =11,169 p =0			
Pre-Post	p =0	p =0	p =0	p =0,001			
Pre-Follow	p =0	p =0	p =0	p =0,011			
Post-Follow	p =0,176	p =0	p =0	p =0			

LOS: Limits of stability/ stabilite limitleri; Ort: ortalama, ss: standart sapma, F: iki yönlü karma model ANOVA, F^b: Tekrarlayan Ölçümlerde ANOVA t testi, η^2; eta katsayısı, *p<0,05: istatistiksel anlamlılık vardır

Projeye dahil edilen DSBS'li katılımcılarda **Los Max exursion ort** ölçümlerinin değişimlerinde gruplar arasında istatistiksel olarak anlamlı farklılık bulundu (p<0,05). Gruplar arası zamana göre değişimde elde edilen kısmi eta kare (η^2) değeri 0.314, etkileşimin toplam varyansın yaklaşık %31.4'ünü açıkladığını göstermektedir. Grup zaman etkileşimi zamanın her grupta etkisinin aynı olmadığını göstermektedir.

Grup içi zamana göre karşılaştırmalarda;

- VR+BDT grubunda ölçümlerin zamana göre uygulama öncesi ve sonrası değişimi istatistiksel olarak anlamlı bulundu (p<0.05).
    - Pre ile post, pre ile follow arasındaki değişim istatistiksel olarak anlamlı bulundu (p<0.05).
- VR grubunda ölçümlerin zamana göre uygulama öncesi ve sonrası değişimi istatistiksel olarak anlamlı bulundu (p<0.05).
    - Pre ile post, pre ile follow, post ile follow arasındaki değişim istatistiksel olarak anlamlı bulundu (p<0.05).

- BDT grubunda ölçümlerin zamana göre uygulama öncesi ve sonrası değişimi istatistiksel olarak anlamlı bulundu ($p<0.05$).
    - Pre ile post, pre ile follow, post ile follow arasındaki değişim istatistiksel olarak anlamlı bulundu ($p<0.05$).
- Aktif kontrol grubunda ölçümlerin zamana göre uygulama öncesi ve sonrası değişimi istatistiksel olarak anlamlı bulundu ($p<0.05$).
    - Pre ile post, pre ile follow, post ile follow arasındaki değişim istatistiksel olarak anlamlı bulundu ($p<0.05$). (Bkz. Tablo 18.).

Tablo 19. Stabilite Limitleri (LOS) Yön Kontrolü Gruplar Arasında ve Zaman Noktalarına Göre (Pre–Post–Follow-up) Karşılaştırılması.

**Değişken**	**VR+BDT**		**VR**		**BDT**		**Aktif Kontrol**		**Gruplar arası Karşılaştırma**	**Gruplararası Bağımsız Karşılaştırma**	
	**Ort ± ss**		**Ort ± ss**		**Ort ± ss**		**Ort ± ss**			**F^a**	**p^a**
**Pre-LOS Yön Kontrolü**	34,35 ± 7,51		31,78 ± 12,78		35,02 ± 11,02		29,54 ± 11,86		F =11,818	F =0,639	p =0,594
**Post-LOS Yön Kontrolü**	83,33 ± 1,84		73,29 ± 5,17		71 ± 12,01		48,16 ± 16,02		p =0	F =23,437	p =0
**Follow-LOS Yön Kontrolü**	78,43 ± 1,8		60,84 ± 5,92		60,21 ± 8,76		38,85 ± 9,25		η =0,452	F =60,344	p =0
**Grupiçi Karşılaştırma (F^b)**	F =351,909	p =0	F =57,661	p =0	F =47,246	p =0	F =16,228	p =0			
Pre-Post	p =0		p =0		p =0		p =0				
Pre-Follow	p =0		p =0		p =0		p =0,004				
Post-Follow	p =0,047		p =0		p =0		p =0				

LOS: Limits of stability/ stabilite limitleri ;Ort: ortalama, ss: standart sapma, F: iki yönlü karma model ANOVA, F^b: Tekrarlayan Ölçümlerde ANOVA t testi, η^2; eta katsayısı, *$p<0,05$: istatistiksel anlamlılık vardır.

Projeye dahil edilen DSBS'li katılımcılarda LOS Directional Control ölçümlerinin değişimlerinde gruplar arasında istatistiksel olarak anlamlı farklılık bulundu ($p<0,05$). Gruplar arası zamana göre değişimde elde edilen kısmi eta kare (η^2) değeri

0.452, etkileşimin toplam varyansın yaklaşık %45.2'sini açıkladığını göstermektedir. Grup zaman etkileşimi zamanın her grupta etkisinin aynı olmadığını göstermektedir.

Grup içi zamana göre karşılaştırmalarda;

- VR+BDT grubunda ölçümlerin zamana göre uygulama öncesi ve sonrası değişimi istatistiksel olarak anlamlı bulundu ($p<0.05$).
  - Pre ile post, pre ile follow, post ile follow arasındaki değişim istatistiksel olarak anlamlı bulundu ($p<0.05$).
- VR grubunda ölçümlerin zamana göre uygulama öncesi ve sonrası değişimi istatistiksel olarak anlamlı bulundu ($p<0.05$).
  - Pre ile post, pre ile follow, post ile follow arasındaki değişim istatistiksel olarak anlamlı bulundu ($p<0.05$).
- BDT grubunda ölçümlerin zamana göre uygulama öncesi ve sonrası değişimi istatistiksel olarak anlamlı bulundu ($p<0.05$).
  - Pre ile post, pre ile follow, post ile follow arasındaki değişim istatistiksel olarak anlamlı bulundu ($p<0.05$).
- Aktif kontrol grubunda ölçümlerin zamana göre uygulama öncesi ve sonrası değişimi istatistiksel olarak anlamlı bulundu ($p<0.05$).
  - Pre ile post, pre ile follow, post ile follow arasındaki değişim istatistiksel olarak anlamlı bulundu ($p<0.05$). (Bkz. Tablo 19).

Tablo 20. Sübjektif Görsel Dikeylik Algısı (SVV) Testinin Gruplar Arasında ve Zaman Noktalarına Göre (Pre–Post–Follow-up) Karşılaştırılması.

**Değişken**	**VR+BDT**	**VR**	**BDT**	**Aktif Kontrol**	**Gruplar arası Karşılaştırma**	**Gruplararası Bağımsız Karşılaştırma**	
	**Ort ± ss**	**Ort ± ss**	**Ort ± ss**	**Ort ± ss**		**F^a**	**p^a**
**Pre-SVV ort.**	1,75 ± 2,03	0,38 ± 2,22	1,57 ± 1,95	1,2 ± 3,36	F =0,553	F =0,659	p =0,582

Değişken	VR+BDT	VR	BDT	Aktif Kontrol	Gruplar arası Karşılaştırma	F^a	p^a
**Post-SVV ort.**	0,36 ± 0,7	0,37 ± 0,98	0,99 ± 1,6	1,49 ± 3,13	p =0,766	F =0,967	p =0,417
**Follow-SVV ort.**	0,5 ± 0,57	0,37 ± 0,95	1,16 ± 1,22	1,35 ± 1,71	η =0,037	F =1,845	p =0,153
**Grupiçi Karşılaştırma (F^b)**	F =5,019 p =0,011	F =0 p =1	F =0,251 p =0,779	F =0,062 p =0,94			
Pre-Post	p =0,621	p =1	p =1	p =1			
Pre-Follow	p =0,19	p =1	p =1	p =1			
Post-Follow	p =1	p =1	p =1	p =1			

SVV: Sübjektif gösel dikeylik algısı/sübjective visual vertical Ort: ortalama, ss: standart sapma, F: iki yönlü karma model ANOVA, F^b: Tekrarlayan Ölçümlerde ANOVA t testi, η^2; eta katsayısı, *p<0,05: istatistiksel anlamlılık vardır

Projeye dahil edilen DSBS'li katılımcılarda **SVV ort.** ölçümlerinin değişimlerinde gruplar arasında istatistiksel olarak anlamlı farklılık bulunmadı (p>0.05). (Bkz. Tablo 20).

Grup içi zamana göre karşılaştırmalarda;

- VR+BDT grubunda ölçümlerin zamana göre uygulama öncesi ve sonrası değişimi istatistiksel olarak anlamlı bulunmadı (p>0.05).
- VR grubunda ölçümlerin zamana göre uygulama öncesi ve sonrası değişimi istatistiksel olarak anlamlı bulunmadı (p>0.05).
- BDT grubunda ölçümlerin zamana göre uygulama öncesi ve sonrası değişimi istatistiksel olarak anlamlı bulunmadı (p>0.05).
- Aktif kontrol grubunda ölçümlerin zamana göre uygulama öncesi ve sonrası değişimi istatistiksel olarak anlamlı bulunmadı (p>0.05).

Tablo 21. Dinamik Sübjektif Görsel Dikeylik Algısı (SVV) Testinin Gruplar Arasında ve Zaman Noktalarına Göre (Pre–Post–Follow-up) Karşılaştırılması.

**Değişken**	**VR+BDT**	**VR**	**BDT**	**Aktif Kontrol**	**Gruplar arası Karşılaştırma**	**Gruplararası Bağımsız Karşılaştırma**	
	**Ort ± ss**	**Ort ± ss**	**Ort ± ss**	**Ort ± ss**		**F^a**	**p^a**
**Pre-Dinamik SVV ort.**	-3,13 ± 7,72	-4,01 ± 6,39	-1,31 ± 4,91	-5,03 ± 8,19	F =0,102	F =0,637	p =0,595

**Post-Dinamik SVV ort.**	-1,14 ± 2,18		-2,93 ± 2,9		-1,06 ± 4,44		-3,08 ± 7,68		p =0,996	F =0,585	p =0,628
**Follow-Dinamik SVV ort.**	-1,34 ± 1,92		-3,25 ± 2,4		-1,13 ± 3,16		-4,05 ± 4,13		η =0,007	F =2,627	p =0,062
**Grupiçi Karşılaştırma (F^b)**	F =0,931	p =0,402	F =0,081	p =0,922	F =0,005	p =0,995	F =0,433	p =0,652			
Pre-Post	p =1		p =1		p =1		p =1				
Pre-Follow	p =1		p =1		p =1		p =1				
Post-Follow	p =1		p =1		p =1		p =1				

DSVV: Dinamik Sübjektif Görsel Dikeylik Algısı / dynamic sübjective visual vertical Ort: ortalama, ss: standart sapma, F: iki yönlü karma model ANOVA, F^b: Tekrarlayan Ölçümlerde ANOVA t testi, η^2; eta katsayısı, *p<0,05: istatistiksel anlamlılık vardır

Projeye dahil edilen DSBS'li katılımcılarda **Dinamik SVV ort.** ölçümlerinin değişimlerinde gruplar arasında istatistiksel olarak anlamlı farklılık bulunmadı (p>0.05).

Grup içi zamana göre karşılaştırmalarda;

- VR+BDT grubunda ölçümlerin zamana göre uygulama öncesi ve sonrası değişimi istatistiksel olarak anlamlı bulunmadı (p>0.05).
- VR grubunda ölçümlerin zamana göre uygulama öncesi ve sonrası değişimi istatistiksel olarak anlamlı bulunmadı (p>0.05).
- BDT grubunda ölçümlerin zamana göre uygulama öncesi ve sonrası değişimi istatistiksel olarak anlamlı bulunmadı (p>0.05).
- Aktif kontrol grubunda ölçümlerin zamana göre uygulama öncesi ve sonrası değişimi istatistiksel olarak anlamlı bulunmadı (p>0.05). (Bkz. Tablo 21).

Tablo 22. Rod and Frame /Çubuk Çerçeve Testinin Gruplar Arasında ve Zaman Noktalarına Göre (Pre–Post–Follow-up) Karşılaştırılması.

Değişken	VR+BDT		VR		BDT		Aktif Kontrol		Gruplar arası Karşılaştırma	Gruplararası Bağımsız Karşılaştırma	
	Ort ± ss		Ort ± ss		Ort ± ss		Ort ± ss			F^a	p^a
**Pre-RFT ort.**	6,54 ± 6,25		10,43 ± 5,96		11,66 ± 4,2		9,63 ± 5,35		F =1,545	F =1,807	p =0,16
**Post-RFT ort.**	2,58 ± 2,54		5,36 ± 2,47		7,09 ± 3,74		9,08 ± 4,34		p =0,173	F =7,649	p =0
**Follow-RFT ort.**	2,97 ± 2,39		6,88 ± 2,09		8,46 ± 3,41		9,35 ± 3,95		η =0,097	F =9,506	p =0
**Grupiçi Karşılaştırma (F^b)**	F =6,456	p =0,004	F =3,936	p =0,027	F =3,485	p =0,04	F =0,16	p =0,853			
Pre-Post	p =0,097		p =0,021		p =0,032		p =1				
Pre-Follow	p =0,026		p =0,026		p =0,04		p =1				
Post-Follow	p =1		p =0,039		p =0,057		p =1				

RFT: Çobuk-Çerçeve Testi/ Rod and the Frame; Ort: ortalama, ss: standart sapma, F: iki yönlü karma model ANOVA, F^b: Tekrarlayan Ölçümlerde ANOVA t testi, η^2; eta katsayısı, *p<0,05: istatistiksel anlamlılık vardır

Projeye dahil edilen DSBS'li katılımcılarda **RFT ort.**ölçümlerinin değişimlerinde gruplar arasında istatistiksel olarak anlamlı farklılık bulunmadı (p>0.05).

Grup içi zamana göre karşılaştırmalarda;

- VR+BDT grubunda ölçümlerin zamana göre uygulama öncesi ve sonrası değişimi istatistiksel olarak anlamlı bulundu (p<0.05).
  - Pre ile follow arasındaki değişim istatistiksel olarak anlamlı bulundu (p<0.05).
- VR grubunda ölçümlerin zamana göre uygulama öncesi ve sonrası değişimi istatistiksel olarak anlamlı bulundu (p<0.05).
  - Pre ile post, pre ile follow, post ile follow arasındaki değişim istatistiksel olarak anlamlı bulundu (p<0.05).

- BDT grubunda ölçümlerin zamana göre uygulama öncesi ve sonrası değişimi istatistiksel olarak anlamlı bulundu (p<0.05).
    - Pre ile post, pre ile follow arasındaki değişim istatistiksel olarak anlamlı bulundu (p<0.05).
- Aktif kontrol grubunda ölçümlerin zamana göre uygulama öncesi ve sonrası değişimi istatistiksel olarak anlamlı bulunmadı (p>0.05). (Bkz. Tablo 22).

Tablo 23. Visual Motion Sensitvity/Görsel Hareket Duyarlılığı (VMS) Testi Optokinetik Hız Referansı Parametresinin Gruplar Arasında ve Zamana Göre Karşılaştırılması.

**Değişken**	**VR+BDT**		**VR**		**BDT**		**Aktif Kontrol**		**Gruplar arası Karşılaştırma**	**Gruplararası Bağımsız Karşılaştırma**	
	**Ort ± ss**		**Ort ± ss**		**Ort ± ss**		**Ort ± ss**			**F^a**	**p^a**
**Pre-VMS hız referansı**	27,46 ± 25,75		15,26 ± 8,5		21,44 ± 7,48		46,71 ± 32,93		F =0,525	F =4,718	p =0,006
**Post-VMS hız referansı**	14,32 ± 12,64		11,06 ± 4,46		14,39 ± 4,88		47,1 ± 30,63		p =0,788	F =11,456	p =0
**Follow-VMS hız referansı**	15,63 ± 10,65		12,74 ± 3,96		16,5 ± 4,46		46,91 ± 23,53		η =0,036	F =16,694	p =0
**Grupiçi Karşılaştırma (F^b)**	F =3,411	p =0,043	F =0,123	p =0,885	F =0,425	p =0,657	F =0,002	p =0,998			
Pre-Post	p =0,44		p =1		p =1		p =1				
Pre-Follow	p =0,145		p =1		p =1		p =1				
Post-Follow	p =1		p =1		p =1		p =1				

VMS: Visual Motion Sensitvity/Görsel Hareket Duyarlılığı (VMS); Ort: ortalama, ss: standart sapma, F: iki yönlü karma model ANOVA, F^b: Tekrarlayan Ölçümlerde ANOVA t testi, η^2; eta katsayısı, *p<0,05: istatistiksel anlamlılık vardır

Projeye dahil edilen DSBS'li katılımcılarda VMS- Optokinetik Hız Referansı ölçümlerinin değişimlerinde gruplar arasında istatistiksel olarak anlamlı farklılık bulunmadı (p>0.05).

Grup içi zamana göre karşılaştırmalarda;

- VR+BDT grubunda ölçümlerin zamana göre uygulama öncesi ve sonrası değişimi istatistiksel olarak anlamlı bulunmadı ($p>0.05$).
- VR grubunda ölçümlerin zamana göre uygulama öncesi ve sonrası değişimi istatistiksel olarak anlamlı bulunmadı ($p>0.05$).
- BDT grubunda ölçümlerin zamana göre uygulama öncesi ve sonrası değişimi istatistiksel olarak anlamlı bulunmadı ($p>0.05$).
- Aktif kontrol grubunda ölçümlerin zamana göre uygulama öncesi ve sonrası değişimi istatistiksel olarak anlamlı bulunmadı ($p>0.05$). (Bkz. Tablo 23).

Tablo 24. Visual Motion Sensitvity/Görsel Hareket Duyarlılığı (VMS) Testi Optokinetik Hız Referansı Parametresinin Gruplar Arasında ve Zaman Noktalarına Göre (Pre–Post–Follow-up) Karşılaştırılması.

Tablo 24. Visual Motion Sensitvity/Görsel Hareket Duyarlılığı (VMS) Testi Optokinetik Hız Ortalaması Parametresinin Gruplar Arasında ve Zamana Göre Karşılaştırılması.

**Değişken**	**VR+BDT**	**VR**	**BDT**	**Aktif Kontrol**	**Gruplar arası Karşılaştırma**	**Gruplararası Bağımsız Karşılaştırma**	
	**Ort ± ss**	**Ort ± ss**	**Ort ± ss**	**Ort ± ss**		**F^a**	**p^a**
**Pre-VMS optokinetic hız ort.**	24,82 ± 16,05	22,78 ± 12,08	20,8 ± 10,11	23,46 ± 11,32	F =1,336	F =0,205	p =0,892
**Post-VMS optokinetic. hız ort.**	13,23 ± 10,72	13,2 ± 6,2	14,18 ± 5,56	23,49 ± 8,77	p =0,25	F =4,833	p =0,006
**Follow-VMS optokinetic. hız ort.**	14,39 ± 9,24	16,08 ± 5,94	16,17 ± 5,39	23,48 ± 6,32	η =0,087	F =4,37	p =0,009
**Grupiçi Karşılaştırma (F^b)**	F =5,547 p =0,007	F =2,065 p =0,14	F =1,073 p =0,351	F =0 p =1			

Pre-Post	p =0,072	p =0,146	p =0,452	p =1
Pre-Follow	p =0,022	p =0,19	p =0,53	p =1
Post-Follow	p =1	p =0,192	p =0,533	p =1

VMS: Visual Motion Sensitvity/Görsel Hareket Duyarlılığı (VMS); Ort: ortalama, ss: standart sapma, F: iki yönlü karma model ANOVA, F^b: Tekrarlayan Ölçümlerde ANOVA t testi, η^2; eta katsayısı, *p<0,05: istatistiksel anlamlılık vardır

Projeye dahil edilen DSBS'li katılımcılarda VMS Optokinetik Hız Ortalaması ölçümlerinin değişimlerinde gruplar arasında istatistiksel olarak anlamlı farklılık bulunmadı (p>0.05).

Grup içi zamana göre karşılaştırmalarda;

- VR+BDT grubunda ölçümlerin zamana göre uygulama öncesi ve sonrası değişimi istatistiksel olarak anlamlı bulundu (p<0.05).
    - Pre ile follow arasındaki değişim istatistiksel olarak anlamlı bulundu (p<0.05).
- VR grubunda ölçümlerin zamana göre uygulama öncesi ve sonrası değişimi istatistiksel olarak anlamlı bulunmadı (p>0.05).
- BDT grubunda ölçümlerin zamana göre uygulama öncesi ve sonrası değişimi istatistiksel olarak anlamlı bulunmadı (p>0.05).
- Aktif kontrol grubunda ölçümlerin zamana göre uygulama öncesi ve sonrası değişimi istatistiksel olarak anlamlı bulunmadı (p>0.05). (Bkz. Tablo24)

Tablo 25. Visual Motion Sensitvity/Görsel Hareket Duyarlılığı (VMS) Testi Optokinetik Alan Referansı Parametresinin Gruplar Arasında ve Zamana Göre Karşılaştırılması.

Değişken	VR+BDT	VR	BDT	Aktif Kontrol	Gruplar arası Karşılaştırma	Gruplararası Bağımsız Karşılaştırma	
	Ort ± ss	Ort ± ss	Ort ± ss	Ort ± ss		F^a	p^a

**Pre-VMS optokinetik .alan ref.**	6,03 ± 4,01	3,25 ± 3,05	2,56 ± 1,93	5,56 ± 3,83	F =3,297	F =3,049	p =0,039
**Post-VMS optokinetik .alan ref.**	2,11 ± 1,65	1,47 ± 1,32	3,85 ± 2,9	2,84 ± 2,47	p =0,006	F =2,428	p =0,079
**Follow-VMS optokinetik .alan ref.**	2,5 ± 1,59	2 ± 1,13	3,46 ± 2,03	4,2 ± 1,69	η =0,191	F =4,131	p =0,012
**Grupiçi Karşılaştırma (F^b)**	F =14,904 p =0	F =1,013 p =0,372	F =0,579 p =0,565	F =4,816 p =0,013			
Pre-Post	p =0,02	p =0,542	p =0,927	p =0,088			
Pre-Follow	p =0,001	p =0,478	p =0,854	p =0,292			
Post-Follow	p =1	p =0,881	p =1	p =0,016			
**Pre-VMS optokinetik. alan ort.**	5,96 ± 5,59	10,83 ± 13,66	3,71 ± 3,06	20,8 ± 11,61	F =5,701	F =7,536	p =0
**Post-VMS optokinetik. alan ort.**	2,4 ± 1,62	4,78 ± 5,84	11,93 ± 8,63	9,49 ± 9,88	p =0	F =3,494	p =0,024
**Follow-VMS optokinetik. alan ort.**	2,76 ± 1,81	6,6 ± 4,84	9,46 ± 6,03	15,15 ± 8,46	η =0,294	F =8,159	p =0
**Grupiçi Karşılaştırma (F^b)**	F =2,716 p =0,078	F =1,841 p =0,172	F =3,704 p =0,033	F =14,473 p =0			
Pre-Post	p =1	p =0,314	p =0,07	p =0,005			
Pre-Follow	p =0,675	p =0,236	p =0,043	p =0,037			
Post-Follow	p =1	p =0,592	p =0,211	p =0			

Visual Motion Sensitvity/Görsel Hareket Duyarlılığı (VMS) Ort: ortalama, ss: standart sapma, F: iki yönlü karma model ANOVA, F^b: Tekrarlayan Ölçümlerde ANOVA t testi, η^2; eta katsayısı, *p<0,05: istatistiksel anlamlılık vardır

Projeye dahil edilen DSBS'li katılımcılarda VMS- Optokinetik Alan Ortalama ölçümlerinin değişimlerinde gruplar arasında istatistiksel olarak anlamlı farklılık bulundu (p<0,05). Gruplar arası zamana göre değişimde elde edilen kısmi eta kare (η^2) değeri 0.294, etkileşimin toplam varyansın yaklaşık %29,4'ünü açıkladığını göstermektedir. Grup zaman etkileşimi zamanın her grupta etkisinin aynı olmadığını göstermektedir.

Grup içi zamana göre karşılaştırmalarda;

- VR+BDT grubunda ölçümlerin zamana göre uygulama öncesi ve sonrası değişimi istatistiksel olarak anlamlı bulunmadı (p>0.05).
- VR grubunda ölçümlerin zamana göre uygulama öncesi ve sonrası değişimi istatistiksel olarak anlamlı bulunmadı (p>0.05).
- BDT grubunda ölçümlerin zamana göre uygulama öncesi ve sonrası değişimi istatistiksel olarak anlamlı bulundu (p<0.05).
    - Pre ile post arasındaki değişim istatistiksel olarak anlamlı bulundu (p<0.05).
- Aktif kontrol grubunda ölçümlerin zamana göre uygulama öncesi ve sonrası değişimi istatistiksel olarak anlamlı bulundu (p<0.05).
    - Pre ile post, pre ile follow, post ile follow arasındaki değişim istatistiksel olarak anlamlı bulundu (p<0.05). (Bkz.Tablo 26)

Tablo 27. Visual Motion Sensitvity/Görsel Hareket Duyarlılığı (VMS) Testi Optik Akış Hız Referansı Ortalaması Parametresinin Gruplar Arasında ve Zamana Göre Karşılaştırılması.

Değişken	VR+BDT		VR		BDT		Aktif Kontrol		Gruplar arası Karşılaştırma	Gruplararası Bağımsız Karşılaştırma	
	Ort ± ss		Ort ± ss		Ort ± ss		Ort ± ss			F^a	p^a
**Pre-VMS optik akış hız ref.**	27,73 ± 12,81		28,43 ± 25,17		22,52 ± 7,23		44,28 ± 31,17		F =1,326	F =2,403	p =0,081
**Post-VMS optik akış hız ref.**	2,62 ± 2,26		5,03 ± 12,44		4,23 ± 10,72		10,02 ± 7,82		p =0,255	F =1,568	p =0,212
**Follow-VMS optik akış hız ref.**	5,13 ± 2,81		12,05 ± 10,8		9,72 ± 7,6		27,15 ± 14,75		η =0,088	F =10,812	p =0
**Grupiçi Karşılaştırma**	F =40,936	p =0	F =6,929	p =0,003	F =5,645	p =0,007	F =20,057	p =0			

(F^b)				
Pre-Post	p =0,005	p =0,02	p =0,041	p =0
Pre-Follow	p =0	p =0,006	p =0,015	p =0,001
Post-Follow	p =1	p =0,146	p =0,222	p =0

VMS: Visual Motion Sensitvity/Görsel Hareket Duyarlılığı;Ort: ortalama, ss: standart sapma, F: iki yönlü karma model ANOVA, F^b: Tekrarlayan Ölçümlerde ANOVA t testi, η^2; eta katsayısı, *$p<0,05$: istatistiksel anlamlılık vardır

Projeye dahil edilen DSBS'li katılımcılarda VMS Optik Akış Hız Referansı ölçümlerinin değişimlerinde gruplar arasında istatistiksel olarak anlamlı farklılık bulunmadı ($p>0.05$).

Grup içi zamana göre karşılaştırmalarda;

- VR+BDT grubunda ölçümlerin zamana göre uygulama öncesi ve sonrası değişimi istatistiksel olarak anlamlı bulundu ($p<0.05$).
  - Pre ile post, pre ile follow arasındaki değişim istatistiksel olarak anlamlı bulundu ($p<0.05$).
- VR grubunda ölçümlerin zamana göre uygulama öncesi ve sonrası değişimi istatistiksel olarak anlamlı bulundu ($p<0.05$).
  - Pre ile post, pre ile follow arasındaki değişim istatistiksel olarak anlamlı bulundu ($p<0.05$).
- BDT grubunda ölçümlerin zamana göre uygulama öncesi ve sonrası değişimi istatistiksel olarak anlamlı bulundu ($p<0.05$).
  - Pre ile post, pre ile follow arasındaki değişim istatistiksel olarak anlamlı bulundu ($p<0.05$).
- Aktif kontrol grubunda ölçümlerin zamana göre uygulama öncesi ve sonrası değişimi istatistiksel olarak anlamlı bulundu ($p<0.05$).

- Pre ile post, pre ile follow, post ile follow arasındaki değişim istatistiksel olarak anlamlı bulundu ($p<0.05$). (Bkz Tablo 27).

Tablo 28. Visual Motion Sensitvity/Görsel Hareket Duyarlılığı (VMS) Testi Optik Akış Hız Ortalaması Parametresinin Gruplar Arasında ve Zamana Göre Karşılaştırılması.

Değişken	VR+BDT	VR	BDT	Aktif Kontrol	Gruplar arası Karşılaştırma	Gruplararası Bağımsız Karşılaştırma	
	Ort ± ss	Ort ± ss	Ort ± ss	Ort ± ss		F^a	p^a
**Pre-VMS optik akış hız ort.**	43,75 ± 14,64	36,68 ± 29,61	38,69 ± 22,98	36,13 ± 17,31	F =3,333	F =0,282	p =0,838
**Post-VMS optik akış hız ort.**	3,05 ± 2,17	3,29 ± 5,78	3,01 ± 4,22	15,32 ± 10,09	p =0,006	F =10,24	p =0
**Follow-VMS akış hız ort.**	7,12 ± 2,81	13,31 ± 12,52	13,71 ± 7,15	25,73 ± 10,19	η =0,208	F =9,569	p =0
**Grupiçi Karşılaştırma (F^b)**	F =90,07 p =0	F =9,545 p =0	F =18,68 p =0	F =18,306 p =0			
Pre-Post	p =0	p =0	p =0	p =0,003			
Pre-Follow	p =0	p =0	p =0	p =0,033			
Post-Follow	p =0,27	p =0,003	p =0	p =0			

VMS: Visual Motion Sensitvity/Görsel Hareket Duyarlılığı; Ort: ortalama, ss: standart sapma, F: iki yönlü karma model ANOVA, F^b: Tekrarlayan Ölçümlerde ANOVA t testi, η^2; eta katsayısı, *$p<0,05$: istatistiksel anlamlılık vardır

Projeye dahil edilen DSBS'li katılımcılarda VMS Optik Akış Hızı Ortalması değişimlerinde gruplar arasında istatistiksel olarak anlamlı farklılık bulundu ($p<0,05$). Gruplar arası zamana göre değişimde elde edilen kısmi eta kare (η^2) değeri 0.208, etkileşimin toplam varyansın yaklaşık %20.8'ini açıkladığını göstermektedir. Grup zaman etkileşimi zamanın her grupta etkisinin aynı olmadığını göstermektedir.

Grup içi zamana göre karşılaştırmalarda;

- VR+BDT grubunda ölçümlerin zamana göre uygulama öncesi ve sonrası değişimi istatistiksel olarak anlamlı bulundu ($p<0.05$).
    - Pre ile post, pre ile follow arasındaki değişim istatistiksel olarak anlamlı bulundu ($p<0.05$).
- VR grubunda ölçümlerin zamana göre uygulama öncesi ve sonrası değişimi istatistiksel olarak anlamlı bulundu ($p<0.05$).
    - Pre ile post, pre ile follow, post ile follow arasındaki değişim istatistiksel olarak anlamlı bulundu ($p<0.05$).
- BDT grubunda ölçümlerin zamana göre uygulama öncesi ve sonrası değişimi istatistiksel olarak anlamlı bulundu ($p<0.05$).
    - Pre ile post, pre ile follow, post ile follow arasındaki değişim istatistiksel olarak anlamlı bulundu ($p<0.05$).
- Aktif kontrol grubunda ölçümlerin zamana göre uygulama öncesi ve sonrası değişimi istatistiksel olarak anlamlı bulundu ($p<0.05$).
    - Pre ile post, pre ile follow, post ile follow arasındaki değişim istatistiksel olarak anlamlı bulundu ($p<0.05$).

Tablo 29. Visual Motion Sensitvity/Görsel Hareket Duyarlılığı (VMS) Testi Optik Akış Alan Referansı Parametresinin Gruplar Arasında ve Zamana Göre Karşılaştırılması.

**Değişken**	**VR+BDT**	**VR**	**BDT**	**Aktif Kontrol**	**Gruplar arası Karşılaştırma**	**Gruplararası Bağımsız Karşılaştırma**	
	**Ort ± ss**	**Ort ± ss**	**Ort ± ss**	**Ort ± ss**		**F^a**	**p^a**
**Pre-VMS optik akış alan ref.**	6,79 ± 5,99	3,99 ± 2,98	3,24 ± 1,45	8,01 ± 6,03	F =0,691	F =2,575	p =0,068
**Post-VMS optik akış alan ref.**	3,27 ± 2,95	2,38 ± 1,99	1,71 ± 0,76	7,52 ± 5,08	p =0,658	F =7,365	p =0,001
**Follow-VMS optik akış**	3,62 ± 2,71	2,86 ± 1,43	2,17 ± 0,56	7,76 ± 4,27	η =0,052	F =9,494	p =0

**alan ref.**								
**Grupiçi Karşılaştırma (F^b)**	F =5,183	p =0,01	F =0,339	p =0,715	F =0,419	p =0,661	F =0,088	p =0,916
Pre-Post	p =0,156		p =1		p =1		p =1	
Pre-Follow	p =0,038		p =1		p =1		p =1	
Post-Follow	p =1		p =1		p =1		p =1	

VMS: Visual Motion Sensitvity/Görsel Hareket Duyarlılığı; Ort: ortalama, ss: standart sapma, F: iki yönlü karma model ANOVA, F^b: Tekrarlayan Ölçümlerde ANOVA t testi, η^2; eta katsayısı, *p<0,05: istatistiksel anlamlılık vardır

Projeye dahil edilen DSBS'li katılımcılarda VMS Optik Akış Alan Referansı ölçümlerinin değişimlerinde gruplar arasında istatistiksel olarak anlamlı farklılık bulunmadı (p>0.05).

Grup içi zamana göre karşılaştırmalarda;

- VR+BDT grubunda ölçümlerin zamana göre uygulama öncesi ve sonrası değişimi istatistiksel olarak anlamlı bulundu (p<0.05).
  - Pre ile follow arasındaki değişim istatistiksel olarak anlamlı bulundu (p<0.05).
- VR grubunda ölçümlerin zamana göre uygulama öncesi ve sonrası değişimi istatistiksel olarak anlamlı bulunmadı (p>0.05).
- BDT grubunda ölçümlerin zamana göre uygulama öncesi ve sonrası değişimi istatistiksel olarak anlamlı bulunmadı (p>0.05).
- Aktif kontrol grubunda ölçümlerin zamana göre uygulama öncesi ve sonrası değişimi istatistiksel olarak anlamlı bulunmadı (p>0.05). (Bkz.Tablo 29).

Tablo 30. Visual Motion Sensitvity/Görsel Hareket Duyarlılığı (VMS) Testi Optik Akış Alan Ortalaması Parametresinin Gruplar Arasında ve Zamana Göre Karşılaştırılması.

Değişken	VR+BDT	VR	BDT	Aktif Kontrol	Gruplar arası Karşılaştırma	Gruplararası Bağımsız Karşılaştırma

	Ort ± ss	Ort ± ss	Ort ± ss	Ort ± ss		F^a	p^a
**Pre-VMS optik akış alan ort.**	5,74 ± 3,78	20,62 ± 20,92	9,36 ± 7,15	29,12 ± 15,77	F =0,675	F =7,774	p =0
**Post-VMS optik akış alan ort.**	3,65 ± 2,81	8,53 ± 9,22	3,59 ± 2,22	24,1 ± 11,66	p =0,67	F =18,022	p =0
**Follow-VMS optik akış alan ort.**	3,86 ± 2,57	12,16 ± 9,36	5,32 ± 1,62	26,61 ± 10,85	η =0,055	F =23,172	p =0
**Grupiçi Karşılaştırma (F^b)**	F =0,89 p =0,42	F =3,842 p =0,031	F =1,459 p =0,247	F =1,492 p =0,239			
Pre-Post	p =1	p =0,128	p =0,61	p =0,619			
Pre-Follow	p =1	p =0,065	p =0,441	p =0,9			
Post-Follow	p =1	p =0,406	p =1	p =0,384			

VMS: Visual Motion Sensitvity/Görsel Hareket Duyarlılığı; Ort: ortalama, ss: standart sapma, F: iki yönlü karma model ANOVA, F^b: Tekrarlayan Ölçümlerde ANOVA t testi, η^2; eta katsayısı, *p<0,05: istatistiksel anlamlılık vardır

Projeye dahil edilen DSBS'li katılımcılarda VMS) Testi Optik Akış Alan Ortalaması ölçümlerinin değişimlerinde gruplar arasında istatistiksel olarak anlamlı farklılık bulunmadı (p>0.05).

Grup içi zamana göre karşılaştırmalarda;

- VR+BDT grubunda ölçümlerin zamana göre uygulama öncesi ve sonrası değişimi istatistiksel olarak anlamlı bulunmadı (p>0.05).
- VR grubunda ölçümlerin zamana göre uygulama öncesi ve sonrası değişimi istatistiksel olarak anlamlı bulunmadı (p>0.05).
- BDT grubunda ölçümlerin zamana göre uygulama öncesi ve sonrası değişimi istatistiksel olarak anlamlı bulunmadı (p>0.05).
- Aktif kontrol grubunda ölçümlerin zamana göre uygulama öncesi ve sonrası değişimi istatistiksel olarak anlamlı bulunmadı (p>0.05). (Bkz.Tablo 30).

Tablo 31. Visual Motion Sensitvity/Görsel Hareket Duyarlılığı (VMS) Testi Optokinetik Ortalama Hız Referansı Parametresinin Gruplar Arasında ve Zamana Göre Karşılaştırılması.

**Değişken**	**VR+BDT**	**VR**	**BDT**	**Aktif Kontrol**	**Gruplar arası Karşılaştırma**	**Gruplararası Bağımsız Karşılaştırma**	
	**Ort ± ss**	**Ort ± ss**	**Ort ± ss**	**Ort ± ss**		**F^a**	**p^a**
**Pre-VMS optokinetik ortalama hız ref.**	17,4 ± 13,94	8,59 ± 3,23	12,08 ± 4,37	24,54 ± 17,37	F =1,247	F =4,367	p =0,009
**Post-VMS optokinetik ortalama hız ref.**	7,29 ± 6,67	9,4 ± 11,13	7,88 ± 2,82	24,43 ± 16,36	p =0,291	F =6,837	p =0,001
**Follow-VMS optokinetik ortalama hız ref.**	8,3 ± 6,34	9,16 ± 8,05	9,14 ± 2,24	24,49 ± 12,6	η =0,084	F =10,482	p =0
**Grupiçi Karşılaştırma (F^b)**	F =8,858 p =0,001	F =0,021 p =0,98	F =0,617 p =0,545	F =0,001 p =0,999			
Pre-Post	p =0,152	p =1	p =1	p =1			
Pre-Follow	p =0,018	p =1	p =0,859	p =1			
Post-Follow	p =1	p =1	p =1	p =1			

VMS: Visual Motion Sensitvity/Görsel Hareket Duyarlılığı;Ort: ortalama, ss: standart sapma, F: iki yönlü karma model ANOVA, F^b: Tekrarlayan Ölçümlerde ANOVA t testi, η^2; eta katsayısı, *p<0,05: istatistiksel anlamlılık vardır

Projeye dahil edilen DSBS'li katılımcılarda VMS Optokinetik Ortalama Hız Referansı .ölçümlerinin değişimlerinde gruplar arasında istatistiksel olarak anlamlı farklılık bulunmadı (p>0.05).

Grup içi zamana göre karşılaştırmalarda;

- VR+BDT grubunda ölçümlerin zamana göre uygulama öncesi ve sonrası değişimi istatistiksel olarak anlamlı bulundu (p<0.05).

- Pre ile follow arasındaki değişim istatistiksel olarak anlamlı bulundu (p<0.05).

- VR grubunda ölçümlerin zamana göre uygulama öncesi ve sonrası değişimi istatistiksel olarak anlamlı bulunmadı (p>0.05).
- BDT grubunda ölçümlerin zamana göre uygulama öncesi ve sonrası değişimi istatistiksel olarak anlamlı bulunmadı (p>0.05).
- Aktif kontrol grubunda ölçümlerin zamana göre uygulama öncesi ve sonrası değişimi istatistiksel olarak anlamlı bulunmadı (p>0.05). (Bkz.Tablo 31).

Tablo 32. Visual Motion Sensitvity/Görsel Hareket Duyarlılığı (VMS) Testi Optokinetik Ortalama Hız Parametresinin Gruplar Arasında ve Zamana Göre Karşılaştırılması.

Değişken	VR+BDT		VR		BDT		Aktif Kontrol		Gruplar arası Karşılaştırma	Gruplararası Bağımsız Karşılaştırma	
	Ort ± ss		Ort ± ss		Ort ± ss		Ort ± ss			F^a	p^a
**Pre-VMS optokinetik ortalma hızı**	16,59 ± 11,26		13,3 ± 7,8		9,51 ± 5,39		13,14 ± 6,43		F =2,617	F =1,511	p =0,226
**Post-VMS optokinetik ortalma hızı**	8,31 ± 4,97		8,63 ± 3,14		6,85 ± 2,95		13,08 ± 4,93		p =0,022	F =5,393	p =0,003
**Follow-VMS optokinetik ortalma hızı**	9,14 ± 5,49		10,03 ± 3,49		7,65 ± 2,42		13,11 ± 3,63		η =0,157	F =4,535	p =0,008
**Grupiçi Karşılaştırma (F^b)**	F =20,57	p =0	F =2,094	p =0,136	F =0,74	p =0,483	F =0,001	p =0,999			
Pre-Post	p =0,005		p =0,154		p =0,719		p =1				
Pre-Follow	p =0		p =0,134		p =0,676		p =1				
Post-Follow	p =1		p =0,327		p =1		p =1				
**Pre-VMS optik akış hız ort.**	21,5 ± 7,23		16,51 ± 14,85		18,98 ± 9,97		29,82 ± 14,94		F =3,57	F =2,472	p =0,077

**Post-VMS optik akış hız ort.**	4,8 ± 3,45	11,46 ± 10,58	9,25 ± 4,24	29,77 ± 9,94	p =0,004	F =23,299	p =0
**Follow-VMS optik akış hız ort.**	6,47 ± 2,93	12,97 ± 11,26	12,17 ± 2,74	29,8 ± 9,39	η =0,225	F =20,706	p =0
**Grupiçi Karşılaştırma (F^b)**	F =39,732 p =0	F =0,826 p =0,446	F =4,214 p =0,023	F =0 p =1			
Pre-Post	p =0,002	p =0,852	p =0,057	p =1			
Pre-Follow	p =0	p =0,695	p =0,028	p =1			
Post-Follow	p =1	p =1	p =0,244	p =1			

VMS: Visual Motion Sensitvity/Görsel Hareket Duyarlılığı; Ort: ortalama, ss: standart sapma, F: iki yönlü karma model ANOVA, F^b: Tekrarlayan Ölçümlerde ANOVA t testi, η^2; eta katsayısı, *p<0,05: istatistiksel anlamlılık vardır

Projeye dahil edilen DSBS'li katılımcılarda VMS) Testi Optik Akış Hızı Ortalaması ölçümlerinin değişimlerinde gruplar arasında istatistiksel olarak anlamlı farklılık bulundu ($p<0,05$). Gruplar arası zamana göre değişimde elde edilen kısmi eta kare (η^2) değeri 0.225, etkileşimin toplam varyansın yaklaşık %25.2'sini açıkladığını göstermektedir. Grup zaman etkileşimi zamanın her grupta etkisinin aynı olmadığını göstermektedir.

Grup içi zamana göre karşılaştırmalarda;

- VR+BDT grubunda ölçümlerin zamana göre uygulama öncesi ve sonrası değişimi istatistiksel olarak anlamlı bulundu ($p<0.05$).
    - Pre ile post, pre ile follow arasındaki değişim istatistiksel olarak anlamlı bulundu ($p<0.05$).
- VR grubunda ölçümlerin zamana göre uygulama öncesi ve sonrası değişimi istatistiksel olarak anlamlı bulunmadı ($p>0.05$).
- BDT grubunda ölçümlerin zamana göre uygulama öncesi ve sonrası değişimi istatistiksel olarak anlamlı bulundu ($p<0.05$).
    - pre ile follow arasındaki değişim istatistiksel olarak anlamlı bulundu ($p<0.05$).

- Aktif kontrol grubunda ölçümlerin zamana göre uygulama öncesi ve sonrası değişimi istatistiksel olarak anlamlı bulunmadı (p>0.05). (Bkz Tablo 34).

Tablo 35. Visual Motion Sensitvity/Görsel Hareket Duyarlılığı (VMS) Testi Salınım Elips Alanı Referansı Parametresinin Gruplar Arasında ve Zamana Göre Karşılaştırılması.

Değişken	VR+BDT	VR	BDT	Aktif Kontrol	Gruplar arası Karşılaştırma	Gruplararası Bağımsız Karşılaştırma	
	Ort ± ss	Ort ± ss	Ort ± ss	Ort ± ss		F^a	p^a
**Pre-VMS salınım elips alanı ref**	6,51 ± 5,75	11,07 ± 28,81	9,31 ± 23,85	52,39 ± 38,17	F =4,42	F =7,363	p =0
**Post-VMS salınım elips alanı ref ref**	2,73 ± 2,49	3,51 ± 7,13	4,23 ± 10,72	10,02 ± 7,82	p =0,001	F =2,125	p =0,112
**Follow-VMS salınım elips alanı ref**	3,1 ± 2,55	5,78 ± 10,5	5,75 ± 9,65	31,2 ± 17,76	η =0,244	F =14,634	p =0
**Grupiçi Karşılaştırma (F^b)**	F =0,699 p =0,503	F =0,721 p =0,493	F =0,355 p =0,703	F =21,707 p =0			
Pre-Post	p =1	p =1	p =1	p =0			
Pre-Follow	p =1	p =1	p =1	p =0			
Post-Follow	p =1	p =1	p =1	p =0			

VMS: Visual Motion Sensitvity/Görsel Hareket Duyarlılığı; Ort: ortalama, ss: standart sapma, F: iki yönlü karma model ANOVA, F^b: Tekrarlayan Ölçümlerde ANOVA t testi, η^2; eta katsayısı, *p<0,05: istatistiksel anlamlılık vardır

Projeye dahil edilen DSBS'li katılımcılarda VMS Testi Salınım Elips Alanı Referansı ölçümlerinin değişimlerinde gruplar arasında istatistiksel olarak anlamlı farklılık bulundu (p<0,05). Gruplar arası zamana göre değişimde elde edilen kısmi eta kare (η^2) değeri 0.244, etkileşimin toplam varyansın yaklaşık %24.4'ünü açıkladığını göstermektedir. Grup zaman etkileşimi zamanın her grupta etkisinin aynı olmadığını göstermektedir.

Grup içi zamana göre karşılaştırmalarda;

- VR+BDT grubunda ölçümlerin zamana göre uygulama öncesi ve sonrası değişimi istatistiksel olarak anlamlı bulunmadı (p>0.05).
- VR grubunda ölçümlerin zamana göre uygulama öncesi ve sonrası değişimi istatistiksel olarak anlamlı bulunmadı (p>0.05).
- BDT grubunda ölçümlerin zamana göre uygulama öncesi ve sonrası değişimi istatistiksel olarak anlamlı bulunmadı (p>0.05).
- Aktif kontrol grubunda ölçümlerin zamana göre uygulama öncesi ve sonrası değişimi istatistiksel olarak anlamlı bulunmadı (p>0.05). (Bkz Tablo 35).

Tablo 36. Visual Motion Sensitvity/Görsel Hareket Duyarlılığı (VMS) Testi Salınım Elips Alanı Parametresinin Gruplar Arasında ve Zamana Göre Karşılaştırılması.

**Değişken**	**VR+BDT**	**VR**	**BDT**	**Aktif Kontrol**	**Gruplar arası Karşılaştırma**	**Gruplararası Bağımsız Karşılaştırma**	
	**Ort ± ss**	**Ort ± ss**	**Ort ± ss**	**Ort ± ss**		**F^a**	**p^a**
**Pre-VMS salınım elips alanı ort.**	6,05 ± 5,38	8,85 ± 13,43	4,39 ± 4,91	16,57 ± 11,73	F =0,214	F =3,709	p =0,019
**Post-VMS salınım elips alanı ort.**	3,14 ± 2,28	4,4 ± 6,01	3,01 ± 4,22	15,32 ± 10,09	p =0,971	F =9,749	p =0
**Follow-VMS salınım elips alanı ort.**	3,44 ± 2,28	5,74 ± 5,74	3,42 ± 3,28	15,94 ± 7,99	η =0,015	F =14,25	p =0
**Grupiçi Karşılaştırma (F^b)**	F =2,033 p =0,144	F =1,25 p =0,297	F =0,132 p =0,877	F =0,143 p =0,868			
Pre-Post	p =1	p =0,641	p =1	p =1			
Pre-Follow	p =0,856	p =0,488	p =1	p =1			
Post-Follow	p =1	p =1	p =1	p =1			

VMS: Visual Motion Sensitvity/Görsel Hareket Duyarlılığı; Ort: ortalama, ss: standart sapma, F: iki yönlü karma model ANOVA, F^b: Tekrarlayan Ölçümlerde ANOVA t testi, $η^2$; eta katsayısı, *p<0,05: istatistiksel anlamlılık vardır

Projeye dahil edilen DSBS'li katılımcılarda (VMS) Testi Salınım Elips Alanı ölçümlerinin değişimlerinde gruplar arasında istatistiksel olarak anlamlı farklılık bulunmadı (p>0.05).

Grup içi zamana göre karşılaştırmalarda;

- VR+BDT grubunda ölçümlerin zamana göre uygulama öncesi ve sonrası değişimi istatistiksel olarak anlamlı bulunmadı (p>0.05).
- VR grubunda ölçümlerin zamana göre uygulama öncesi ve sonrası değişimi istatistiksel olarak anlamlı bulunmadı (p>0.05).
- BDT grubunda ölçümlerin zamana göre uygulama öncesi ve sonrası değişimi istatistiksel olarak anlamlı bulunmadı (p>0.05).
- Aktif kontrol grubunda ölçümlerin zamana göre uygulama öncesi ve sonrası değişimi istatistiksel olarak anlamlı bulunmadı (p>0.05). (Bkz Tablo 36).

Tablo 37. Visual Motion Sensitvity/Görsel Hareket Duyarlılığı (VMS) Optik Akış Salınım Alanı Referansı Parametresinin Gruplar Arasında ve Zamana Göre Karşılaştırılması.

Değişken	VR+BDT	VR	BDT	Aktif Kontrol	Gruplar arası Karşılaştırma	Gruplararası Bağımsız Karşılaştırma	
	Ort ± ss	Ort ± ss	Ort ± ss	Ort ± ss		F^a	p^a
**Pre-VMS optik akış salınım alanı ref.**	5,72 ± 5,26	4,22 ± 2,87	3,73 ± 2,61	4,88 ± 3,46	F =1,098	F =0,586	p =0,628
**Post-VMS optik akış**	2,61 ± 2,6	2,18 ± 1,94	2,25 ± 1,74	4,8 ± 3,06	p =0,371	F =3,171	p =0,035

**salınım alanı ref.**							
**Follow-VMS optik akış salınım alanı ref.**	2,92 ± 2,38	2,79 ± 1,4	2,69 ± 1,2	4,84 ± 2,79	η =0,078	F =2,921	p =0,046
**Grupiçi Karşılaştırma (F^b)**	F =5,082 p =0,011	F =1,127 p =0,335	F =0,791 p =0,461	F =0,008 p =0,993			
Pre-Post	p =0,094	p =0,453	p =0,682	p =1			
Pre-Follow	p =0,034	p =0,551	p =0,791	p =1			
Post-Follow	p =1	p =0,448	p =0,676	p =1			

VMS: Visual Motion Sensitvity/Görsel Hareket Duyarlılığı; Ort: ortalama, ss: standart sapma, F: iki yönlü karma model ANOVA, F^b: Tekrarlayan Ölçümlerde ANOVA t testi, η^2; eta katsayısı, *p<0,05: istatistiksel anlamlılık vardır

Projeye dahil edilen DSBS'li katılımcılarda VMS) Optik Akış Salınım Elips Alanı Referansı ölçümlerinin değişimlerinde gruplar arasında istatistiksel olarak anlamlı farklılık bulunmadı (p>0.05).

Grup içi zamana göre karşılaştırmalarda;

- VR+BDT grubunda ölçümlerin zamana göre uygulama öncesi ve sonrası değişimi istatistiksel olarak anlamlı bulundu (p<0.05).
  - pre ile follow arasındaki değişim istatistiksel olarak anlamlı bulundu (p<0.05).
- VR grubunda ölçümlerin zamana göre uygulama öncesi ve sonrası değişimi istatistiksel olarak anlamlı bulunmadı (p>0.05).
- BDT grubunda ölçümlerin zamana göre uygulama öncesi ve sonrası değişimi istatistiksel olarak anlamlı bulunmadı (p>0.05).
- Aktif kontrol grubunda ölçümlerin zamana göre uygulama öncesi ve sonrası değişimi istatistiksel olarak anlamlı bulunmadı (p>0.05). (Bkz Tablo 36).

Tablo 37. Visual Motion Sensitvity/Görsel Hareket Duyarlılığı (VMS) Optik Akış Salınım Alanı Parametresinin Gruplar Arasında ve Zamana Göre Karşılaştırılması.

**Değişken**	**VR+BDT**	**VR**	**BDT**	**Aktif Kontrol**	**Gruplar arası Karşılaştırma**	**Gruplararası Bağımsız Karşılaştırma**	
	**Ort ± ss**	**Ort ± ss**	**Ort ± ss**	**Ort ± ss**		**F^a**	**p^a**
**Pre-VMS optik akış salınım alanı ort.**	6,13 ± 3,8	26,86 ± 22,12	12,21 ± 10,29	24,53 ± 15,75	F =2,176	F =4,581	p =0,008
**Post-VMS optik akış salınım alanı ort.**	2,76 ± 2,6	9,36 ± 9,23	5,15 ± 4,23	22,11 ± 12,27	p =0,055	F =12,218	p =0
**Follow-VMS optik akış salınım alanı ort.**	3,1 ± 2,27	14,61 ± 10,18	7,27 ± 4,18	23,32 ± 12,17	η =0,15	F =12,017	p =0
**Grupiçi Karşılaştırma (F^b)**	F =1,825 p =0,176	F =8,048 p =0,001	F =1,8 p =0,18	F =0,519 p =0,599			
Pre-Post	p =1	p =0,004	p =0,317	p =1			
Pre-Follow	p =0,978	p =0,002	p =0,243	p =1			
Post-Follow	p =1	p =0,024	p =0,58	p =1			

VMS: Visual Motion Sensitvity/Görsel Hareket Duyarlılığı; Ort: ortalama, ss: standart sapma, F: iki yönlü karma model ANOVA, F^b: Tekrarlayan Ölçümlerde ANOVA t testi, η^2; eta katsayısı, *p<0,05: istatistiksel anlamlılık vardır

Projeye dahil edilen DSBS'li katılımcılarda VMS) Optik Akış Salınım Alanı ölçümlerinin değişimlerinde gruplar arasında istatistiksel olarak anlamlı farklılık bulunmadı (p>0.05).

Grup içi zamana göre karşılaştırmalarda;

- VR+BDT grubunda ölçümlerin zamana göre uygulama öncesi ve sonrası değişimi istatistiksel olarak anlamlı bulunmadı (p>0.05).

- VR grubunda ölçümlerin zamana göre uygulama öncesi ve sonrası değişimi istatistiksel olarak anlamlı bulundu (p<0.05).
  - Pre ile post, pre ile follow, post ile follow arasındaki değişim istatistiksel olarak anlamlı bulundu (p<0.05).
- BDT grubunda ölçümlerin zamana göre uygulama öncesi ve sonrası değişimi istatistiksel olarak anlamlı bulunmadı (p>0.05).
- Aktif kontrol grubunda ölçümlerin zamana göre uygulama öncesi ve sonrası değişimi istatistiksel olarak anlamlı bulunmadı (p>0.05). (Bkz Tablo 37).

Tablo 38. DSBS'li Hastalarda cVEMP P1 Latansının Zamana Göre Değişimi ve Grup Etkisi

**Değişken**	**VR+BDT**	**VR**	**BDT**	**Aktif Kontrol**	**Gruplar arası Karşılaştırma**	**Gruplararası Bağımsız Karşılaştırma**	
	**Ort ± ss**	**Ort ± ss**	**Ort ± ss**	**Ort ± ss**		**F^a**	**p^a**
**Pre-cVEMP P1 latans (ms)**	13,37 ± 0,74	12,44 ± 0,99	12,73 ± 0,87	12,51 ± 0,89	F =2,879	F =2,603	p =0,064
**Post-cVEMP P1 latans (ms)**	12,51 ± 0,95	12,78 ± 0,76	13 ± 1,01	12,87 ± 1,15	p =0,013	F =0,494	p =0,688
**Follow-cVEMP P1 latans (ms)**	12,6 ± 0,9	12,68 ± 0,55	12,92 ± 0,91	12,69 ± 0,62	η =0,167	F =0,388	p =0,762
**Grupiçi Karşılaştırma (F^b)**	F =14,698 p =0	F =0,584 p =0,562	F =0,407 p =0,668	F =0,953 p =0,394			
Pre-Post	p =0,075	p =1	p =1	p =0,911			
Pre-Follow	p =0,006	p =0,928	p =1	p =1			
Post-Follow	p =1	p =1	p =1	p =0,614			

Ort: ortalama, ss: standart sapma, F: iki yönlü karma model ANOVA, F^b: Tekrarlayan Ölçümlerde ANOVA t testi, η^2; eta katsayısı, *p<0,05: istatistiksel anlamlılık vardır

Projeye dahil edilen DSBS'li katılımcılarda cVEMP P1 ölçümlerinin değişimlerinde gruplar arasında istatistiksel olarak anlamlı farklılık bulundu ($p<0,05$). Gruplar arası zamana göre değişimde elde edilen kısmi eta kare (η^2) değeri 0.167, etkileşimin toplam varyansın yaklaşık %16.7'sini açıkladığını göstermektedir. Grup zaman etkileşimi zamanın her grupta etkisinin aynı olmadığını göstermektedir.

Grup içi zamana göre karşılaştırmalarda;

- VR+BDT grubunda ölçümlerin zamana göre uygulama öncesi ve sonrası değişimi istatistiksel olarak anlamlı bulundu ($p<0.05$).
    - pre ile follow arasındaki değişim istatistiksel olarak anlamlı bulundu ($p<0.05$).
- VR grubunda ölçümlerin zamana göre uygulama öncesi ve sonrası değişimi istatistiksel olarak anlamlı bulunmadı ($p>0.05$).
- BDT grubunda ölçümlerin zamana göre uygulama öncesi ve sonrası değişimi istatistiksel olarak anlamlı bulunmadı ($p>0.05$).
- Aktif kontrol grubunda ölçümlerin zamana göre uygulama öncesi ve sonrası değişimi istatistiksel olarak anlamlı bulunmadı ($p>0.05$). (Bkz Tablo 38).

Tablo 39. DSBS'li Hastalarda cVEMP N1 Latansının Zamana Göre Değişimi ve Grup Etkisi

Değişken	VR+BDT	VR	BDT	Aktif Kontrol	Gruplar arası Karşılaştırma	Gruplararası Bağımsız Karşılaştırma	
	Ort ± ss	Ort ± ss	Ort ± ss	Ort ± ss		F^a	p^a
Pre-cVEMP N1 latans (ms)	22,7 ± 1,31	23,14 ± 1,06	23,49 ± 1,46	22,8 ± 1,93	F =0,528	F =0,681	p =0,569
Post-cVEMP N1 latans (ms)	23,26 ± 1,86	23,07 ± 0,96	23,22 ± 1,41	23,3 ± 1,69	p =0,786	F =0,047	p =0,986

**Follow-cVEMP N1 latans (ms)**	23,2 ± 1,67		23,09 ± 0,74		23,3 ± 1,02		23,05 ± 1,55		η =0,036	F =0,091	p =0,965
**Grupiçi Karşılaştırma (F^b)**	F =1,1	p =0,342	F =0,005	p =0,995	F =0,114	p =0,892	F =1,211	p =0,308			
Pre-Post	p =1		p =1		p =1		p =1				
Pre-Follow	p =0,757		p =1		p =1		p =1				
Post-Follow	p =1		p =1		p =1		p =0,504				

Ort: ortalama, ss: standart sapma, F: iki yönlü karma model ANOVA, F^b: Tekrarlayan Ölçümlerde ANOVA t testi, η^2; eta katsayısı, *$p<0,05$: istatistiksel anlamlılık vardır

Projeye dahil edilen DSBS'li katılımcılarda cVEMP N1 ölçümlerinin değişimlerinde gruplar arasında istatistiksel olarak anlamlı farklılık bulunmadı ($p>0.05$).

Grup içi zamana göre karşılaştırmalarda;

- VR+BDT grubunda ölçümlerin zamana göre uygulama öncesi ve sonrası değişimi istatistiksel olarak anlamlı bulunmadı ($p>0.05$).
- VR grubunda ölçümlerin zamana göre uygulama öncesi ve sonrası değişimi istatistiksel olarak anlamlı bulunmadı ($p>0.05$).
- BDT grubunda ölçümlerin zamana göre uygulama öncesi ve sonrası değişimi istatistiksel olarak anlamlı bulunmadı ($p>0.05$).
- Aktif kontrol grubunda ölçümlerin zamana göre uygulama öncesi ve sonrası değişimi istatistiksel olarak anlamlı bulunmadı ($p>0.05$). (Bkz Tablo 38).

Tablo 40. DSBS'li Hastalarda cVEMP P1N1 Amplitüd Değerlerinin Zamana Göre Değişimi ve Grup Etkisi

Değişken	VR+BDT		VR		BDT		Aktif Kontrol		Gruplar arası Karşılaştırma	Gruplararası Bağımsız Karşılaştırma	
	Ort ± ss		Ort ± ss		Ort ± ss		Ort ± ss			F^a	p^a
**Pre-cVEMP P1N1 amplitüd (µV)**	153,03 ± 21,05		140,66 ± 21,56		154,79 ± 33,92		151,52 ± 30,81		F =0,494	F =0,59	p =0,625
**Post-cVEMP P1N1 amplitüd (µV)**	139,39 ± 30,38		135,45 ± 35,59		162,08 ± 30,34		149,39 ± 33,66		p =0,811	F =1,543	p =0,217
**Follow-cVEMP P1N1 amplitüd (µV)**	140,75 ± 27,66		137,01 ± 27,52		159,9 ± 24,99		150,45 ± 15,72		η =0,033	F =2,098	p =0,115
**Grupiçi Karşılaştırma (F^b)**	F =2,282	p =0,115	F =0,091	p =0,913	F =0,194	p =0,824	F =0,029	p =0,972			
Pre-Post	p =0,921		p =1		p =1		p =1				
Pre-Follow	p =0,484		p =1		p =1		p =1				
Post-Follow	p =1		p =1		p =1		p =1				

Ort: ortalama, ss: standart sapma, F: iki yönlü karma model ANOVA, F^b: Tekrarlayan Ölçümlerde ANOVA t testi, η^2; eta katsayısı, *p<0,05: istatistiksel anlamlılık vardır

Projeye dahil edilen DSBS'li katılımcılarda **cVEMP P1N1** ölçümlerinin değişimlerinde gruplar arasında istatistiksel olarak anlamlı farklılık bulunmadı (p>0.05).

Grup içi zamana göre karşılaştırmalarda;

- VR+BDT grubunda ölçümlerin zamana göre uygulama öncesi ve sonrası değişimi istatistiksel olarak anlamlı bulunmadı (p>0.05).
- VR grubunda ölçümlerin zamana göre uygulama öncesi ve sonrası değişimi istatistiksel olarak anlamlı bulunmadı (p>0.05).
- BDT grubunda ölçümlerin zamana göre uygulama öncesi ve sonrası değişimi istatistiksel olarak anlamlı bulunmadı (p>0.05).

- Aktif kontrol grubunda ölçümlerin zamana göre uygulama öncesi ve sonrası değişimi istatistiksel olarak anlamlı bulunmadı (p>0.05). (Bkz Tablo 40).

Tablo 41. DSBS'li Hastalarda cVEMP Eşik Değerlerinin Zamana Göre Değişimi ve Grup Etkisi

**Değişken**	**VR+BDT**		**VR**		**BDT**		**Aktif Kontrol**		**Gruplar arası Karşılaştırma**	**Gruplararası Bağımsız Karşılaştırma**	
	**Ort ± ss**		**Ort ± ss**		**Ort ± ss**		**Ort ± ss**			**F[a]**	**p[a]**
**Pre-cVEMP Eşik (dB nHL)**	89,77 ± 3,62		90,02 ± 2,88		89,52 ± 2,44		89,58 ± 3,65		F =0,212	F =0,057	p =0,982
**Post-cVEMP Eşik (dB nHL)**	90,65 ± 2,94		90,48 ± 2,4		89,39 ± 3,85		89,22 ± 2,37		p =0,972	F =0,734	p =0,538
**Follow-cVEMP Eşik (dB nHL)**	90,57 ± 2,69		90,34 ± 1,47		89,43 ± 2,91		89,4 ± 2,37		η =0,015	F =0,728	p =0,541
**Grupiçi Karşılaştırma (F[b])**	F =0,662	p =0,521	F =0,063	p =0,939	F =0,005	p =0,995	F =0,147	p =0,863			
Pre-Post	p =1		p =1		p =1		p =1				
Pre-Follow	p =1		p =1		p =1		p =1				
Post-Follow	p =1		p =1		p =1		p =1				

Ort: ortalama, ss: standart sapma, F: iki yönlü karma model ANOVA, F[b]: Tekrarlayan Ölçümlerde ANOVA t testi, η^2; eta katsayısı, *p<0,05: istatistiksel anlamlılık vardır

Projeye dahil edilen DSBS'li katılımcılarda cVEMP Eşik ölçümlerinin değişimlerinde gruplar arasında istatistiksel olarak anlamlı farklılık bulunmadı (p>0.05).

Grup içi zamana göre karşılaştırmalarda;

- VR+BDT grubunda ölçümlerin zamana göre uygulama öncesi ve sonrası değişimi istatistiksel olarak anlamlı bulunmadı (p>0.05).

- VR grubunda ölçümlerin zamana göre uygulama öncesi ve sonrası değişimi istatistiksel olarak anlamlı bulunmadı ($p>0.05$).
- BDT grubunda ölçümlerin zamana göre uygulama öncesi ve sonrası değişimi istatistiksel olarak anlamlı bulunmadı ($p>0.05$).
- Aktif kontrol grubunda ölçümlerin zamana göre uygulama öncesi ve sonrası değişimi istatistiksel olarak anlamlı bulunmadı ($p>0.05$). (Bkz Tablo 41).

Tablo 42. DSBS'li Hastalarda cVEMP Yanıtları Arasındaki Amplitüd Asimetrisi Değerlerinin Zamana Göre Değişimi ve Grup Etkisi

Değişken	VR+BDT		VR		BDT		Aktif Kontrol		Gruplar arası Karşılaştırma	Gruplararası Bağımsız Karşılaştırma	
	Ort ± ss		Ort ± ss		Ort ± ss		Ort ± ss			F^a	p^a
**Pre-cVEMP IAR (%)**	21,72 ± 7,96		22,92 ± 7,85		18,21 ± 6,45		18,18 ± 5,38		F =3,744	F =1,443	p =0,243
**Post-cVEMP IAR (%)**	21,87 ± 8,06		14,59 ± 5,64		21,89 ± 10,36		23,87 ± 6,76		p =0,002	F =3,045	p =0,039
**Follow-cVEMP IAR (%)**	21,86 ± 6,99		17,09 ± 5,49		20,79 ± 7,36		21,03 ± 4,69		η =0,207	F =1,3	p =0,287
**Grupiçi Karşılaştırma (F^b)**	F =0,003	p =0,997	F =3,695	p =0,033	F =0,789	p =0,461	F =7,595	p =0,002			
Pre-Post	p =1		p =0,036		p =0,697		p =0,175				
Pre-Follow	p =1		p =0,059		p =0,805		p =0,616				
Post-Follow	p =1		p =0,029		p =0,656		p =0,005				

IAR: Interaural Asymmetry Ratio / kulaklararası yanıtları arasındaki amplitüd asimetrisi Ort: ortalama, ss: standart sapma, F: iki yönlü karma model ANOVA, F^b: Tekrarlayan Ölçümlerde ANOVA t testi, η^2; eta katsayısı, *$p<0,05$: istatistiksel anlamlılık vardır

Projeye dahil edilen DSBS'li katılımcılarda cVEMP Yanıtları Arasındaki Amplitüd Asimetrisi / IAR ölçümlerinin değişimlerinde gruplar arasında istatistiksel olarak anlamlı farklılık bulundu (p<0,05). Gruplar arası zamana göre değişimde elde edilen kısmi eta kare (η^2) değeri 0.207, etkileşimin toplam varyansın yaklaşık %20.7'sini açıkladığını göstermektedir. Grup zaman etkileşimi zamanın her grupta etkisinin aynı olmadığını göstermektedir.

Grup içi zamana göre karşılaştırmalarda;

- VR+BDT grubunda ölçümlerin zamana göre uygulama öncesi ve sonrası değişimi istatistiksel olarak anlamlı bulunmadı (p>0.05).
- VR grubunda ölçümlerin zamana göre uygulama öncesi ve sonrası değişimi istatistiksel olarak anlamlı bulundu (p<0.05).
    - Pre ile post, post ile follow arasındaki değişim istatistiksel olarak anlamlı bulundu (p<0.05).
- BDT grubunda ölçümlerin zamana göre uygulama öncesi ve sonrası değişimi istatistiksel olarak anlamlı bulunmadı (p>0.05).
- Aktif kontrol grubunda ölçümlerin zamana göre uygulama öncesi ve sonrası değişimi istatistiksel olarak anlamlı bulundu (p<0.05).
    - Post ile follow arasındaki değişim istatistiksel olarak anlamlı bulundu (p<0.05). (Bkz Tablo 42).

Tablo 43. DSBS'li Hastalarda oVEMP P1 Latans Değerlerinin Zamana Göre Değişimi ve Grup Etkisi

Değişken	VR+BDT	VR	BDT	Aktif Kontrol	Gruplar arası Karşılaştırma	Gruplararası Bağımsız Karşılaştırma	
	Ort ± ss	Ort ± ss	Ort ± ss	Ort ± ss		F[a]	p[a]
**Pre-oVEMP P1 latans (ms)**	15,61 ± 1,32	15,2 ± 1,12	14,79 ± 0,8	15,22 ± 1,1	F =0,788	F =1,064	p =0,374
**Post-oVEMP P1 latans**	15,14 ± 0,86	15,02 ± 0,75	15,08 ± 0,77	15,6 ± 1,1	p =0,582	F =1,115	p =0,353

Değişken	VR+BDT	VR	BDT	Aktif Kontrol	Gruplar arası		
(ms)							
**Follow-oVEMP P1 latans (ms)**	15,18 ± 0,78	15,07 ± 0,47	14,99 ± 0,59	15,41 ± 0,65	η =0,052	F =1,031	p =0,388
**Grupiçi Karşılaştırma (F^b)**	F =1,488 p =0,237	F =0,076 p =0,927	F =0,201 p =0,818	F =0,868 p =0,427			
Pre-Post	p =0,954	p =1	p =1	p =1			
Pre-Follow	p =0,598	p =1	p =1	p =1			
Post-Follow	p =1	p =1	p =1	p =0,679			

Ort: ortalama, ss: standart sapma, F: iki yönlü karma model ANOVA, F^b: Tekrarlayan Ölçümlerde ANOVA t testi, η^2; eta katsayısı, *p<0,05: istatistiksel anlamlılık vardır

Projeye dahil edilen DSBS'li katılımcılarda oVEMP P1 Latans ölçümlerinin değişimlerinde gruplar arasında istatistiksel olarak anlamlı farklılık bulunmadı (p>0.05).

Grup içi zamana göre karşılaştırmalarda;

- VR+BDT grubunda ölçümlerin zamana göre uygulama öncesi ve sonrası değişimi istatistiksel olarak anlamlı bulunmadı (p>0.05).
- VR grubunda ölçümlerin zamana göre uygulama öncesi ve sonrası değişimi istatistiksel olarak anlamlı bulunmadı (p>0.05).
- BDT grubunda ölçümlerin zamana göre uygulama öncesi ve sonrası değişimi istatistiksel olarak anlamlı bulunmadı (p>0.05).
- Aktif kontrol grubunda ölçümlerin zamana göre uygulama öncesi ve sonrası değişimi istatistiksel olarak anlamlı bulunmadı (p>0.05). (Bkz Tablo 43).

Tablo 44. DSBS'li Hastalarda oVEMP N1 Latans Değerlerinin Zamana Göre Değişimi ve Grup Etkisi

Değişken	VR+BDT	VR	BDT	Aktif Kontrol	Gruplar arası	Gruplararası Bağımsız

					Karşılaştırma	Karşılaştırma	
	**Ort ± ss**	**Ort ± ss**	**Ort ± ss**	**Ort ± ss**		**F^a**	**p^a**
**Pre-oVEMP N1 latans (ms)**	9,88 ± 0,93	10,26 ± 1,09	9,61 ± 1,41	10,31 ± 1,2	F =0,487	F =0,943	p =0,428
**Post-oVEMP N1 latans (ms)**	10,06 ± 0,83	10,28 ± 1,04	10,04 ± 0,98	10,1 ± 0,96	p =0,817	F =0,143	p =0,934
**Follow-oVEMP N1 latans (ms)**	10,04 ± 0,79	10,27 ± 0,66	9,91 ± 0,98	10,2 ± 0,89	η =0,033	F =0,437	p =0,728
**Grupiçi Karşılaştırma (F^b)**	F =0,43 p =0,653	F =0,001 p =0,999	F =0,647 p =0,529	F =0,47 p =0,629			
Pre-Post	p =1	p =1	p =0,805	p =1			
Pre-Follow	p =1	p =1	p =0,771	p =1			
Post-Follow	p =1	p =1	p =1	p =1			

Ort: ortalama, ss: standart sapma, F: iki yönlü karma model ANOVA, F^b: Tekrarlayan Ölçümlerde ANOVA t testi, η^2; eta katsayısı, *p<0,05: istatistiksel anlamlılık vardır

Projeye dahil edilen DSBS’li katılımcılarda oVEMP N1 ölçümlerinin değişimlerinde gruplar arasında istatistiksel olarak anlamlı farklılık bulunmadı (p>0.05).

Grup içi zamana göre karşılaştırmalarda;

- VR+BDT grubunda ölçümlerin zamana göre uygulama öncesi ve sonrası değişimi istatistiksel olarak anlamlı bulunmadı (p>0.05).
- VR grubunda ölçümlerin zamana göre uygulama öncesi ve sonrası değişimi istatistiksel olarak anlamlı bulunmadı (p>0.05).
- BDT grubunda ölçümlerin zamana göre uygulama öncesi ve sonrası değişimi istatistiksel olarak anlamlı bulunmadı (p>0.05).

- Aktif kontrol grubunda ölçümlerin zamana göre uygulama öncesi ve sonrası değişimi istatistiksel olarak anlamlı bulunmadı (p>0.05). (Bkz Tablo 44).

Tablo 45. DSBS'li Hastalarda oVEMP P1N1 Amplitüd Değerlerinin Zamana Göre Değişimi ve Grup Etkisi

**Değişken**	**VR+BDT**		**VR**		**BDT**		**Aktif Kontrol**		**Gruplar arası Karşılaştırma**	**Gruplararası Bağımsız Karşılaştırma**	
	**Ort ± ss**		**Ort ± ss**		**Ort ± ss**		**Ort ± ss**			**F^a**	**p^a**
**Pre-oVEMP P1N1 amplitüd (µV)**	10,32 ± 3,52		10,17 ± 2,09		11,23 ± 1,76		10,13 ± 2,76		F =1,313	F =0,476	p =0,701
**Post-oVEMP P1N1 amplitüd (µV)**	12,55 ± 1,7		10,13 ± 2,32		11,45 ± 2,24		9,88 ± 2,75		p =0,26	F =3,332	p =0,028
**Follow-oVEMP P1N1 amplitüd (µV)**	12,32 ± 1,51		10,14 ± 1,94		11,39 ± 1,64		10,01 ± 1,82		η =0,084	F =4,613	p =0,007
**Grupiçi Karşılaştırma (F^b)**	F =5,57	p =0,007	F =0,001	p =0,999	F =0,022	p =0,978	F =0,07	p =0,933			
Pre-Post	p =0,126		p =1		p =1		p =1				
Pre-Follow	p =0,032		p =1		p =1		p =1				
Post-Follow	p =1		p =1		p =1		p =1				

Ort: ortalama, ss: standart sapma, F: iki yönlü karma model ANOVA, F^b: Tekrarlayan Ölçümlerde ANOVA t testi, η^2; eta katsayısı, *p<0,05: istatistiksel anlamlılık vardır

Projeye dahil edilen DSBS'li katılımcılarda oVEMP P1N1 Amplitüd lateraller ölçümlerinin değişimlerinde gruplar arasında istatistiksel olarak anlamlı farklılık bulunmadı (p>0.05).

Grup içi zamana göre karşılaştırmalarda;

- VR+BDT grubunda ölçümlerin zamana göre uygulama öncesi ve sonrası değişimi istatistiksel olarak anlamlı bulundu ($p<0.05$).
    - pre ile follow arasındaki değişim istatistiksel olarak anlamlı bulundu ($p<0.05$).
- VR grubunda ölçümlerin zamana göre uygulama öncesi ve sonrası değişimi istatistiksel olarak anlamlı bulunmadı ($p>0.05$).
- BDT grubunda ölçümlerin zamana göre uygulama öncesi ve sonrası değişimi istatistiksel olarak anlamlı bulunmadı ($p>0.05$).
- Aktif kontrol grubunda ölçümlerin zamana göre uygulama öncesi ve sonrası değişimi istatistiksel olarak anlamlı bulunmadı ($p>0.05$). (Bkz Tablo 45).

Tablo 46. DSBS'li Hastalarda oVEMP Eşik Değerlerinin Zamana Göre Değişimi ve Grup Etkisi

**Değişken**	**VR+BDT**		**VR**		**BDT**		**Aktif Kontrol**		**Gruplar arası Karşılaştırma**	**Gruplararası Bağımsız Karşılaştırma**	
	**Ort ± ss**		**Ort ± ss**		**Ort ± ss**		**Ort ± ss**			**F^a**	**p^a**
**Pre-oVEMP Eşik (dB nHL)**	90,82 ± 2,98		89,29 ± 1,81		89,55 ± 2,55		89,1 ± 2,86		F =1,282	F =1,008	p =0,398
**Post-oVEMP Eşik (dB nHL)**	89,75 ± 3,14		90,8 ± 2,13		91,11 ± 2,73		88,32 ± 3,16		p =0,274	F =2,445	p =0,077
**Follow-oVEMP Eşik (dB nHL)**	89,85 ± 2,73		90,35 ± 1,62		90,64 ± 1,82		88,71 ± 1,94		η =0,082	F =2,129	p =0,11
**Grupiçi Karşılaştırma (F^b)**	F =0,863	p =0,429	F =0,671	p =0,517	F =0,78	p =0,465	F =0,533	p =0,591			
Pre-Post	p =1		p =0,743		p =0,639		p =1				
Pre-Follow	p =0,906		p =0,778		p =0,673		p =1				
Post-Follow	p =1		p =0,924		p =0,816		p =1				

Ort: ortalama, ss: standart sapma, F: iki yönlü karma model ANOVA, F^b: Tekrarlayan Ölçümlerde ANOVA t testi, η^2; eta katsayısı, *p<0,05: istatistiksel anlamlılık vardır

Projeye dahil edilen DSBS'li katılımcılarda oVEMP Eşik Değerlerinin ölçümlerinin değişimlerinde gruplar arasında istatistiksel olarak anlamlı farklılık bulunmadı (p>0.05).

Grup içi zamana göre karşılaştırmalarda;

- VR+BDT grubunda ölçümlerin zamana göre uygulama öncesi ve sonrası değişimi istatistiksel olarak anlamlı bulunmadı (p>0.05).
- VR grubunda ölçümlerin zamana göre uygulama öncesi ve sonrası değişimi istatistiksel olarak anlamlı bulunmadı (p>0.05).
- BDT grubunda ölçümlerin zamana göre uygulama öncesi ve sonrası değişimi istatistiksel olarak anlamlı bulunmadı (p>0.05).
- Aktif kontrol grubunda ölçümlerin zamana göre uygulama öncesi ve sonrası değişimi istatistiksel olarak anlamlı bulunmadı (p>0.05). (Bkz Tablo 46).

Tablo 47. DSBS'li Hastalarda oVEMP Yanıtları Arasındaki Amplitüd Asimetrisi Değerlerinin Zamana Göre Değişimi ve Grup Etkisi

Değişken	VR+BDT		VR		BDT		Aktif Kontrol		Gruplar arası Karşılaştırma	Gruplararası Bağımsız Karşılaştırma	
	Ort ± ss		Ort ± ss		Ort ± ss		Ort ± ss			F^a	p^a
Pre-oVEMP IAR (%)	22,01 ± 6,02		24,49 ± 11,03		20,49 ± 10,79		19,38 ± 9,06		F =0,451	F =0,641	p =0,593
Post-oVEMP IAR (%)	18,61 ± 9,87		25,63 ± 6,89		21,96 ± 8,87		21,6 ± 10,08		p =0,842	F =1,108	p =0,356
Follow-oVEMP IAR (%)	18,95 ± 9,02		25,29 ± 5,11		21,52 ± 6,6		20,49 ± 7,36		η =0,031	F =1,578	p =0,209
Grupiçi	F =1,486	p =0,238	F =0,041	p =0,96	F =0,075	p =0,928	F =0,57	p =0,57			

**Karşılaştırma (F^b)**				
Pre-Post	p =1	p =1	p =1	p =1
Pre-Follow	p =0,811	p =1	p =1	p =1
Post-Follow	p =1	p =1	p =1	p =1

IAR: Interaural Asymmetry Ratio / kulaklararası yanıtları arasındaki amplitüd asimetrisi ; Ort: ortalama, ss: standart sapma, F: iki yönlü karma model ANOVA, F^b: Tekrarlayan Ölçümlerde ANOVA t testi, η^2; eta katsayısı, *p<0,05: istatistiksel anlamlılık vardır

Projeye dahil edilen DSBS'li katılımcılarda oVEMP Yanıtları Arasındaki Amplitüd Asimetrisi ölçümlerinin değişimlerinde gruplar arasında istatistiksel olarak anlamlı farklılık bulunmadı (p>0.05).

Grup içi zamana göre karşılaştırmalarda;

- VR+BDT grubunda ölçümlerin zamana göre uygulama öncesi ve sonrası değişimi istatistiksel olarak anlamlı bulunmadı (p>0.05).
- VR grubunda ölçümlerin zamana göre uygulama öncesi ve sonrası değişimi istatistiksel olarak anlamlı bulunmadı (p>0.05).
- BDT grubunda ölçümlerin zamana göre uygulama öncesi ve sonrası değişimi istatistiksel olarak anlamlı bulunmadı (p>0.05).
- Aktif kontrol grubunda ölçümlerin zamana göre uygulama öncesi ve sonrası değişimi istatistiksel olarak anlamlı bulunmadı (p>0.05). (Bkz Tablo 47).
