# Supplementary material for: Treatment-related modulation of visuo-vestibular integration in post-earthquake dizziness syndrome: a longitudinal virtual reality–based study
Source: J Neurol. 2026 Mar 24;273(4):230. doi: 10.1007/s00415-026-13767-4 (PMC13013104; doi:10.1007/s00415-026-13767-4)
Supplement: Supplementary file 3 — Supplementary file3 (DOCX 296 KB) [file 415_2026_13767_MOESM3_ESM.docx]

**Sample Size**

The adequacy of the sample size is supported by a power analysis. Power analysis estimates the statistical power of the sample size (Faul et al., 2009). The sample size of this study was determined using power analysis. According to the calculation performed with the G*Power 3.1 software, with an effect size of 0.35, a significance level of 0.05, a confidence level of 0.95, and a statistical power of 0.95, the required sample size was calculated as at least 48 participants (12 per group) (Faul et al., 2009). In the present study, the sample size was planned to be increased by three additional participants per group to account for potential dropouts, missing data, or other unforeseen circumstances.


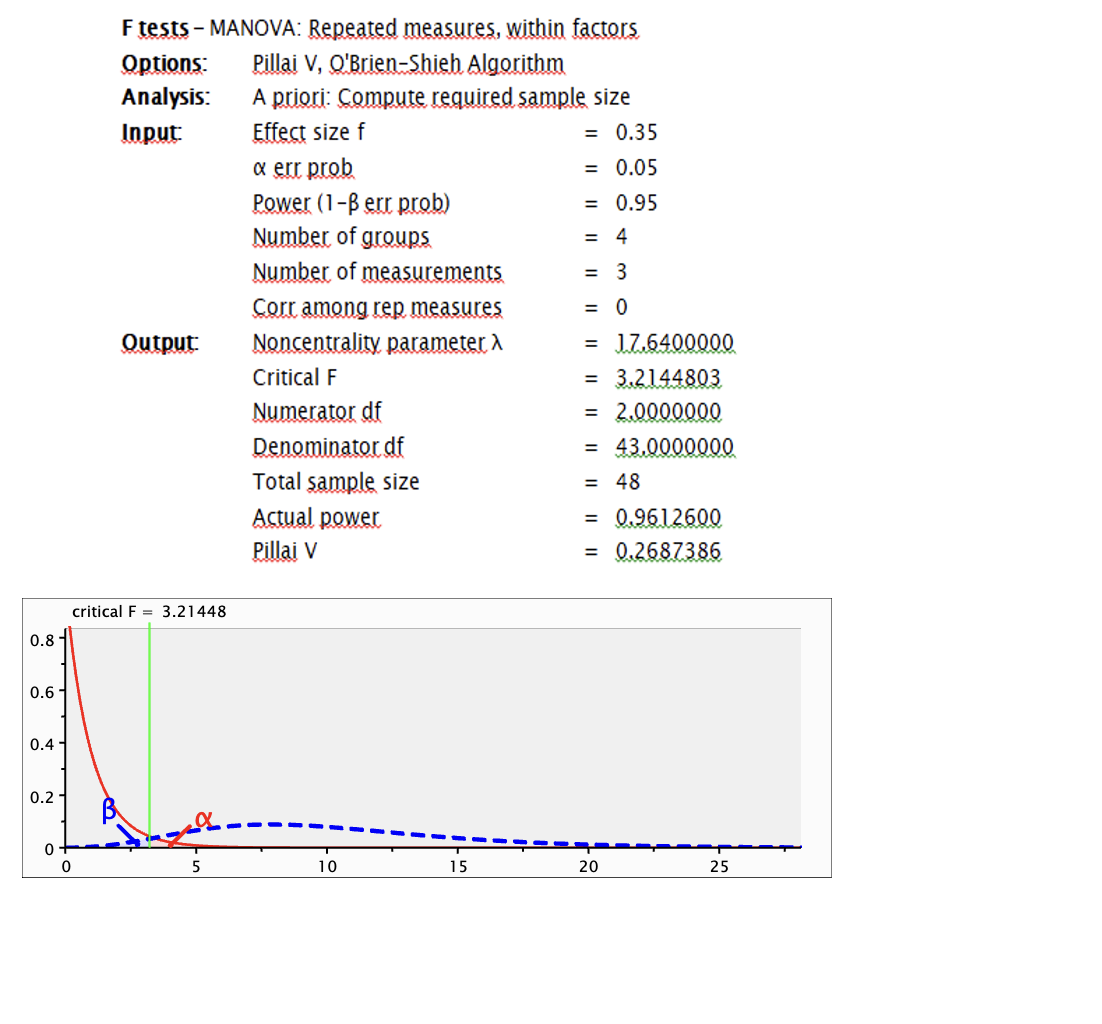


Reference

Faul, F., Erdfelder, E., Buchner, A. & Lang, A.-G. (2009). Statistical power analyses using G*Power 3.1: Tests for correlation and regression analyses. Behavior Research Methods, 41(4), 1149–1160. DOI: 10.3758/BRM.41.4.1149
